# Supplementary material for: One-pot aminobenzylation of aldehydes with toluenes
Source: Nat Commun. 2018 Aug 22;9:3365. doi: 10.1038/s41467-018-05638-y (PMC6105668; doi:10.1038/s41467-018-05638-y)
Supplement: Supplementary file 1 — Supplementary Information [file 41467_2018_5638_MOESM1_ESM.pdf]

One-pot Aminobenzylation of Aldehydes with Toluenes

Wang *et al.*

Supplementary Figure 1. ^{1}H NMR Spectrum of 3aa (400 MHz, $CDCl_3$)

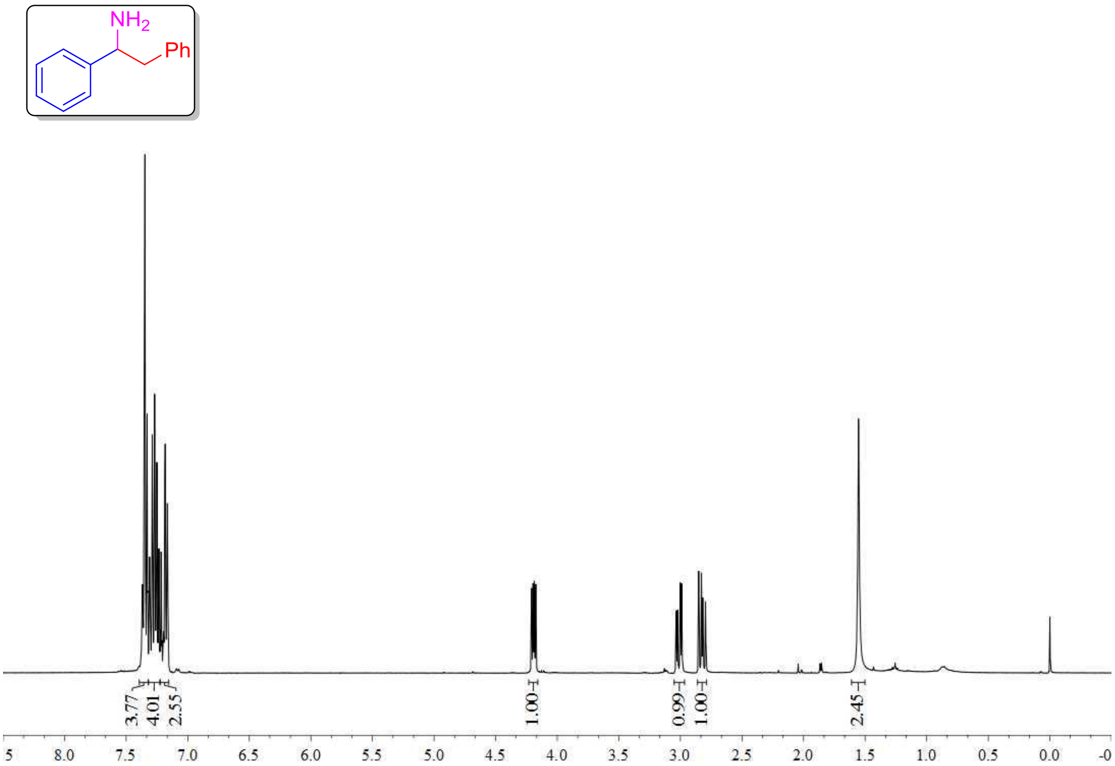

Supplementary Figure 2. ^{13}C NMR Spectrum of 3aa (101 MHz, $CDCl_3$)

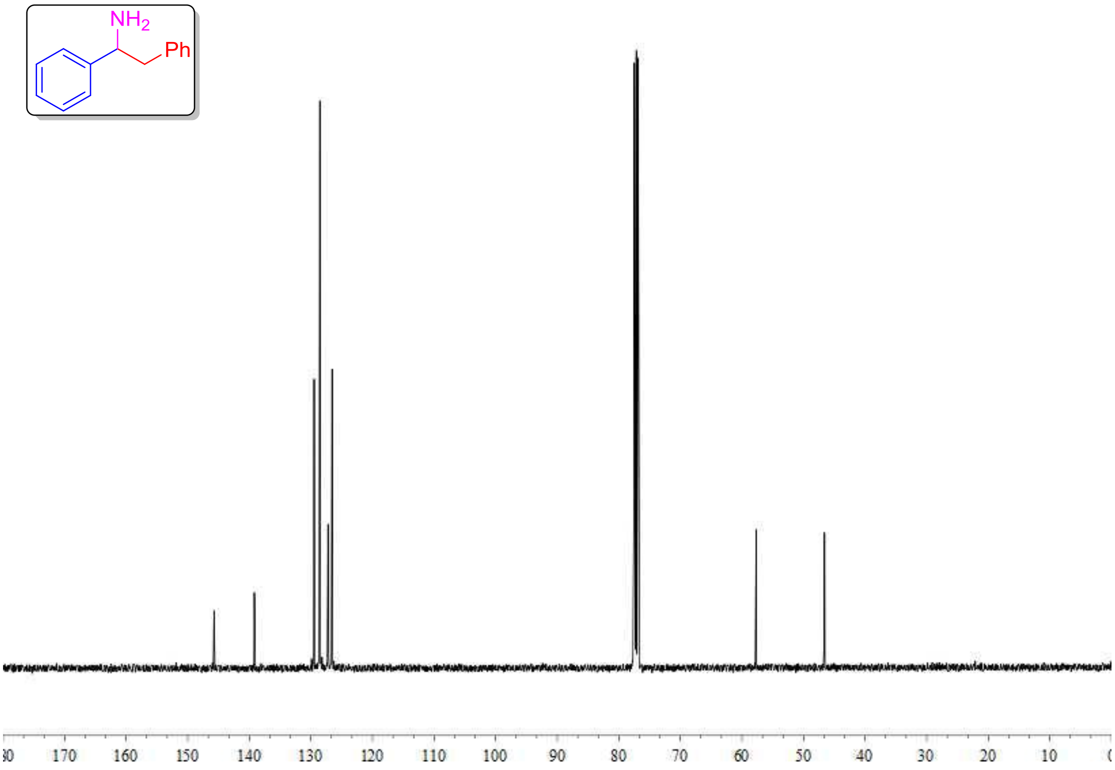

Supplementary Figure 3. ^{1}H NMR Spectrum of 3ba (400 MHz, $CDCl_3$)

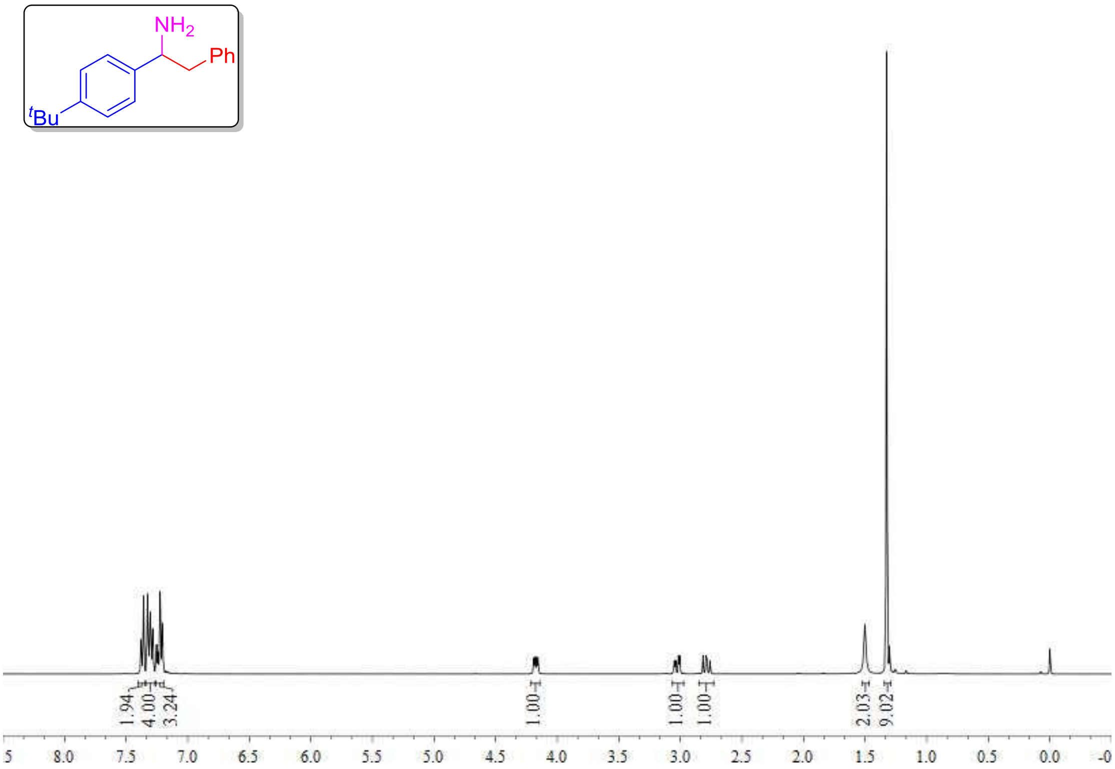

Supplementary Figure 4. ^{13}C NMR Spectrum of 3ba (101 MHz, $CDCl_3$)

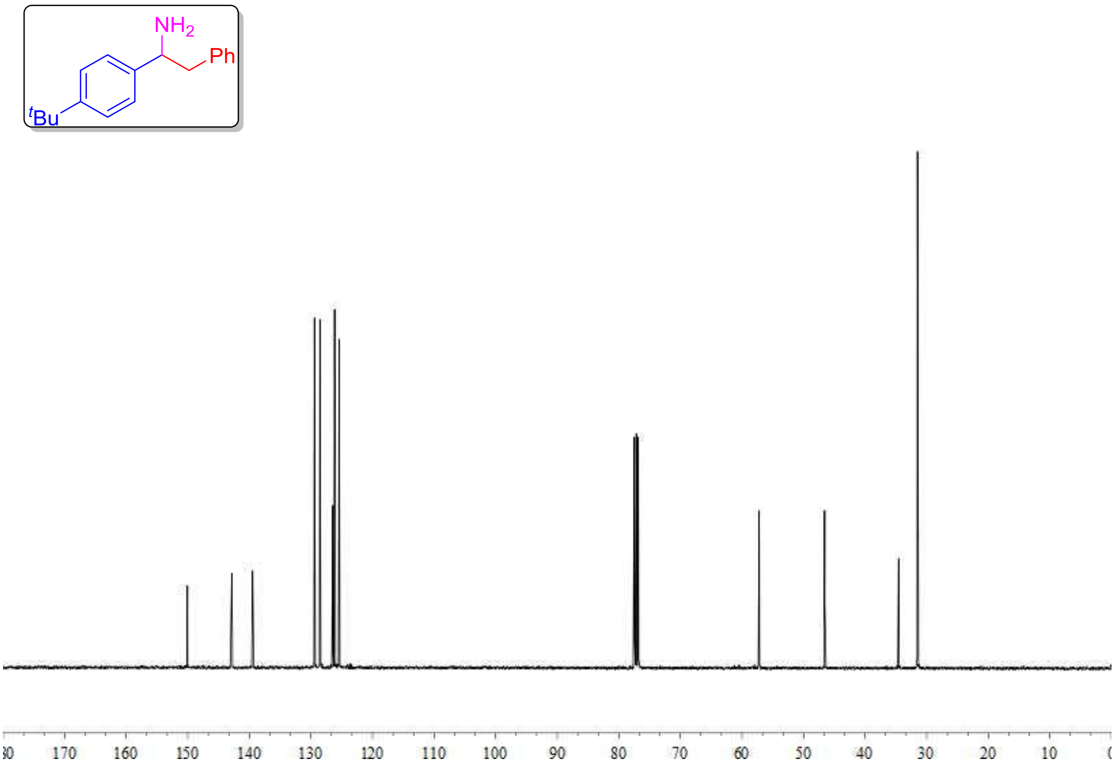

Supplementary Figure 5. ^{1}**H NMR Spectrum of 3ca (400 MHz,** $CDCl_3$**)**

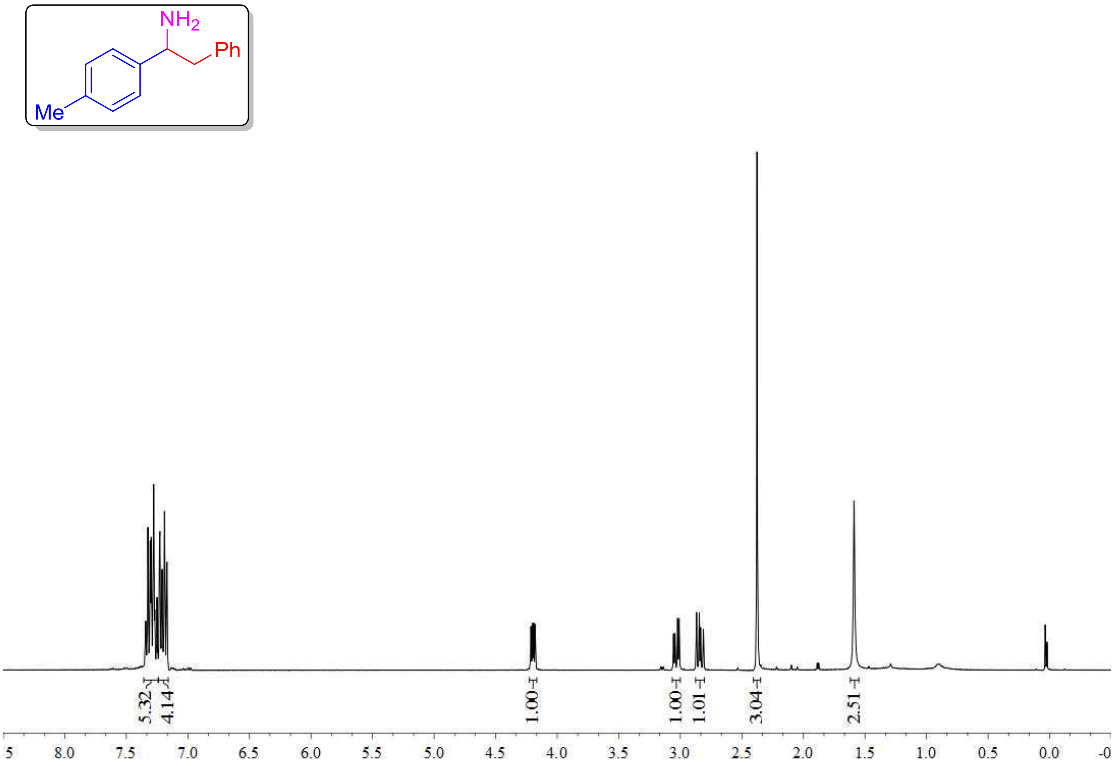

Supplementary Figure 6. ^{13}**C NMR Spectrum of 3ca (101 MHz,** $CDCl_3$**)**

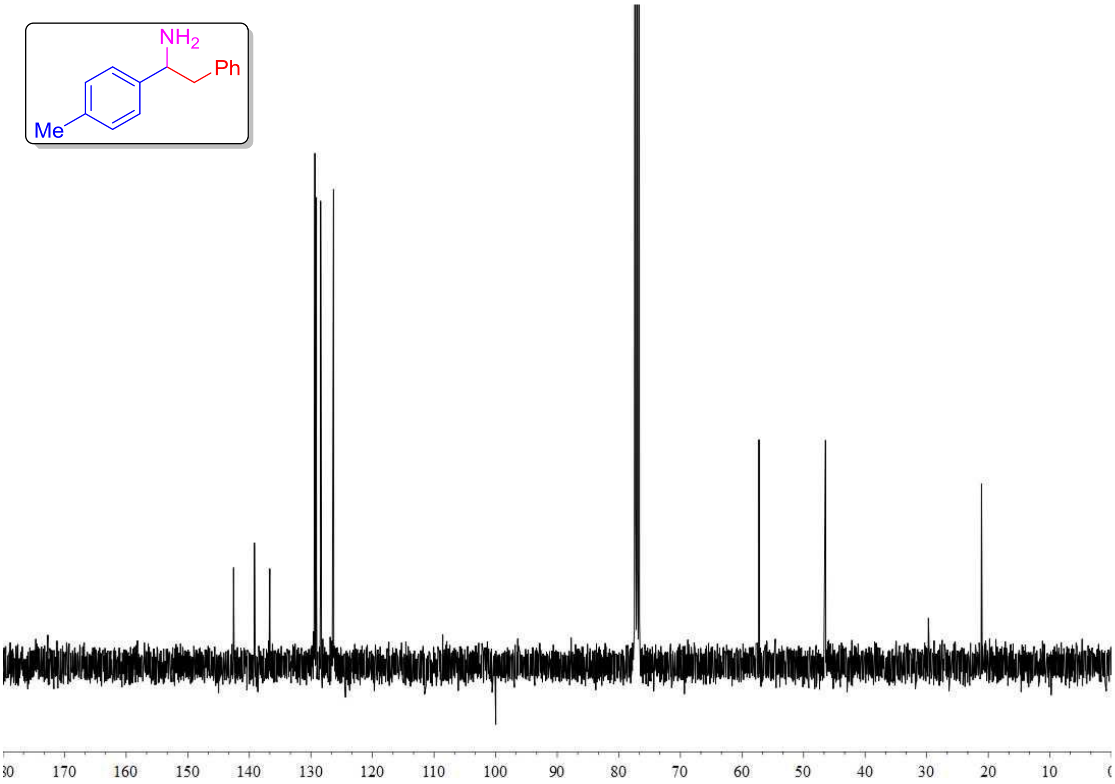

Supplementary Figure 7. **^{1}H NMR Spectrum of 3da (400 MHz, $CDCl_3$)**

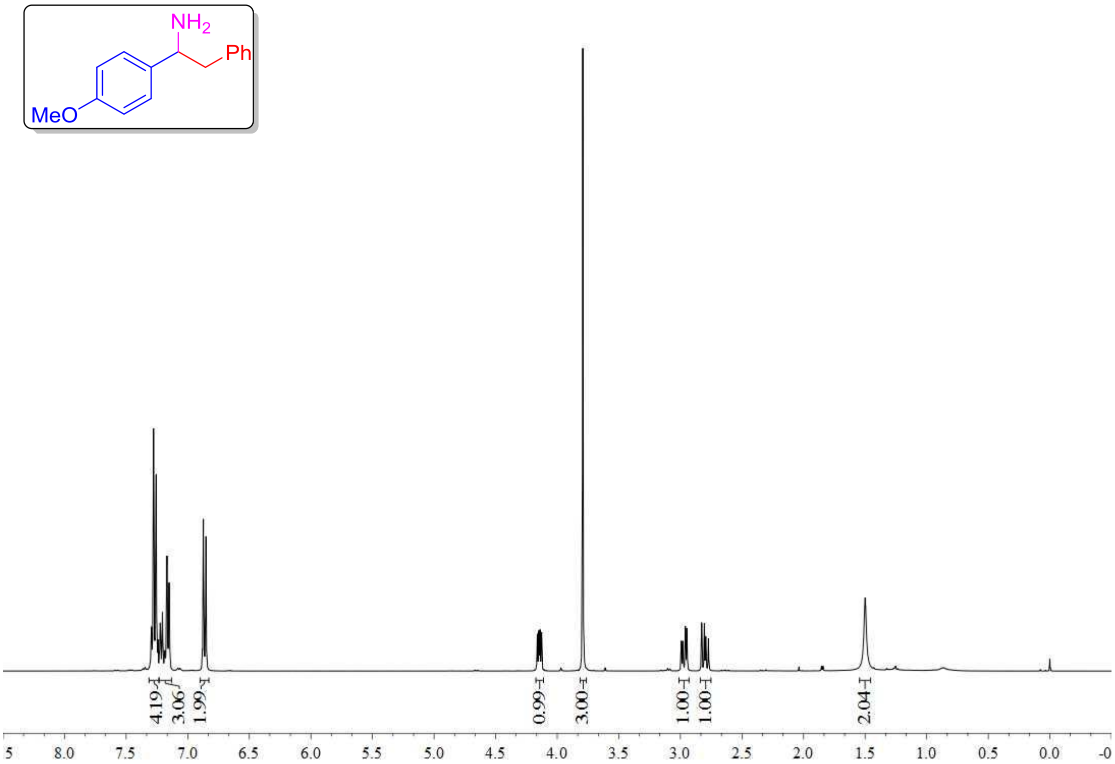

Supplementary Figure 8. **^{13}C NMR Spectrum of 3da (101 MHz, $CDCl_3$)**

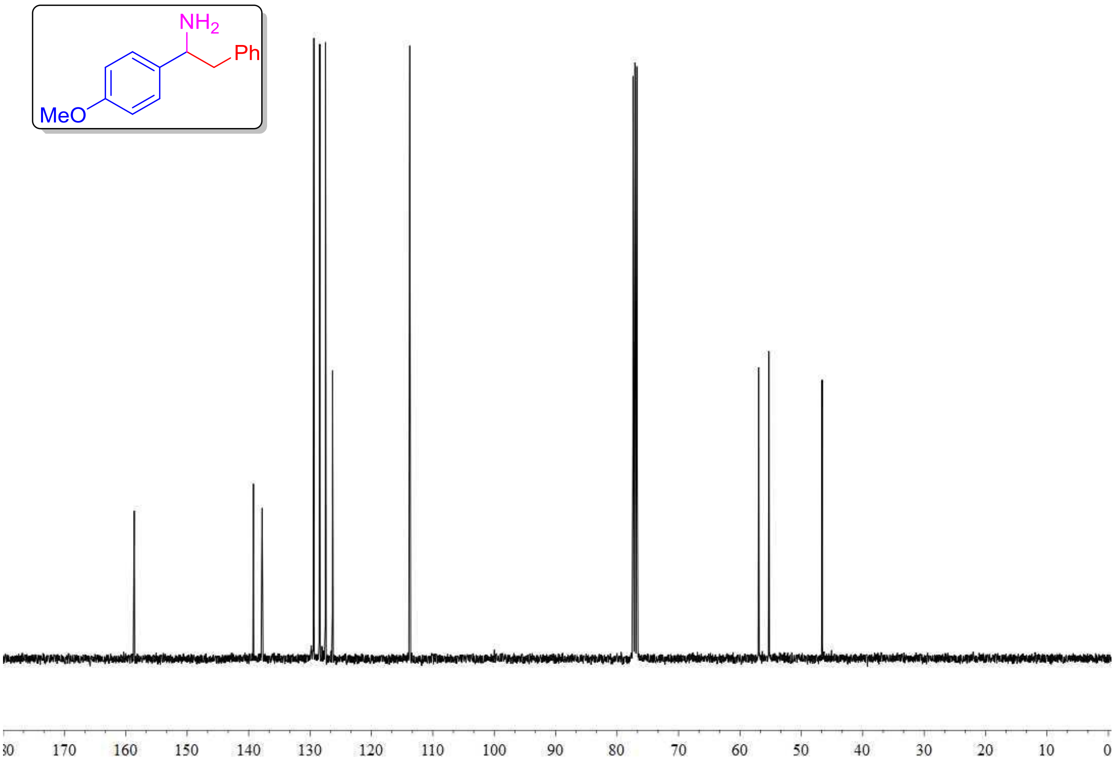

Supplementary Figure 9. ^{1}**H NMR Spectrum of 3ea (400 MHz,** $CDCl_3$**)**

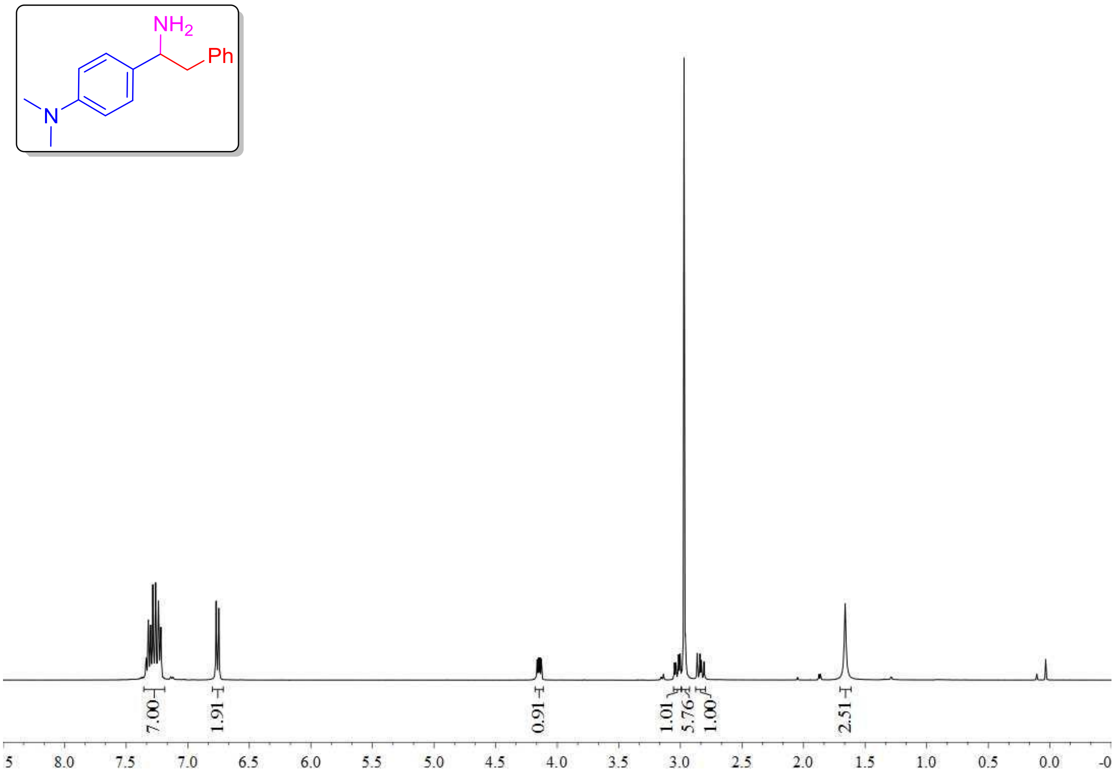

Supplementary Figure 10. ^{13}**C NMR Spectrum of 3ea (101 MHz,** $CDCl_3$**)**

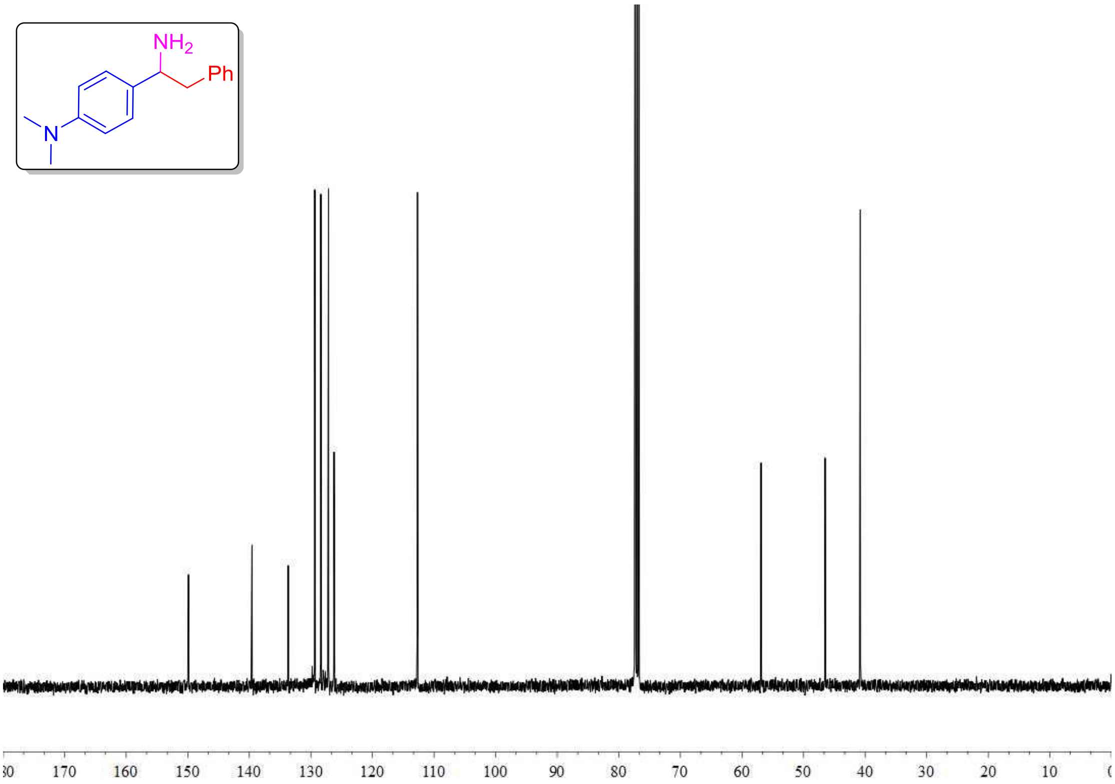

Supplementary Figure 11. ^{1}**H NMR Spectrum of 3fa (400 MHz, CDCl$_3$)**

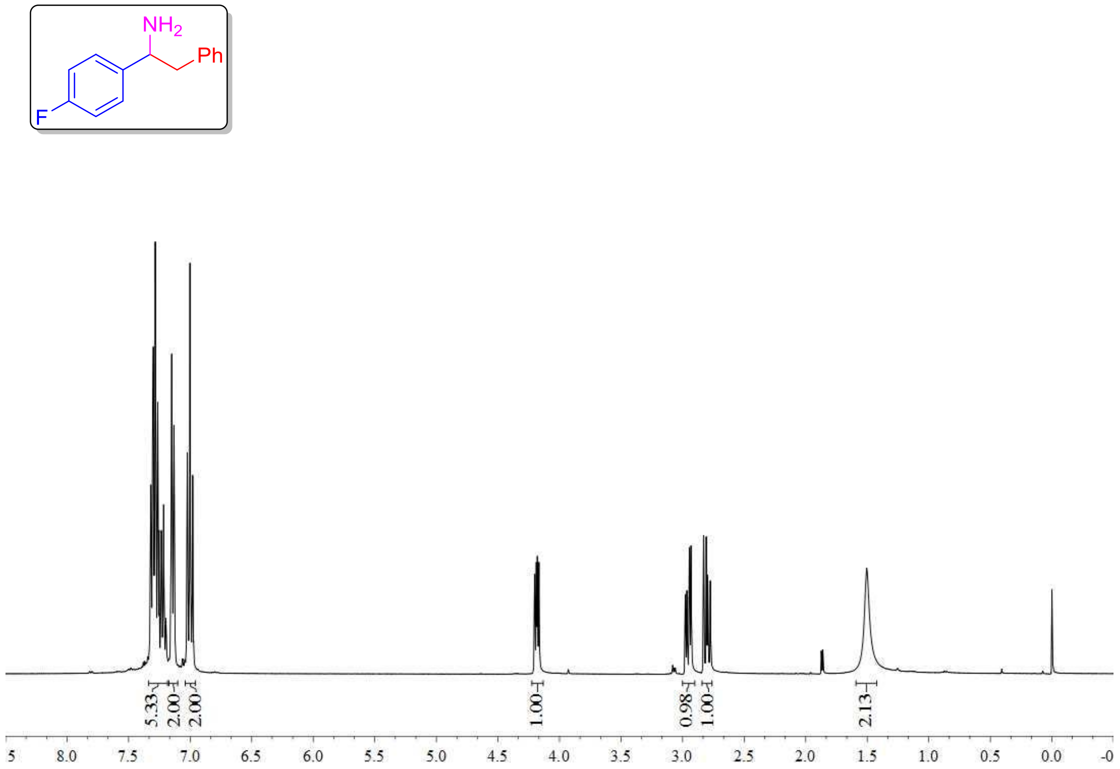

Supplementary Figure 12. ^{13}**C NMR Spectrum of 3fa (101 MHz, CDCl$_3$)**

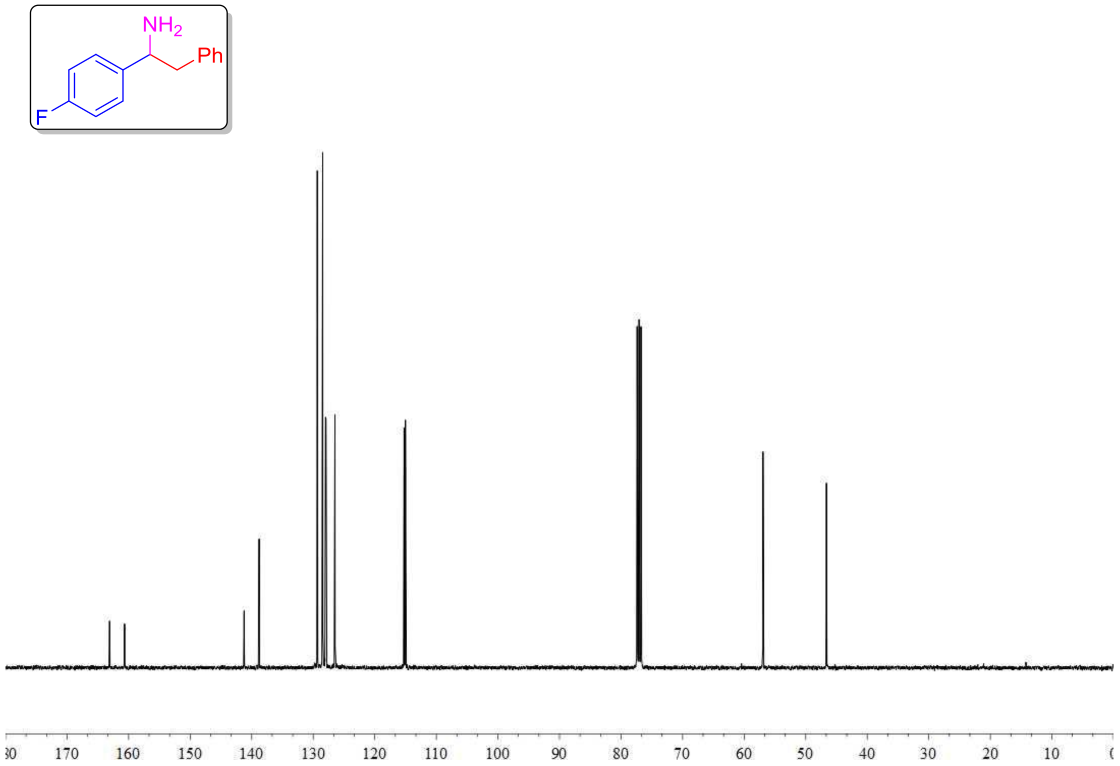

Supplementary Figure 13. ^{1}H NMR Spectrum of 3ga (400 MHz, $CDCl_3$)

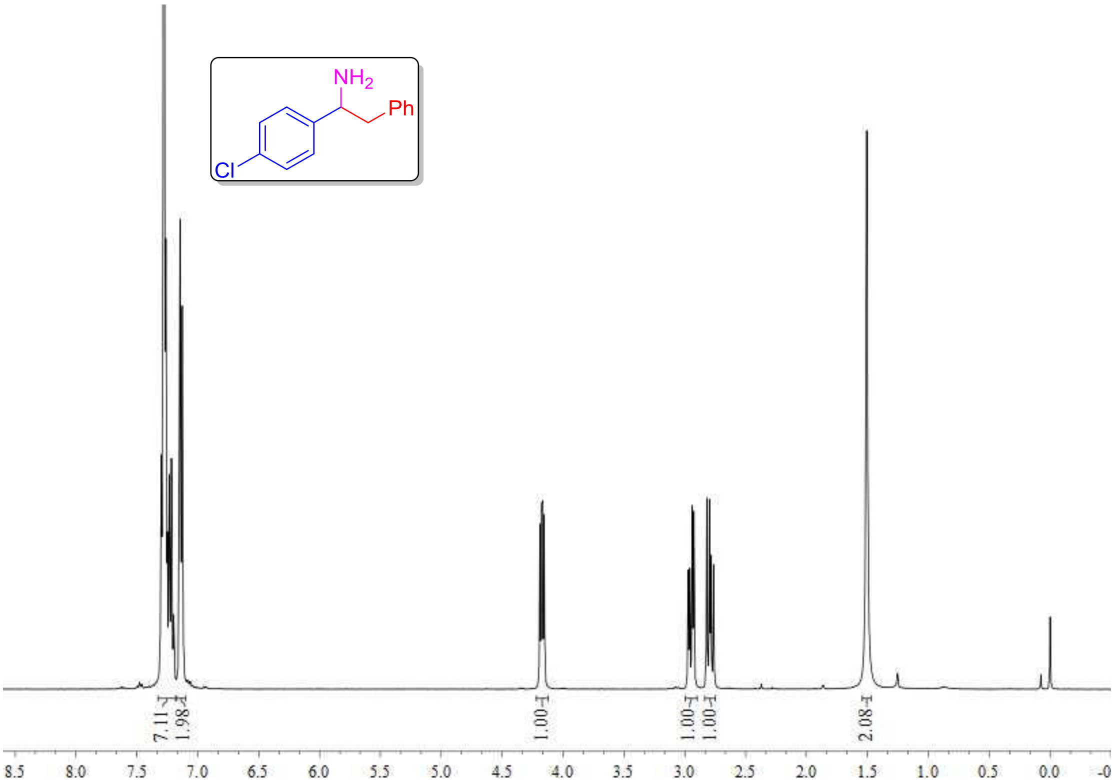

Supplementary Figure 14. ^{13}C NMR Spectrum of 3ga (101 MHz, $CDCl_3$)

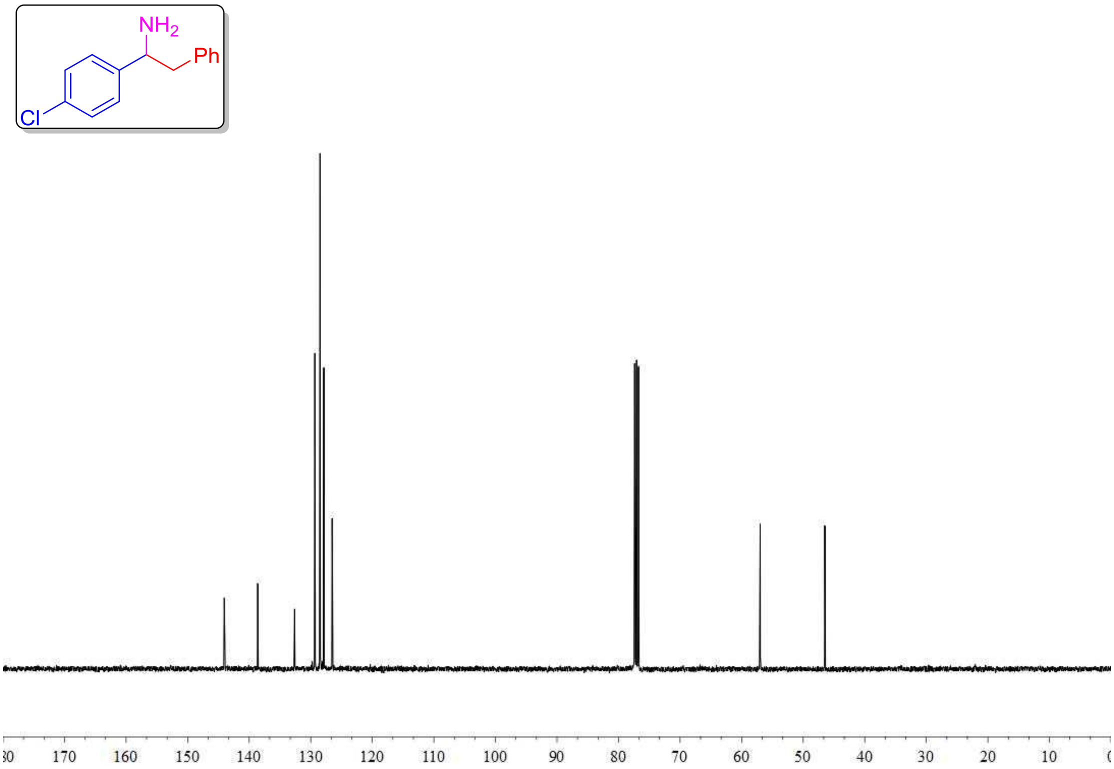

Supplementary Figure 15. ^{1}H NMR Spectrum of 3ha (400 MHz, $CDCl_3$)

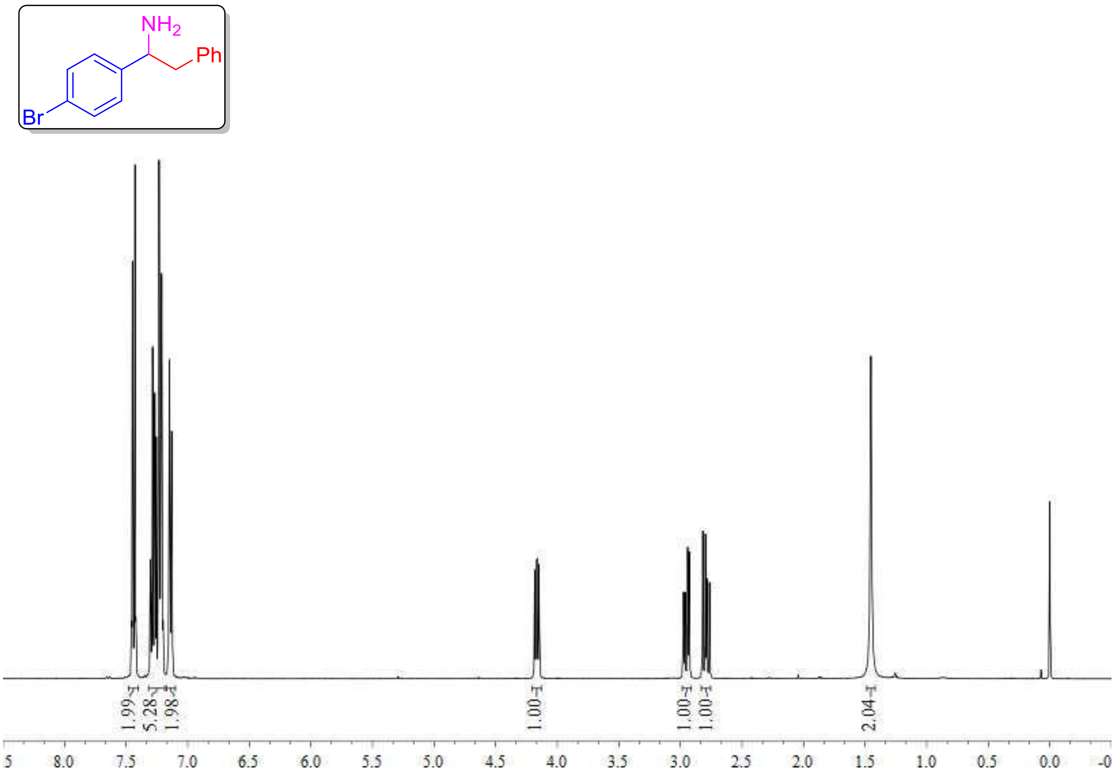

Supplementary Figure 16. ^{13}C NMR Spectrum of 3ha (101 MHz, $CDCl_3$)

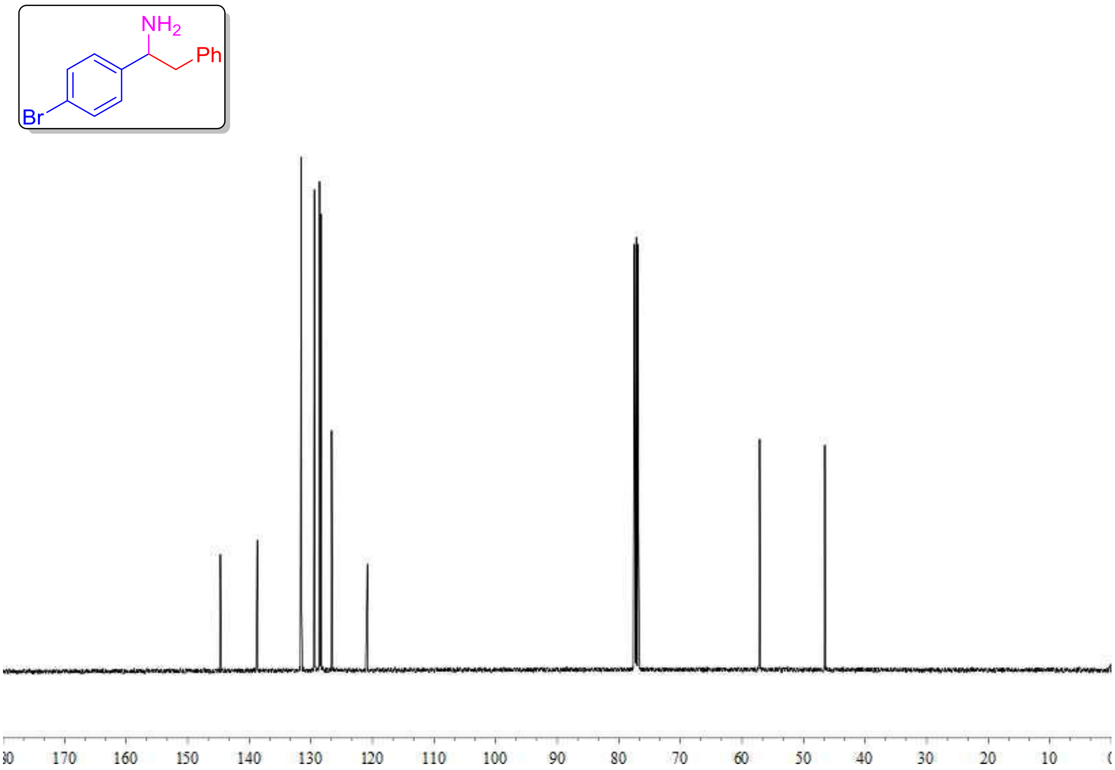

Supplementary Figure 17. ^{1}H NMR Spectrum of 3ia (400 MHz, $CDCl_3$)

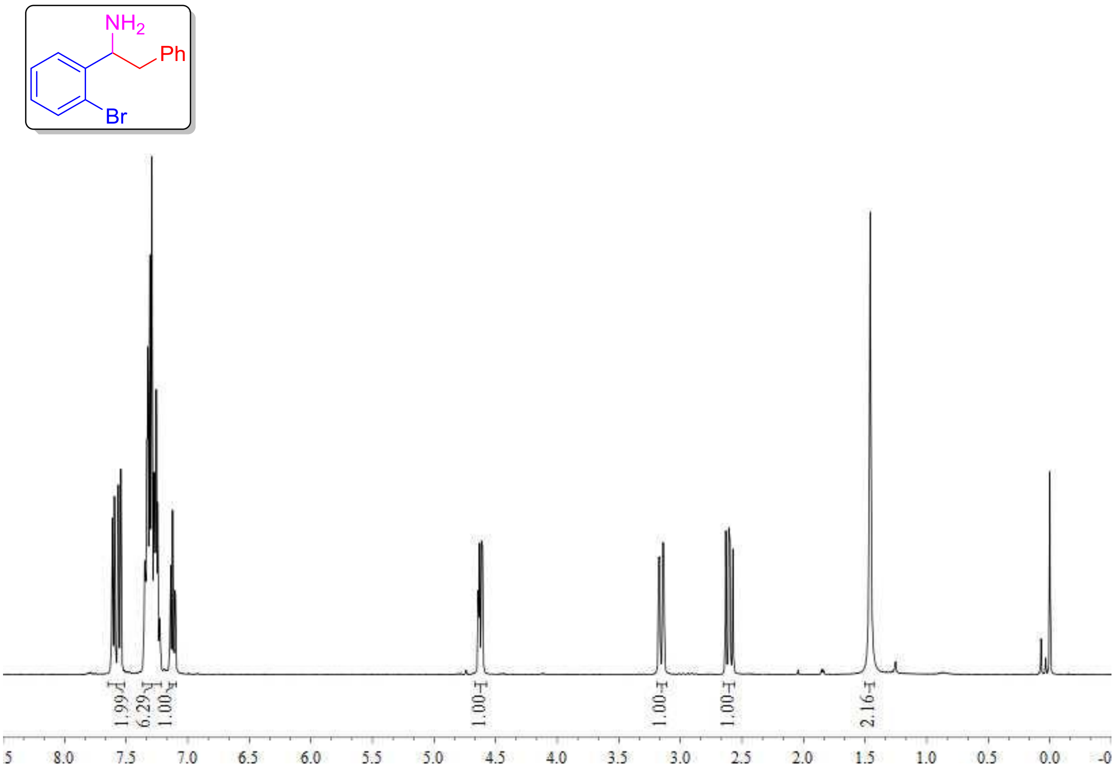

Supplementary Figure 18. ^{13}C NMR Spectrum of 3ia (101 MHz, $CDCl_3$)

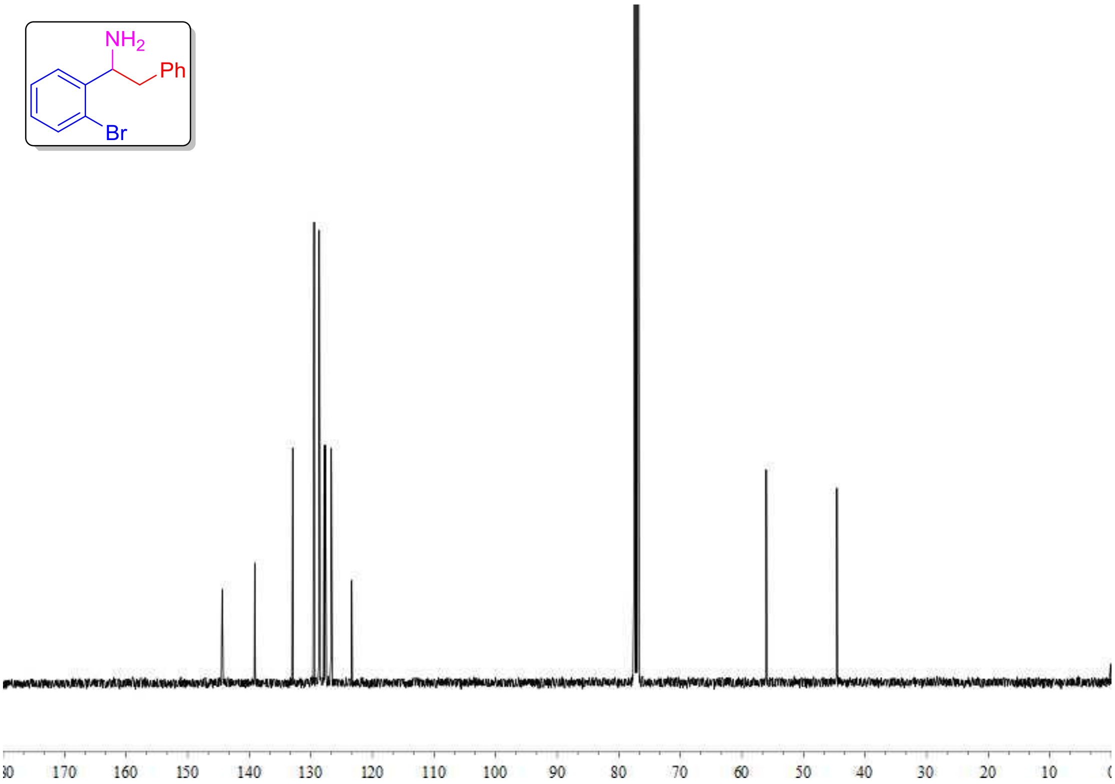

Supplementary Figure 19. ^{1}H NMR Spectrum of 3ja (400 MHz, $CDCl_3$)

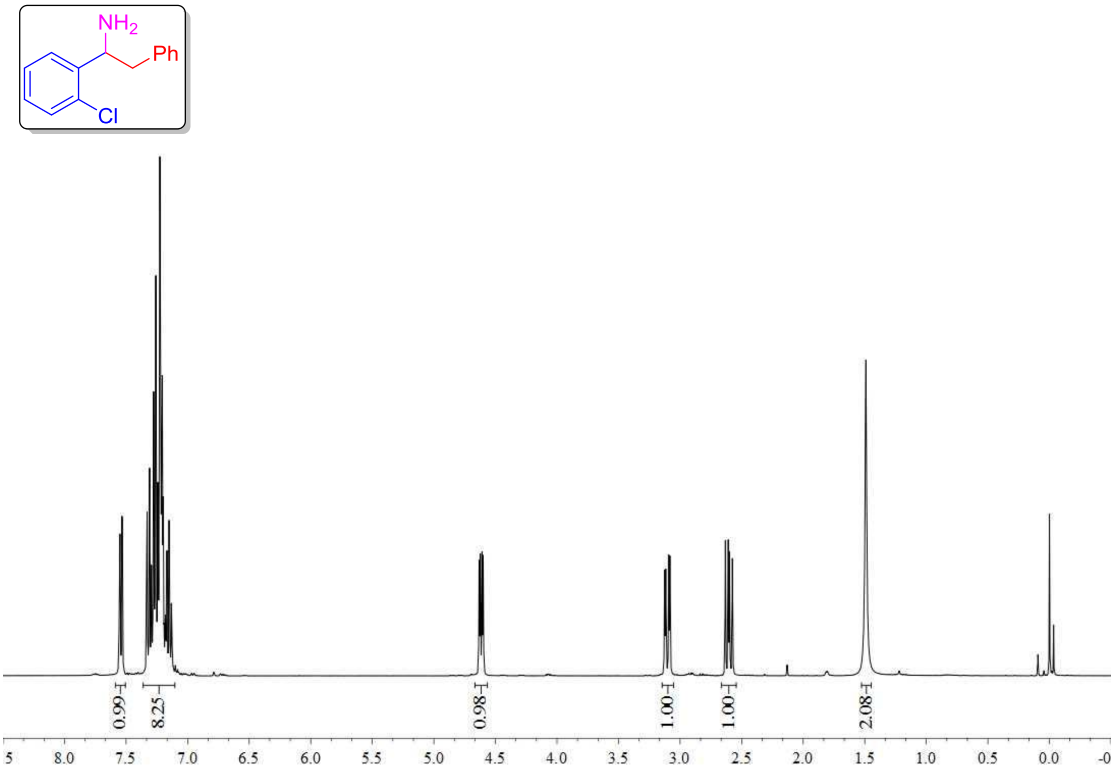

Supplementary Figure 20. ^{13}C NMR Spectrum of 3ja (101 MHz, $CDCl_3$)

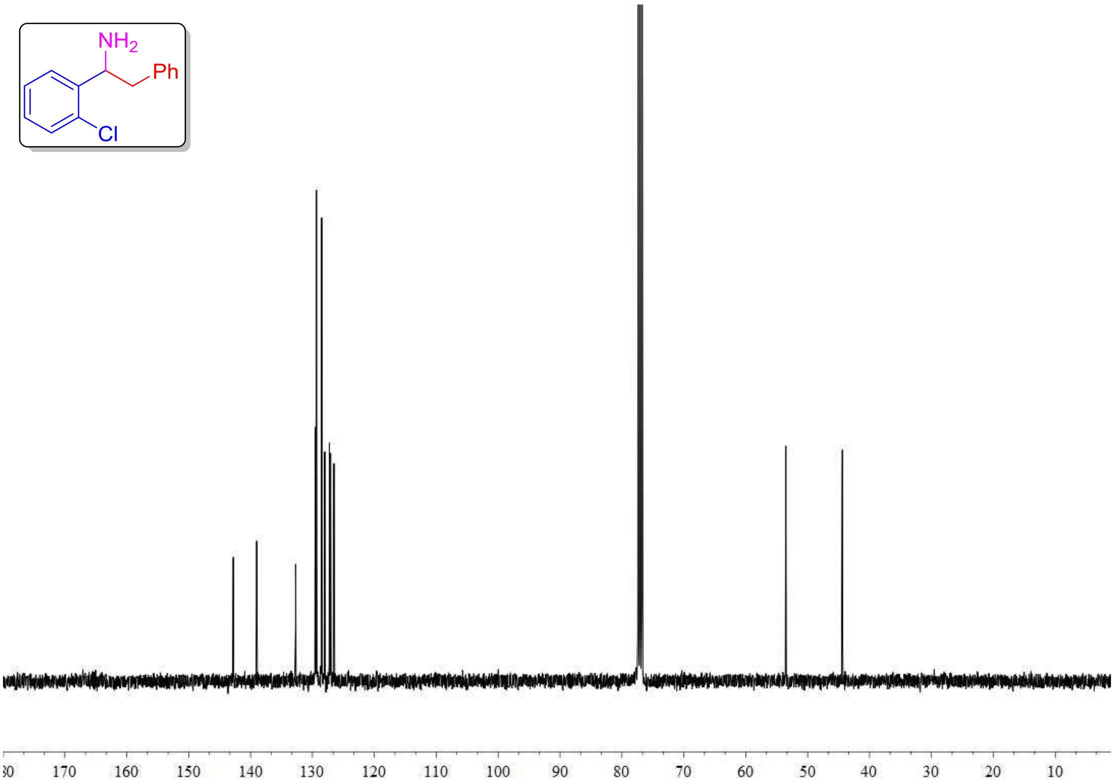

Supplementary Figure 21. ^{1}**H NMR Spectrum of 3ka (400 MHz, $CDCl_3$)**

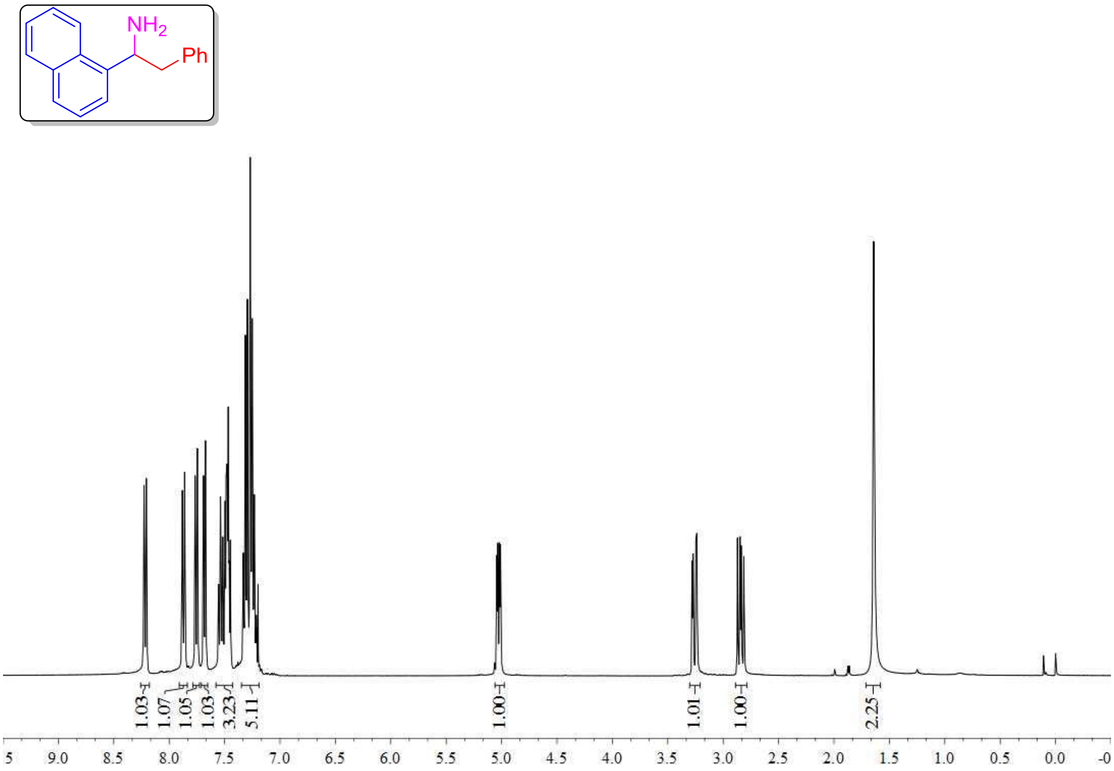

Supplementary Figure 22. ^{13}**C NMR Spectrum of 3ka (101 MHz, $CDCl_3$)**

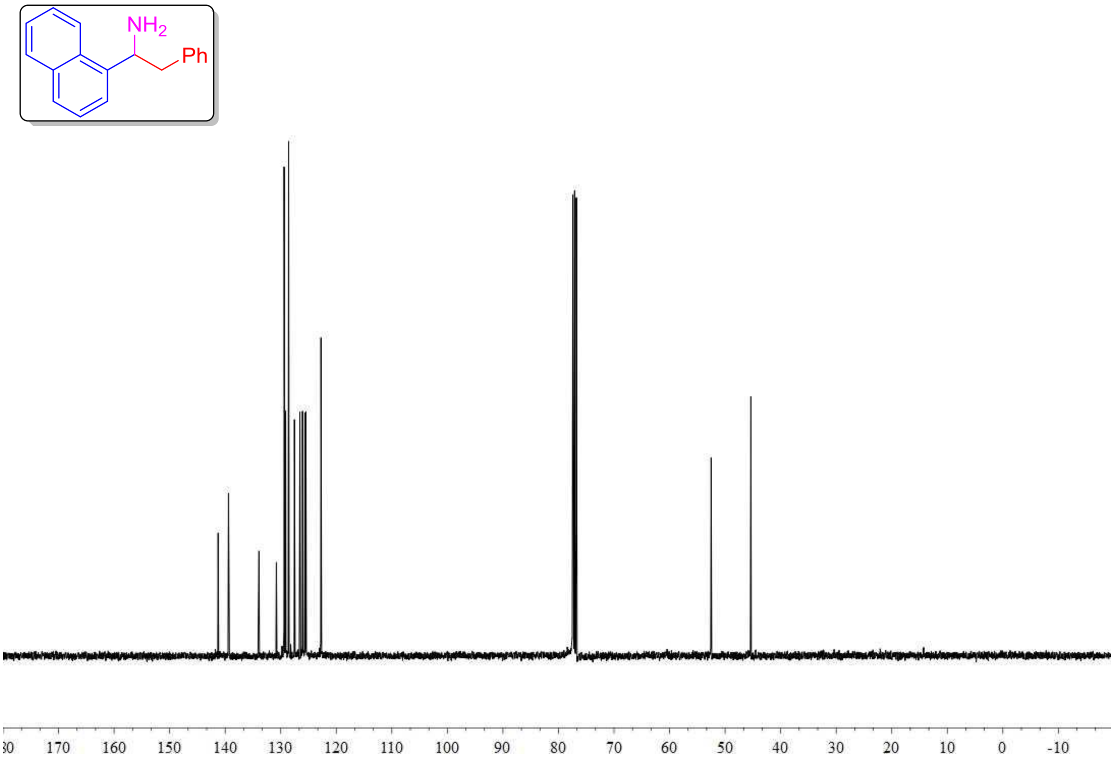

Supplementary Figure 23. ^{1}H **NMR Spectrum of 3la (400 MHz,** $CDCl_3$**)**

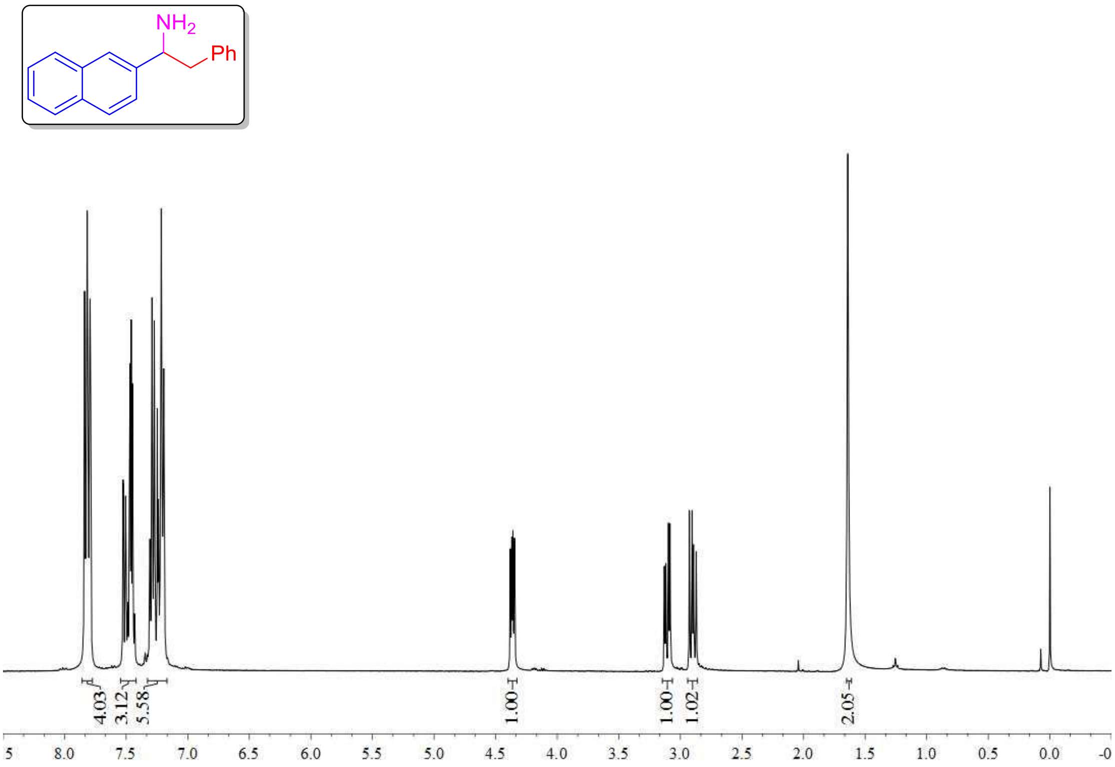

Supplementary Figure 24. ^{13}C **NMR Spectrum of 3la (101 MHz,** $CDCl_3$**)**

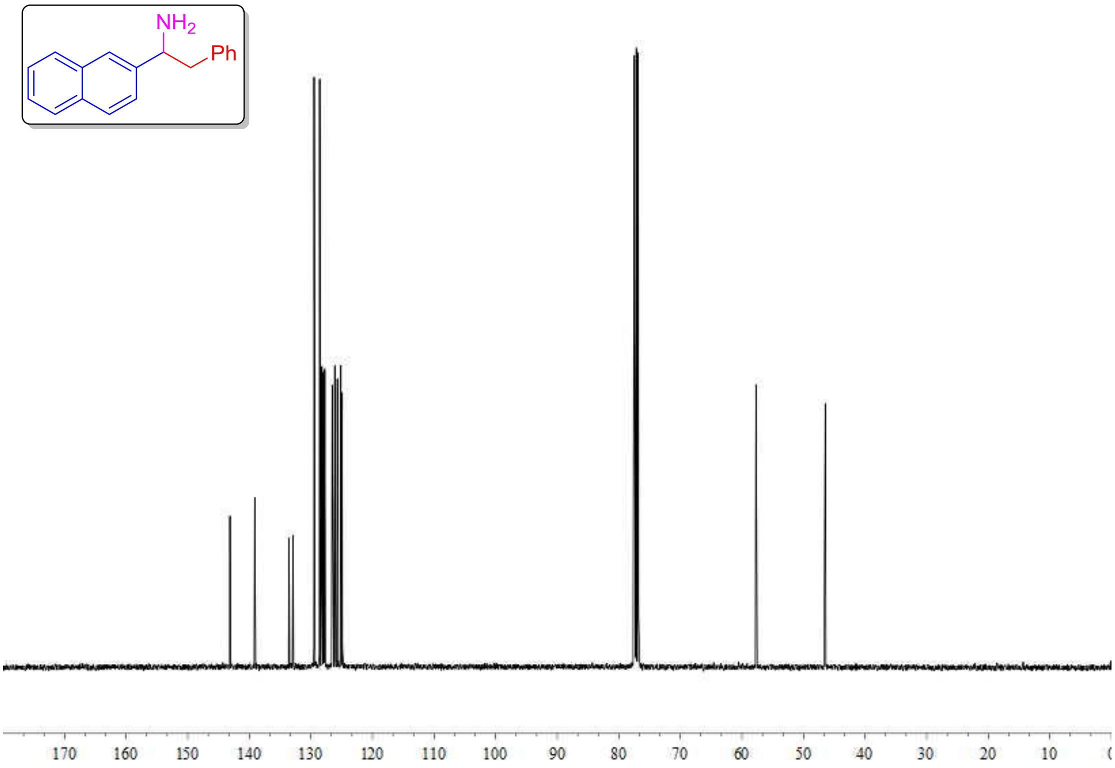

Supplementary Figure 25. ^{1}H NMR Spectrum of 3ma (400 MHz, $CDCl_3$)

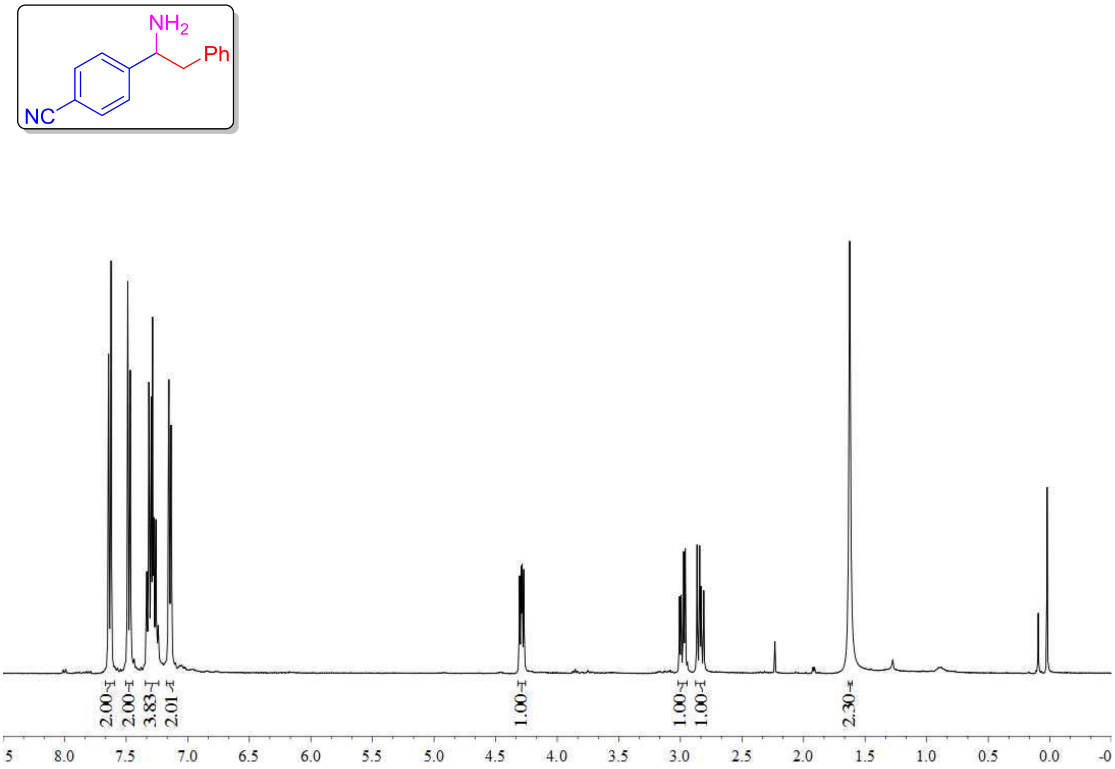

Supplementary Figure 26. ^{13}C NMR Spectrum of 3ma (101 MHz, $CDCl_3$)

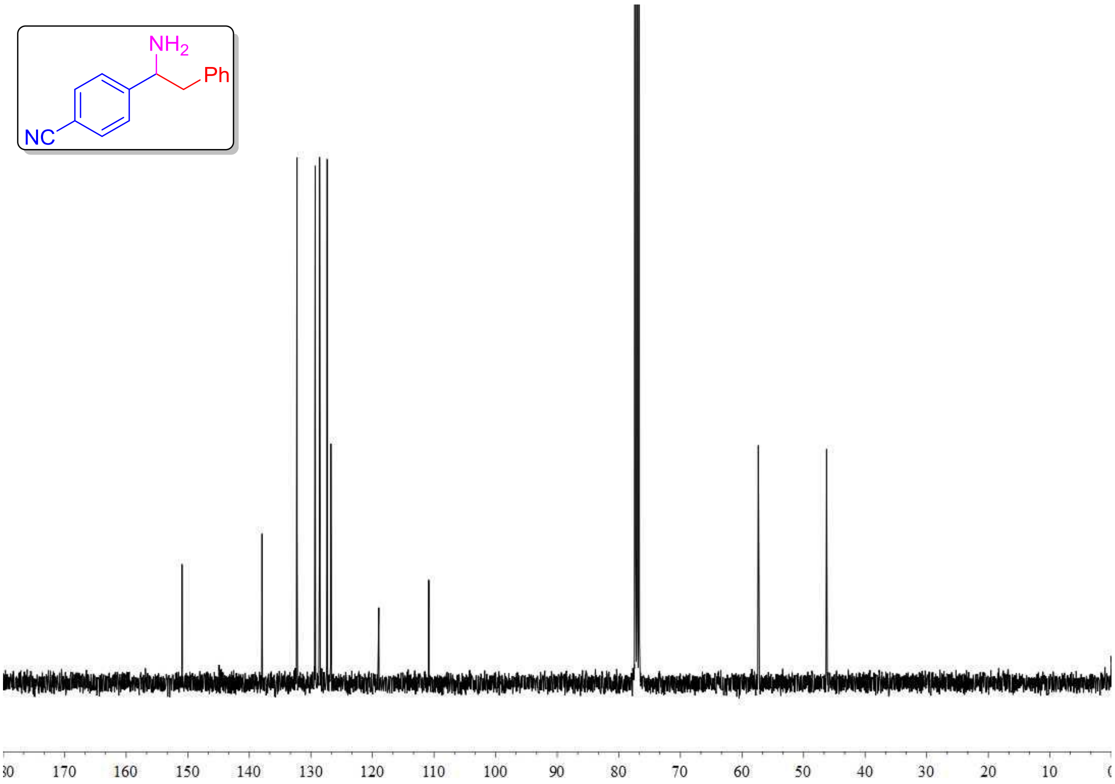

Supplementary Figure 27. ^{1}H NMR Spectrum of 3na (400 MHz, $CDCl_3$)

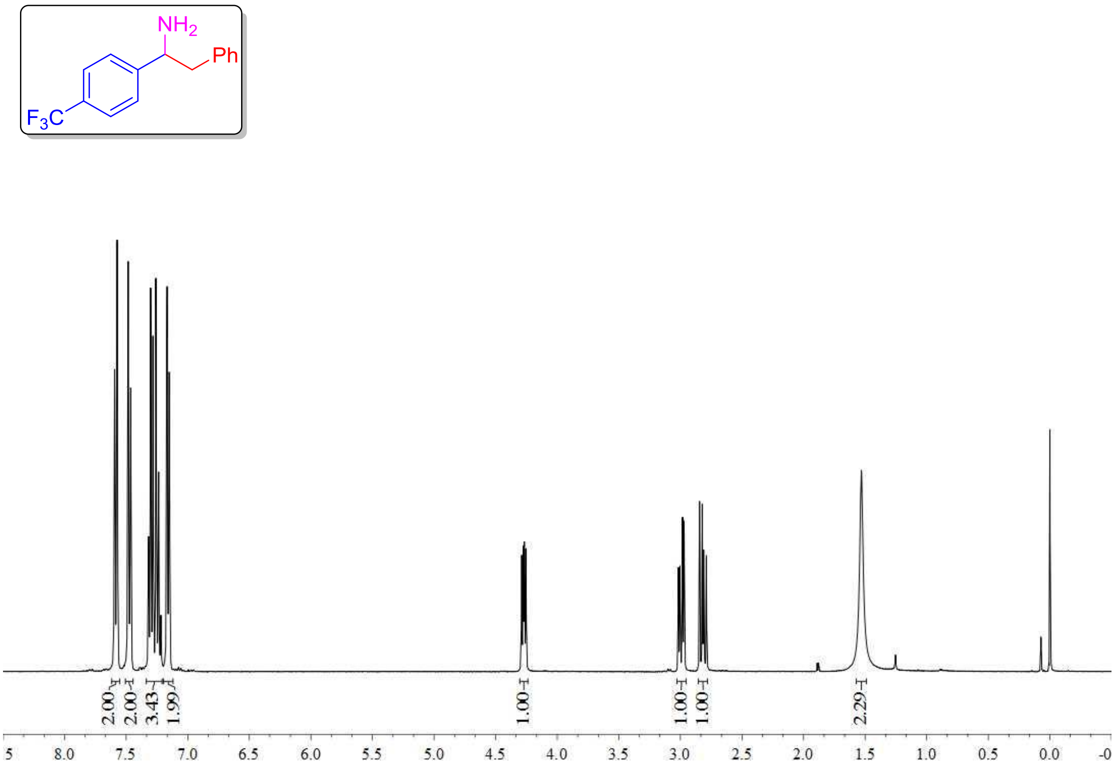

Supplementary Figure 28. ^{13}C NMR Spectrum of 3na (101 MHz, $CDCl_3$)

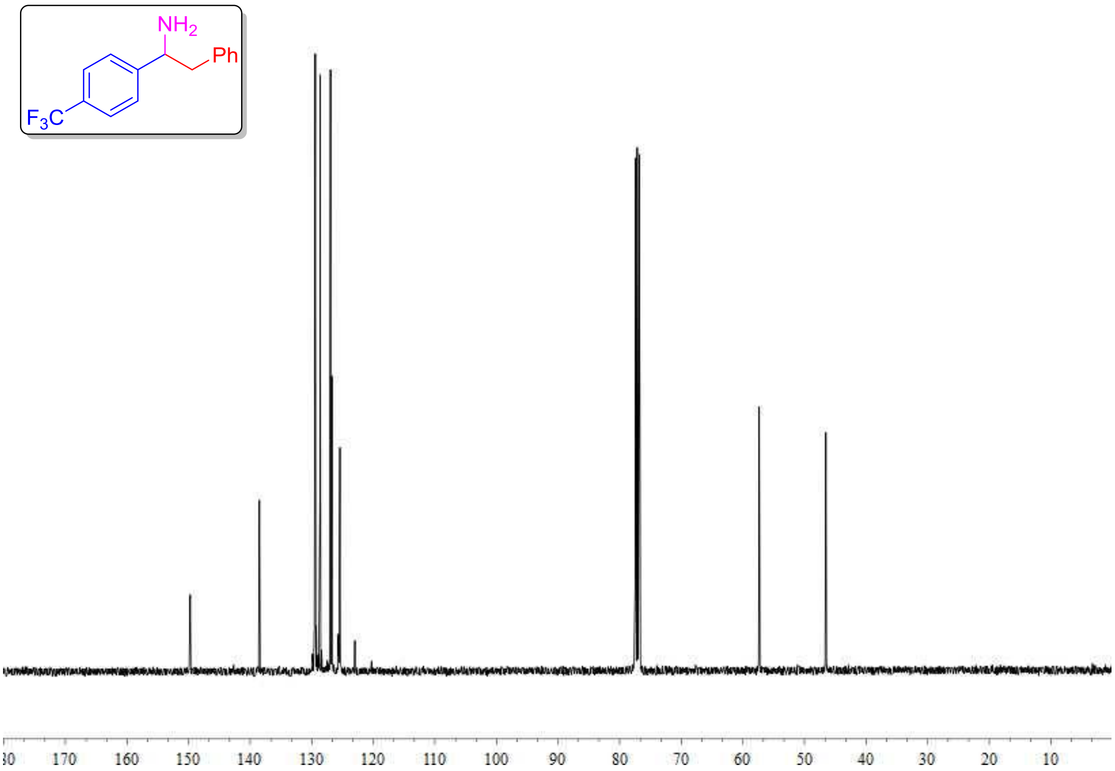

Supplementary Figure 29. **^{1}H NMR Spectrum of 3oa (400 MHz, $CDCl_3$)**

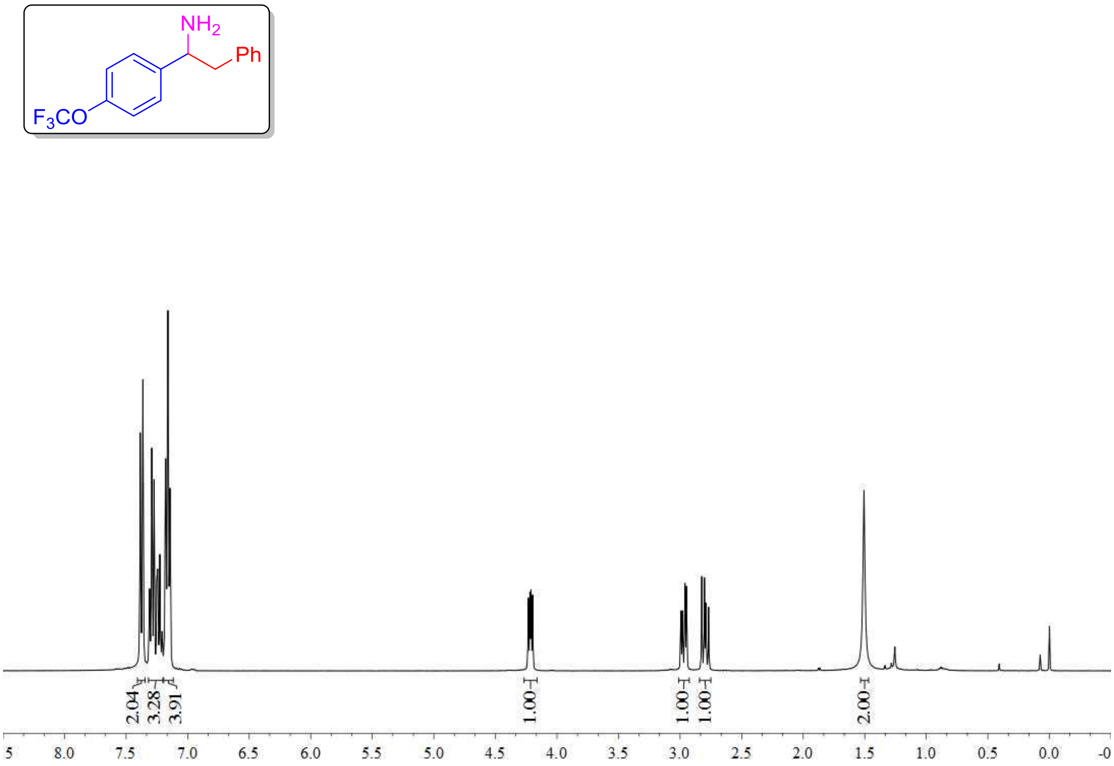

Supplementary Figure 30. **^{13}C NMR Spectrum of 3oa (101 MHz, $CDCl_3$)**

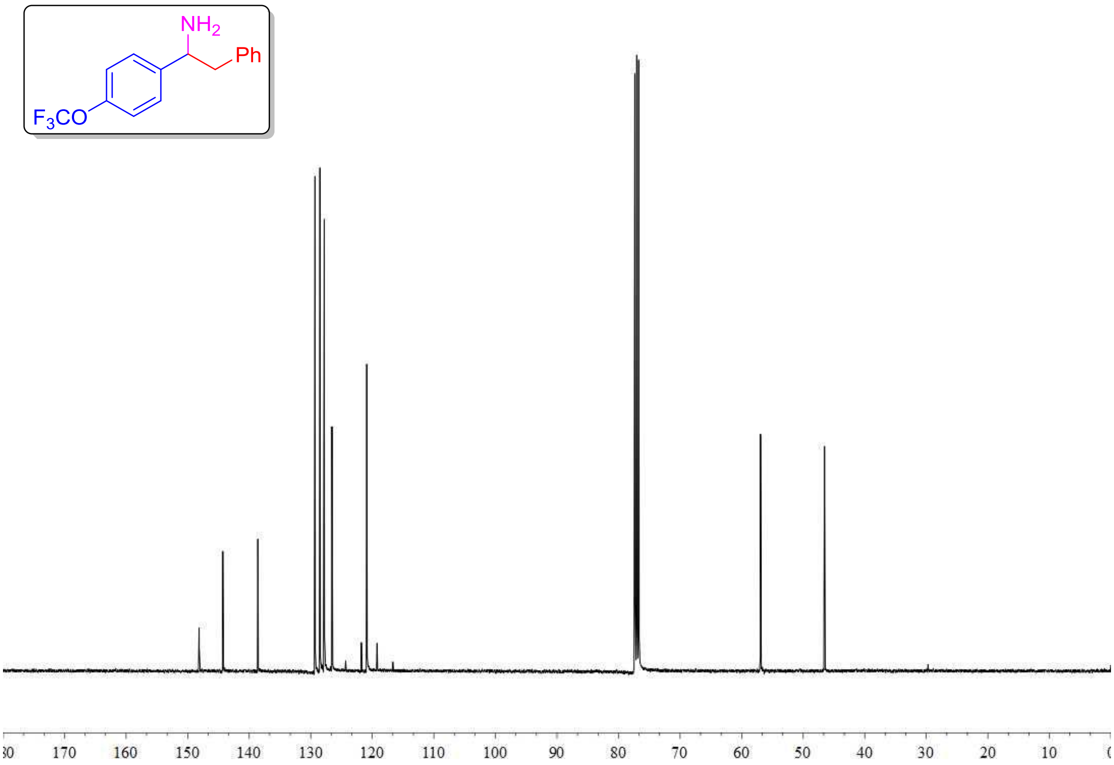

Supplementary Figure 31. ^{1}**H NMR Spectrum of 3pa (400 MHz,** $CDCl_3$**)**

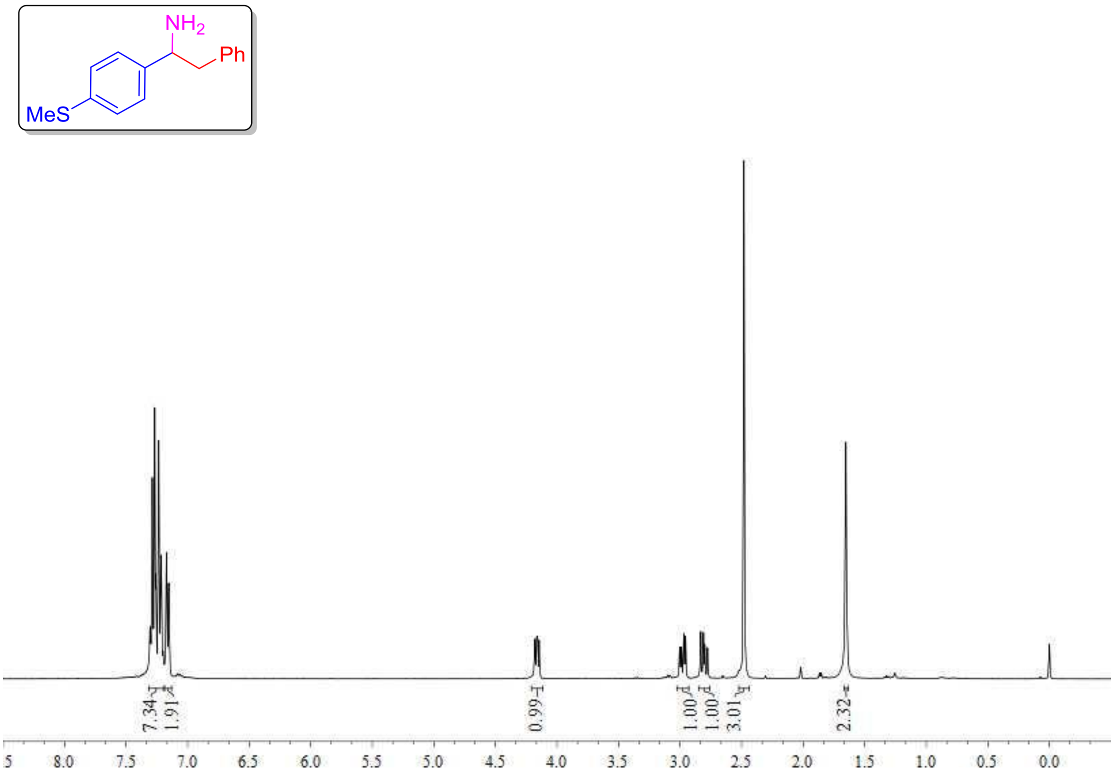

Supplementary Figure 32. ^{13}**C NMR Spectrum of 3pa (101 MHz,** $CDCl_3$**)**

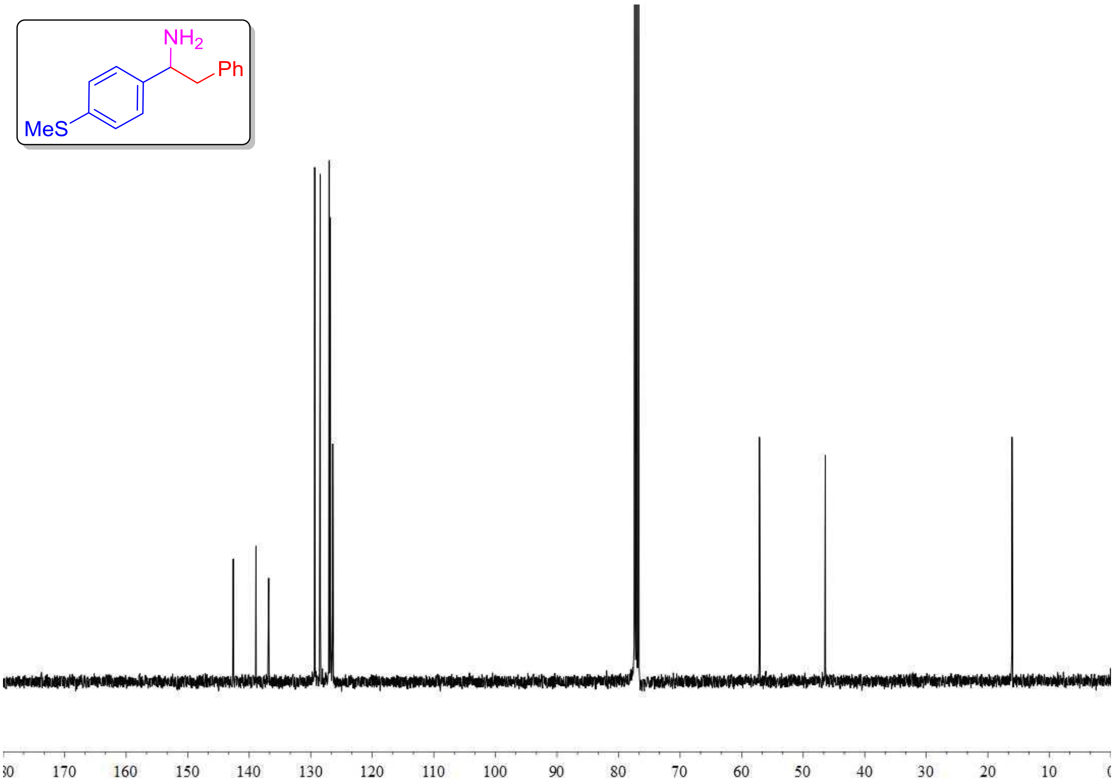

Supplementary Figure 33. ^{1}H NMR Spectrum of 3qa (400 MHz, $CDCl_3$)

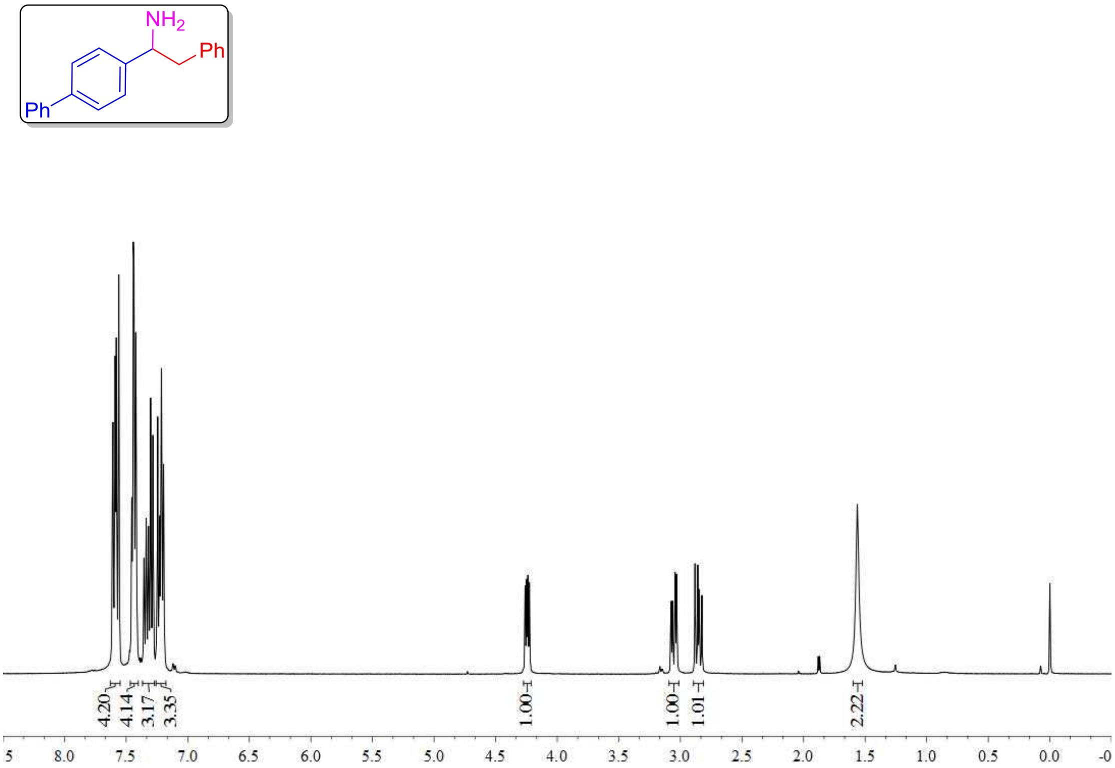

Supplementary Figure 34. ^{13}C NMR Spectrum of 3qa (101 MHz, $CDCl_3$)

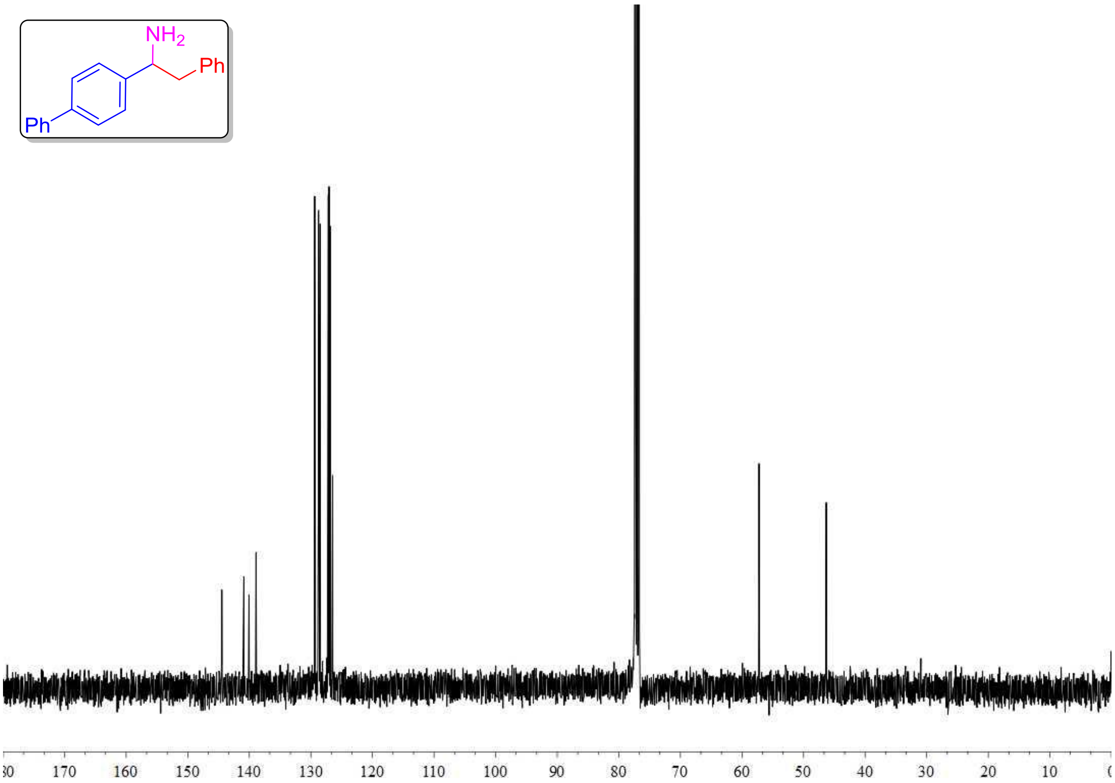

Supplementary Figure 35. ^{1}H NMR Spectrum of 3ra (400 MHz, CDCl$_3$)

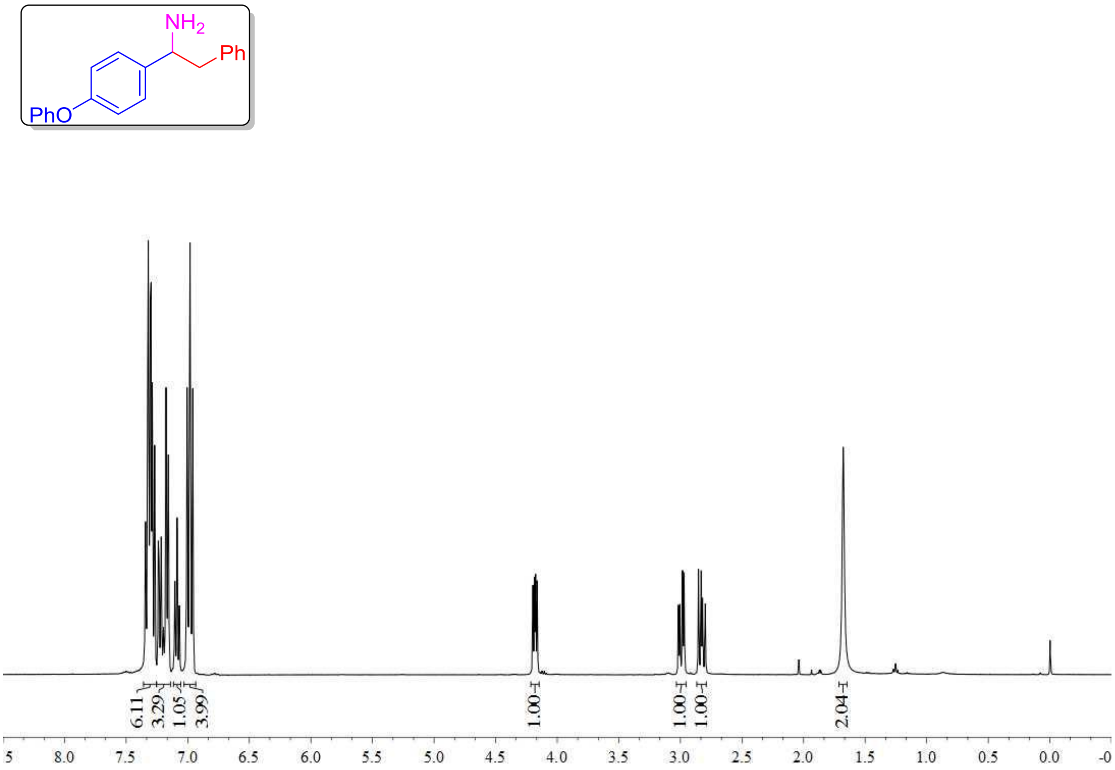

Supplementary Figure 36. ^{13}C NMR Spectrum of 3ra (101 MHz, CDCl$_3$)

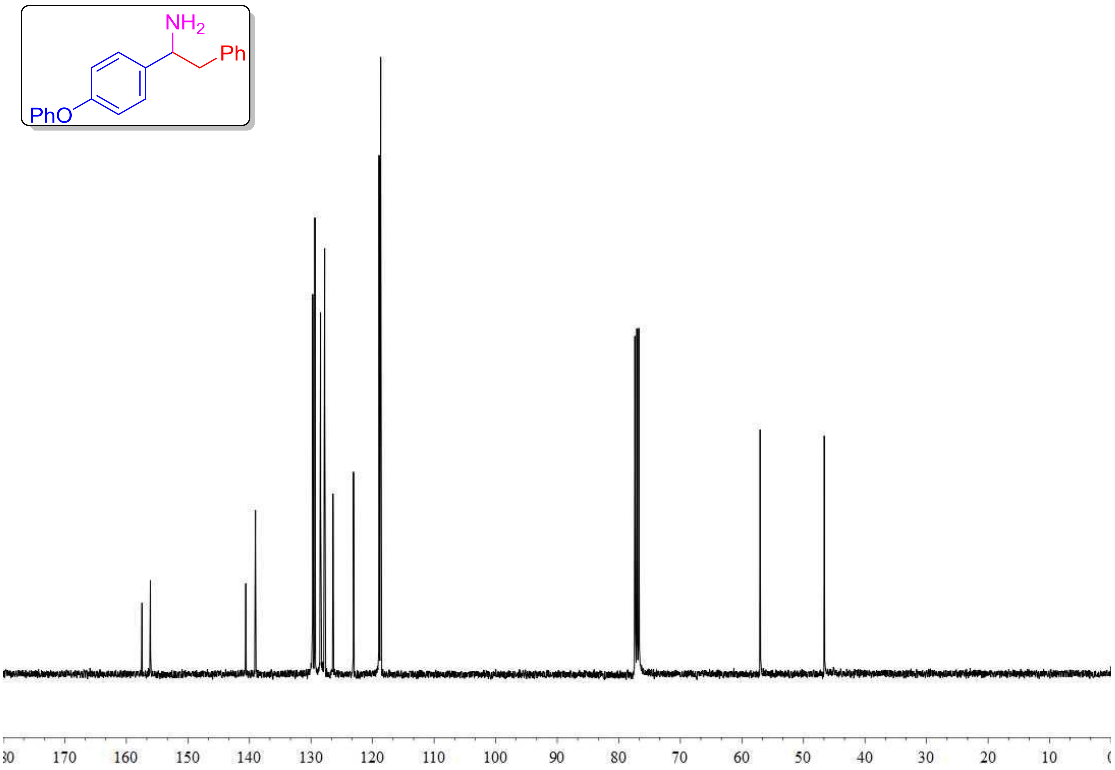

Supplementary Figure 37. ^{1}H NMR Spectrum of 3sa (400 MHz, $CDCl_3$)

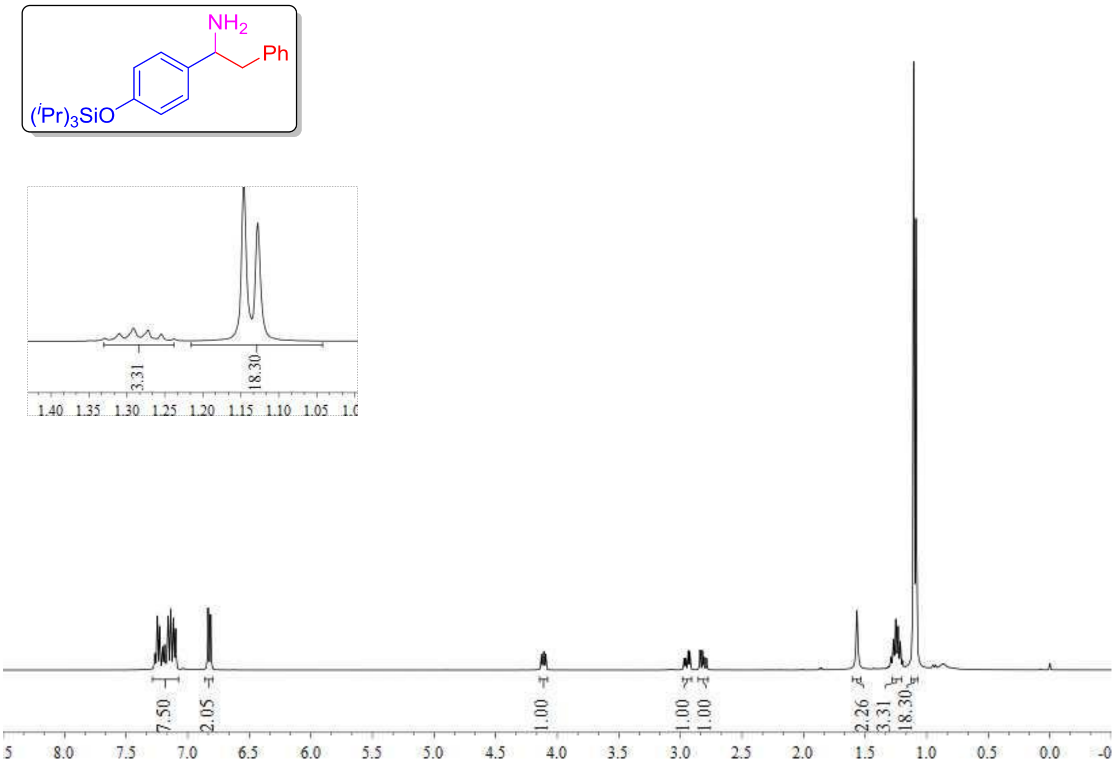

Supplementary Figure 38. ^{13}C NMR Spectrum of 3sa (101 MHz, $CDCl_3$)

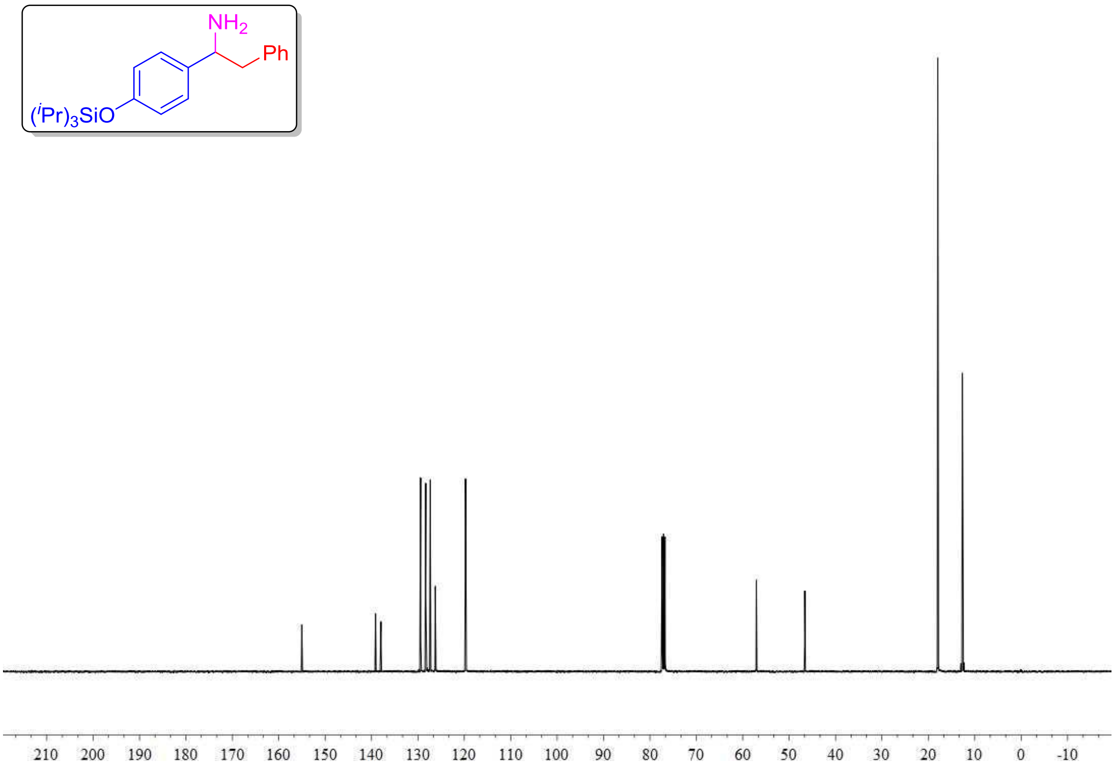

Supplementary Figure 39. ^{1}H NMR Spectrum of 3ta (400 MHz, $CDCl_3$)

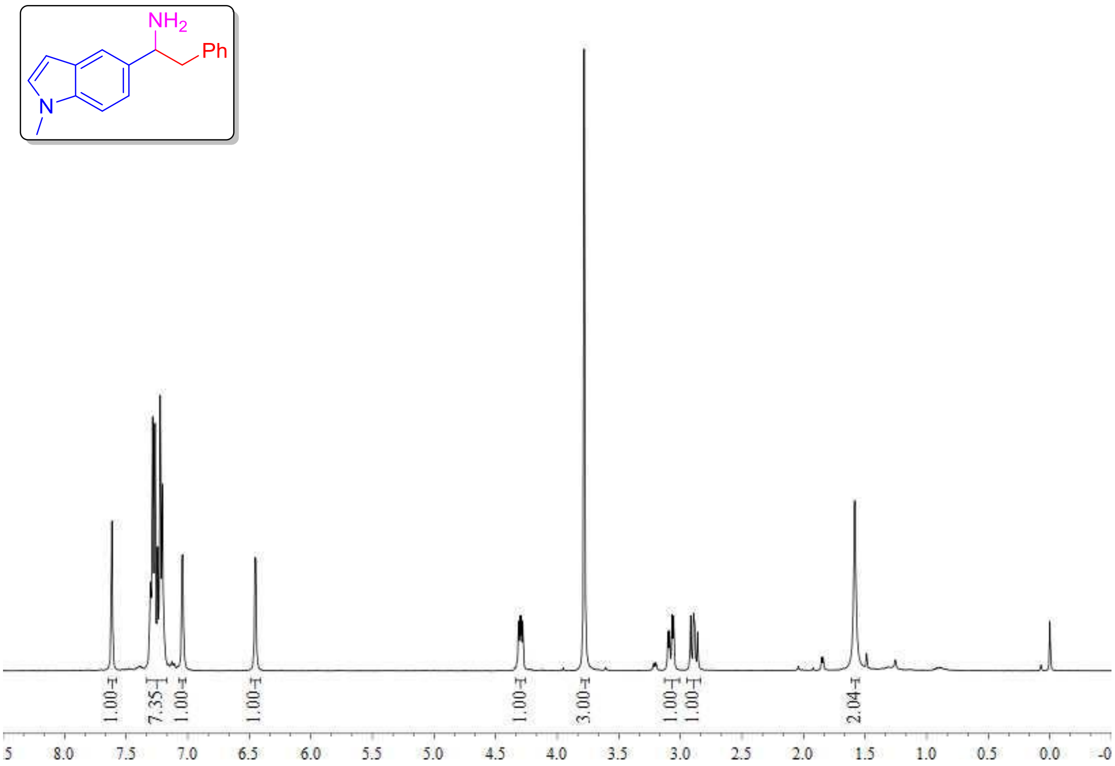

Supplementary Figure 40. ^{13}C NMR Spectrum of 3ta (101 MHz, $CDCl_3$)

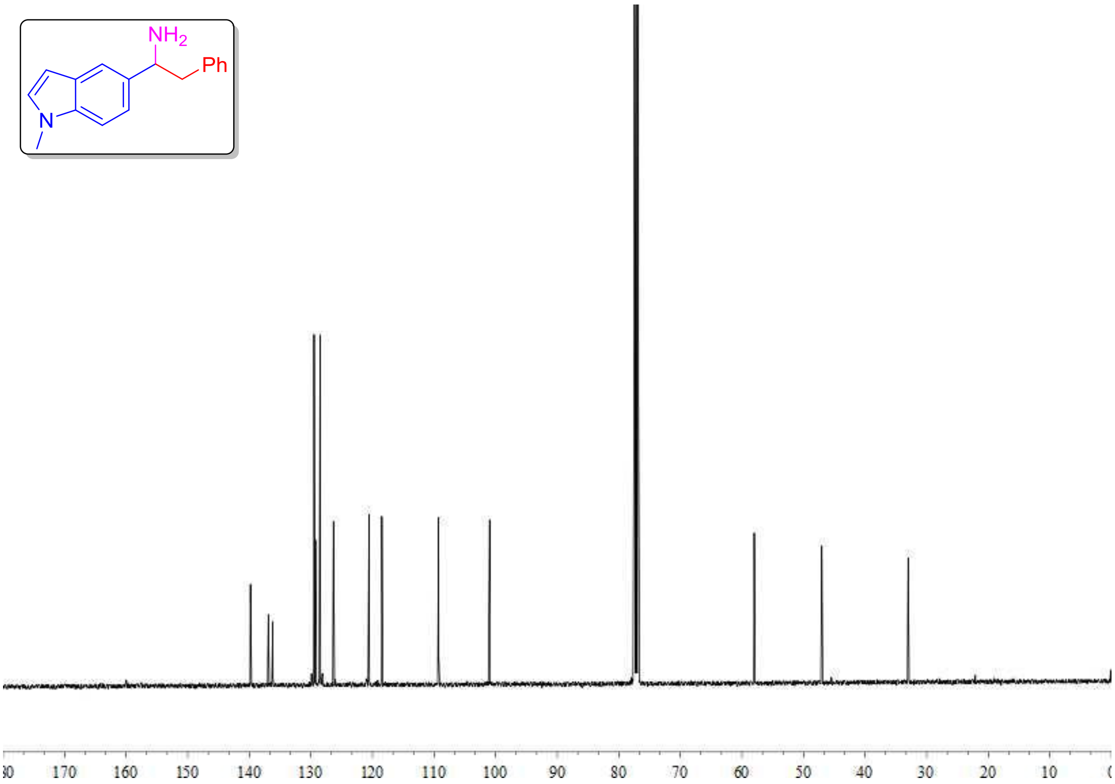

Supplementary Figure 41. ^{1}**H NMR Spectrum of 3ua (400 MHz,** $CDCl_3$**)**

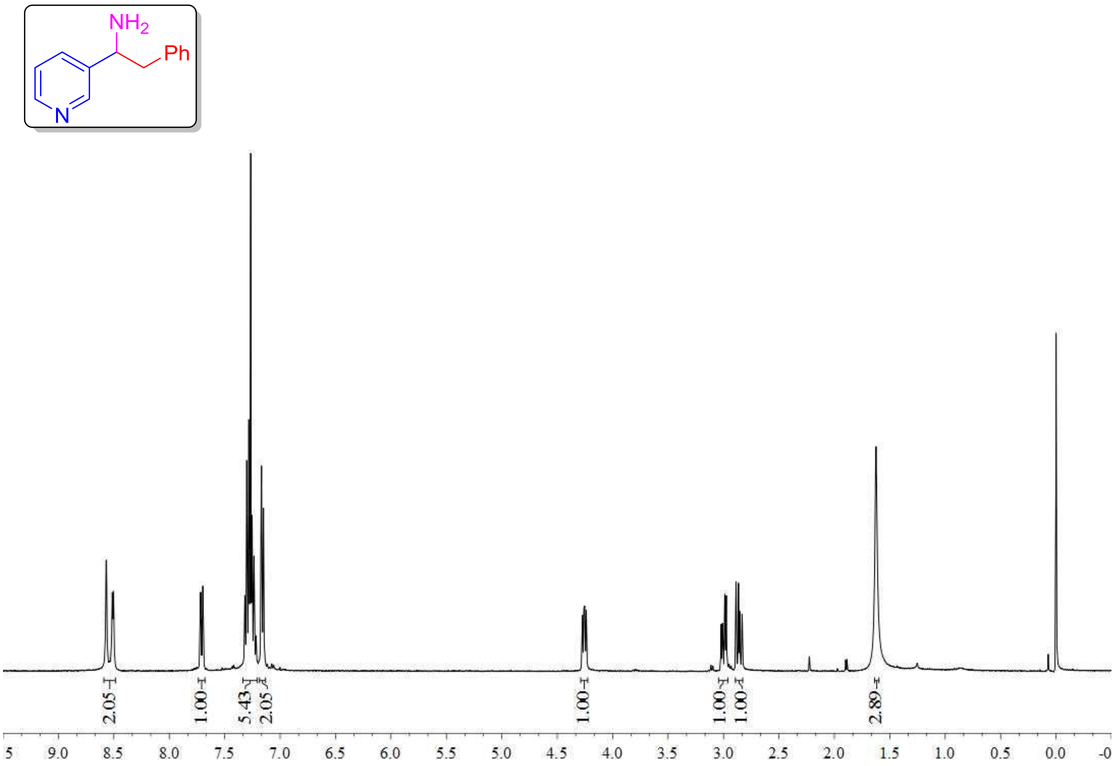

Supplementary Figure 42. ^{13}**C NMR Spectrum of 3ua (101 MHz,** $CDCl_3$**)**

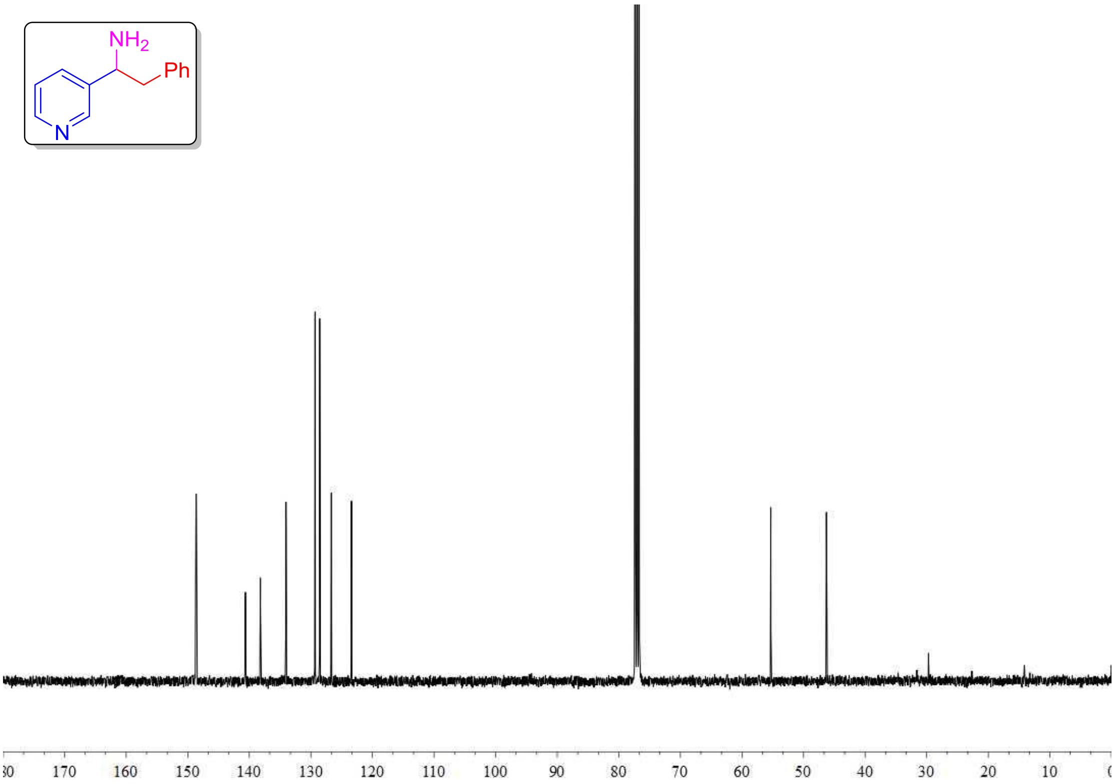

Supplementary Figure 43. ^{1}**H NMR Spectrum of 3va (400 MHz, CDCl$_3$)**

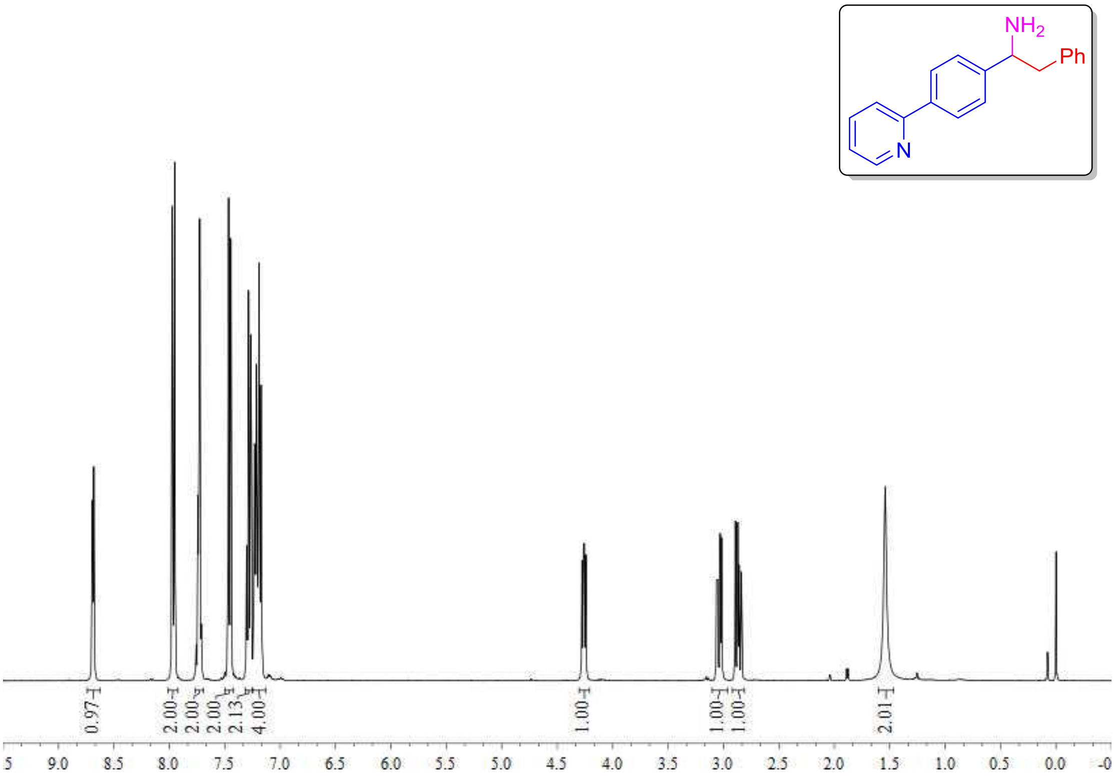

Supplementary Figure 44. ^{13}**C NMR Spectrum of 3va (101 MHz, CDCl$_3$)**

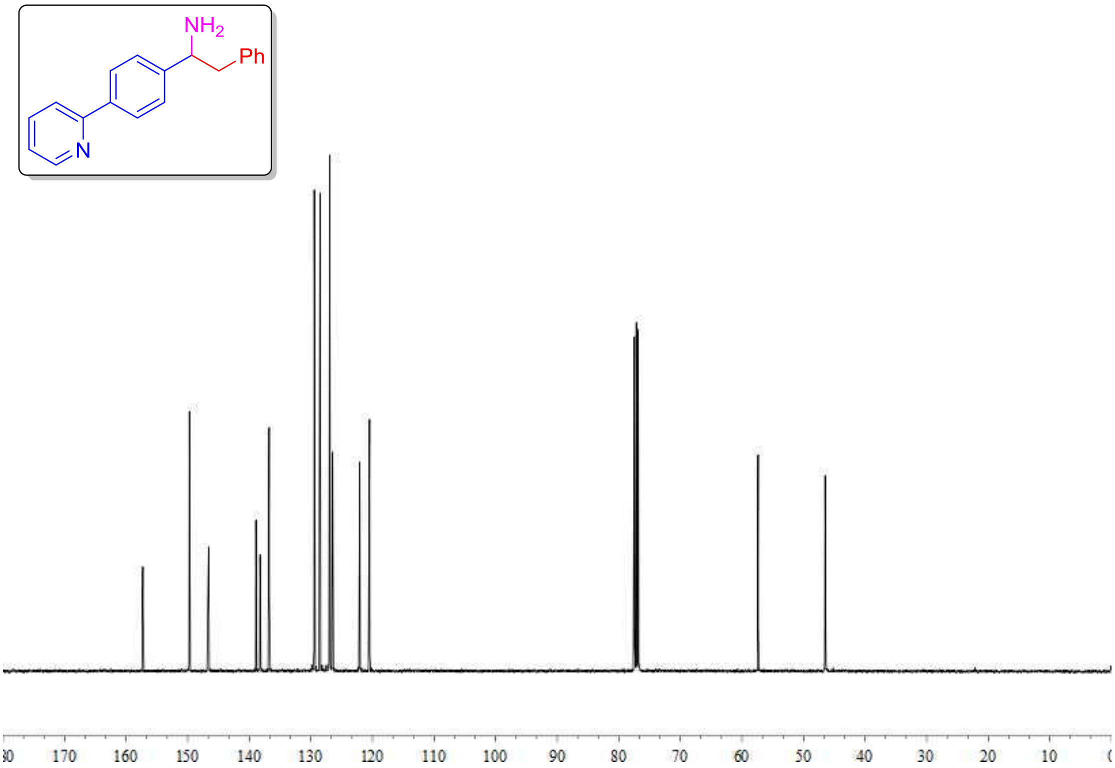

Supplementary Figure 45. ^{1}H NMR Spectrum of 3wa (400 MHz, $CDCl_3$)

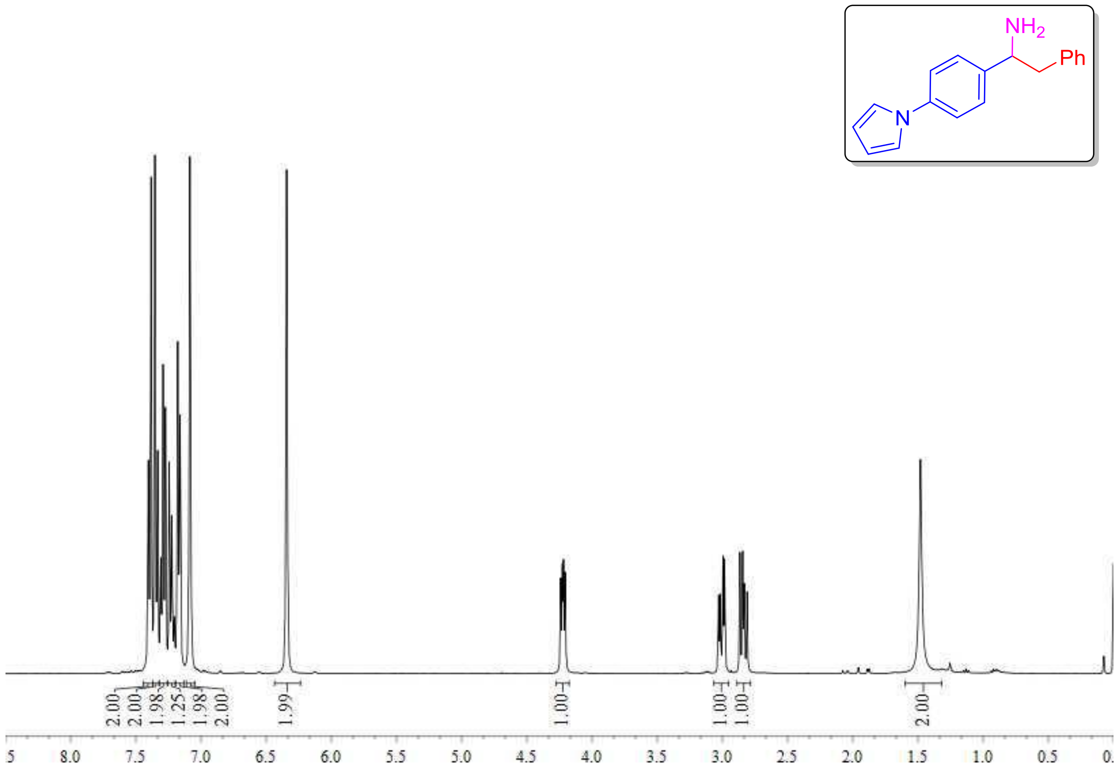

Supplementary Figure 46. ^{13}C NMR Spectrum of 3wa (101 MHz, $CDCl_3$)

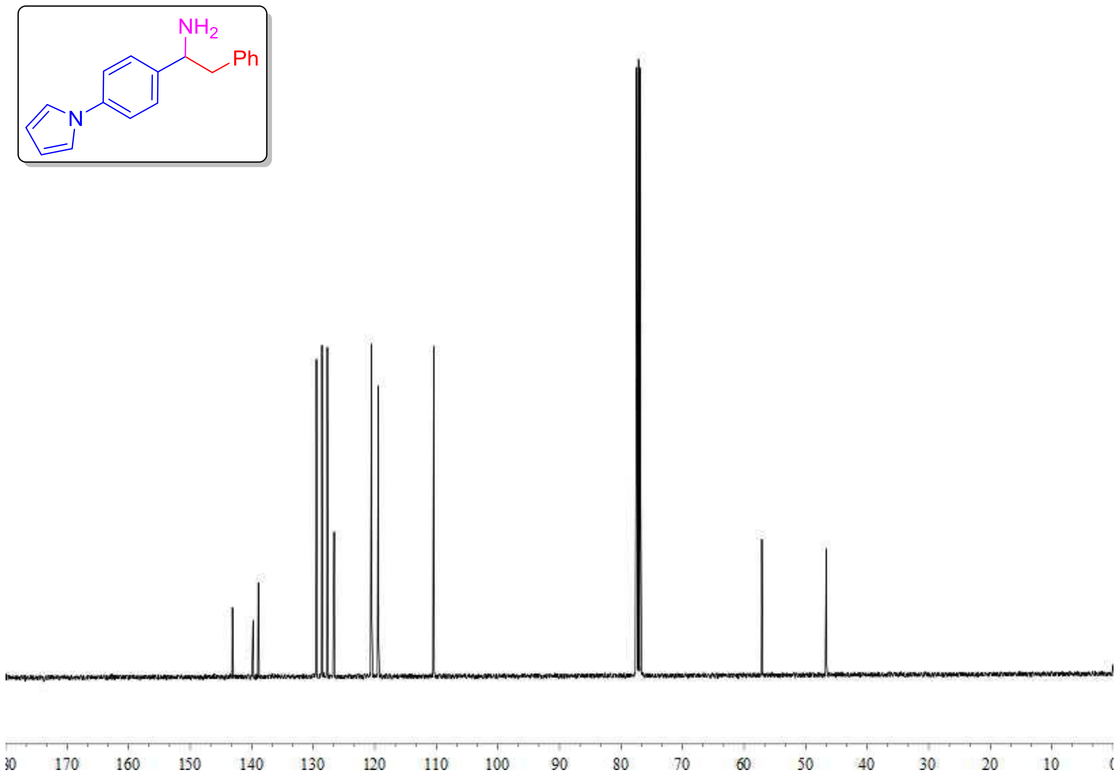

Supplementary Figure 47. ^{1}H **NMR Spectrum of 3xa (400 MHz,** $CDCl_3$**)**

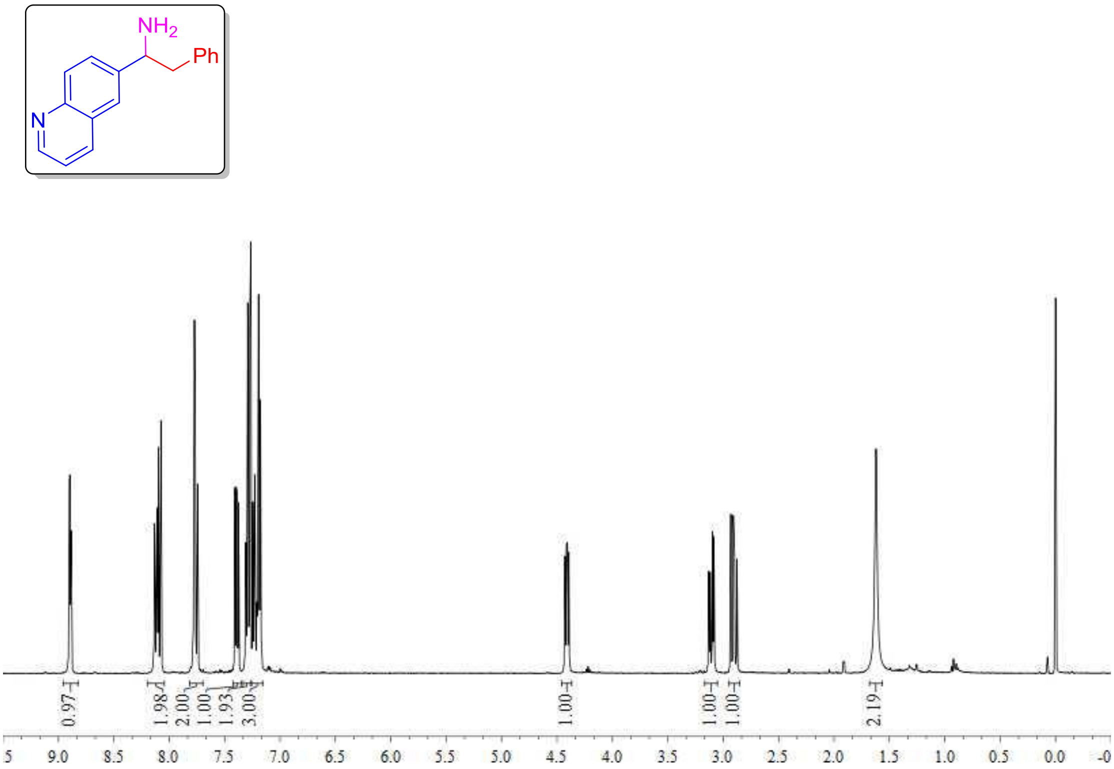

Supplementary Figure 48. ^{13}C **NMR Spectrum of 3xa (101 MHz,** $CDCl_3$**)**

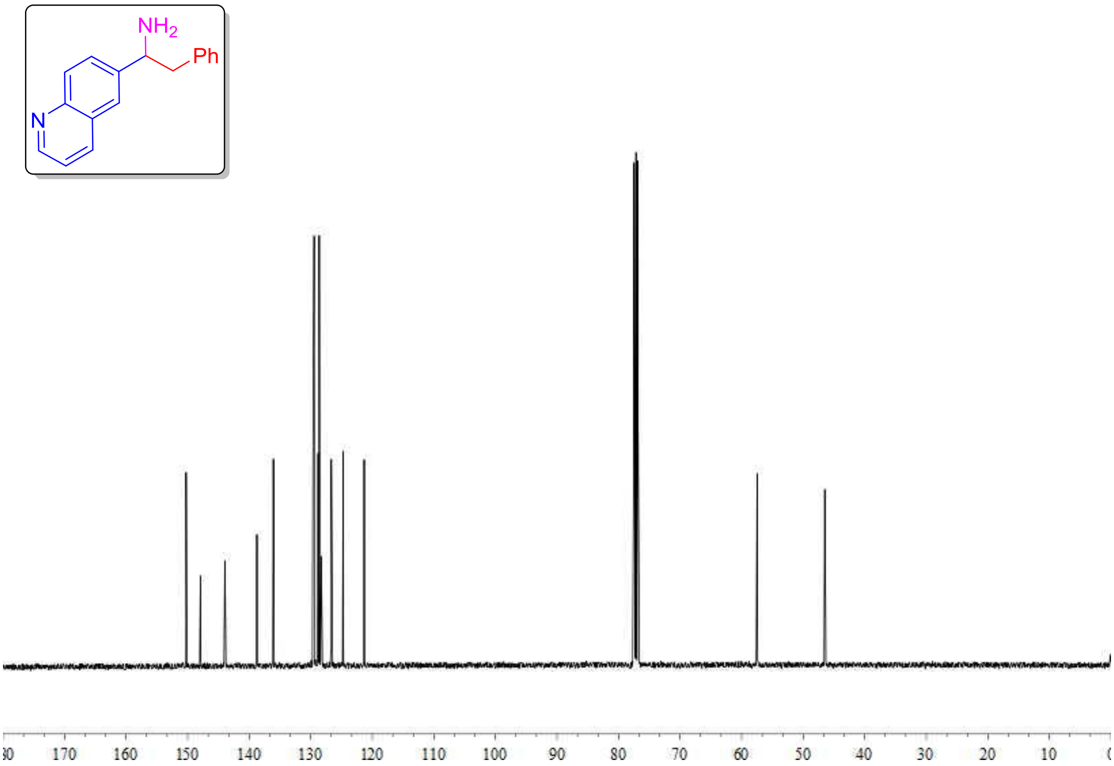

Supplementary Figure 49. ^{1}**H NMR Spectrum of 3ab (400 MHz, CDCl$_3$)**

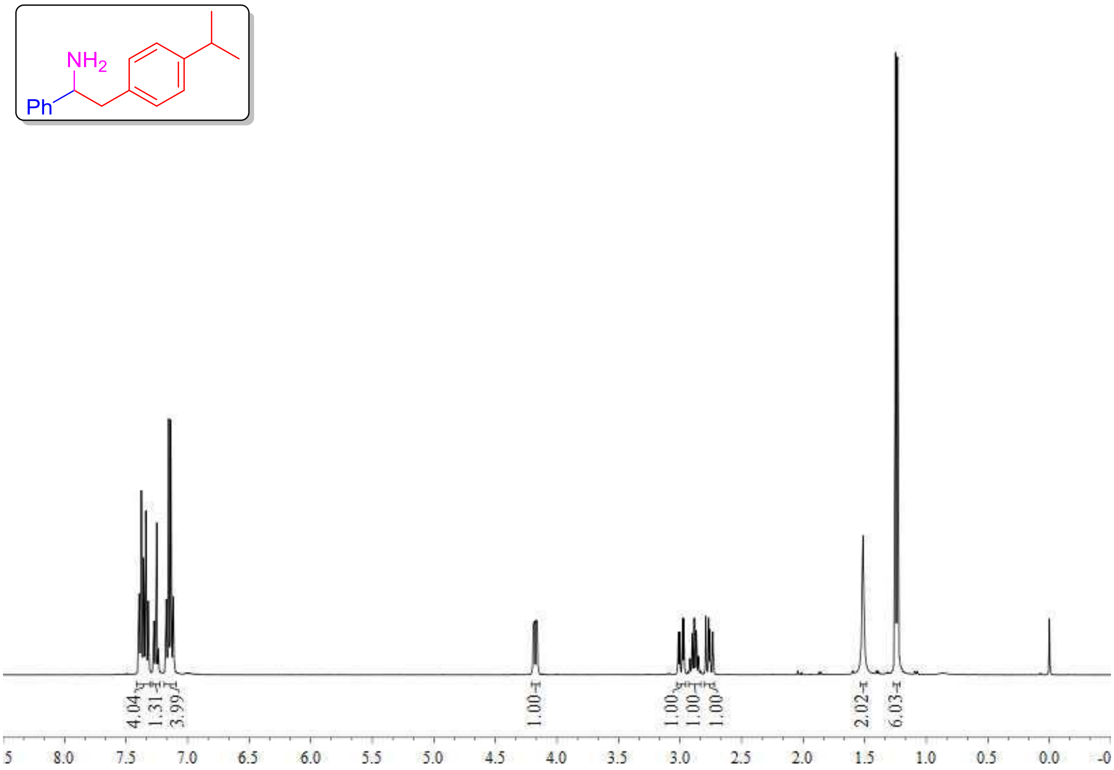

Supplementary Figure 50. ^{13}**C NMR Spectrum of 3ab (101 MHz, CDCl$_3$)**

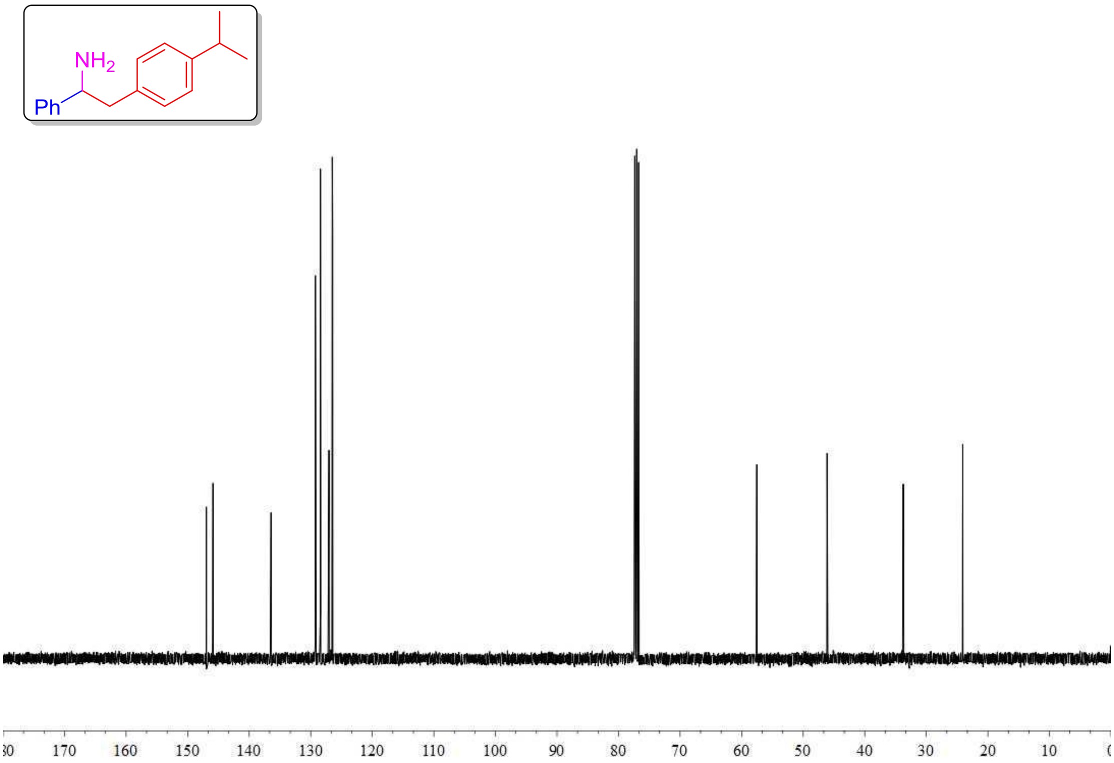

Supplementary Figure 51. ^{1}H NMR Spectrum of 3ac (400 MHz, $CDCl_3$)

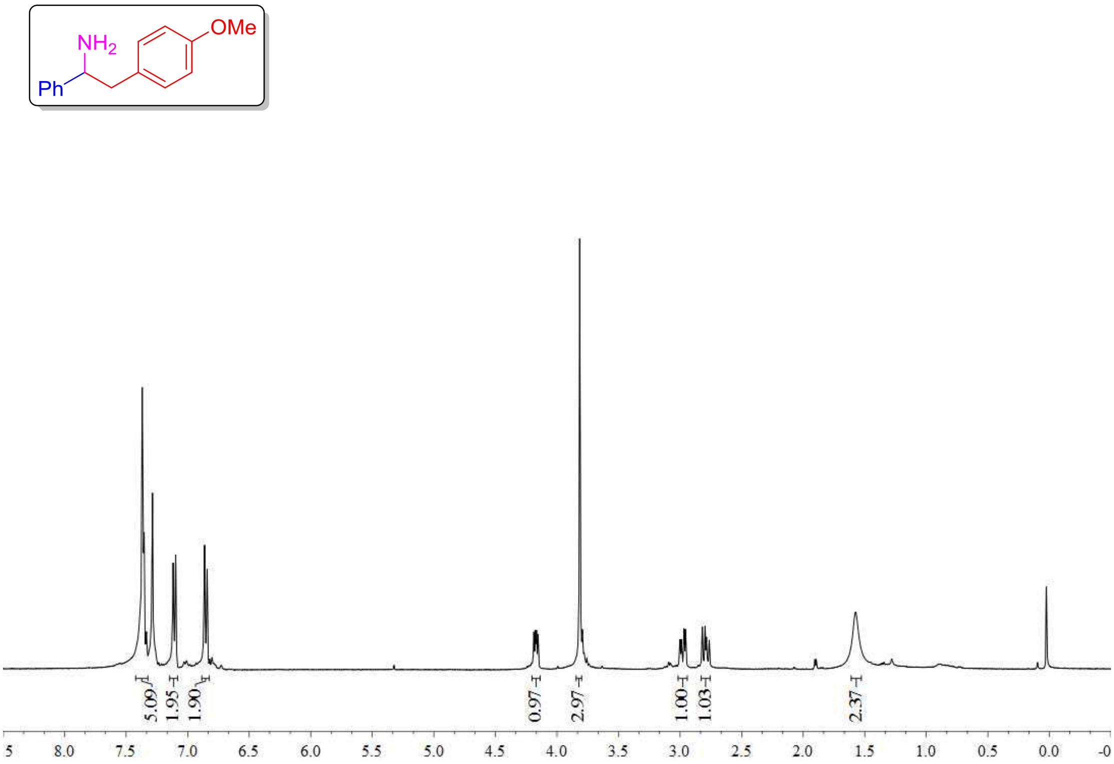

Supplementary Figure 52. ^{13}C NMR Spectrum of 3ac (101 MHz, $CDCl_3$)

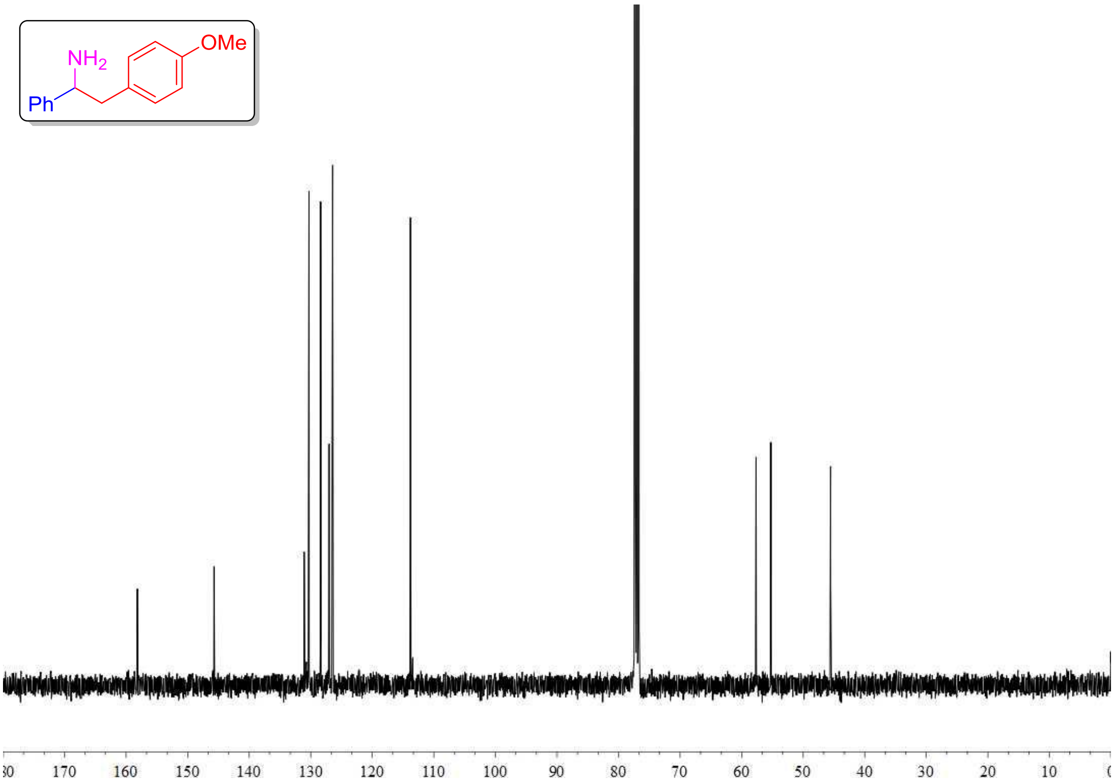

Supplementary Figure 53. ^{1}**H NMR Spectrum of 3ad (400 MHz, CDCl$_3$)**

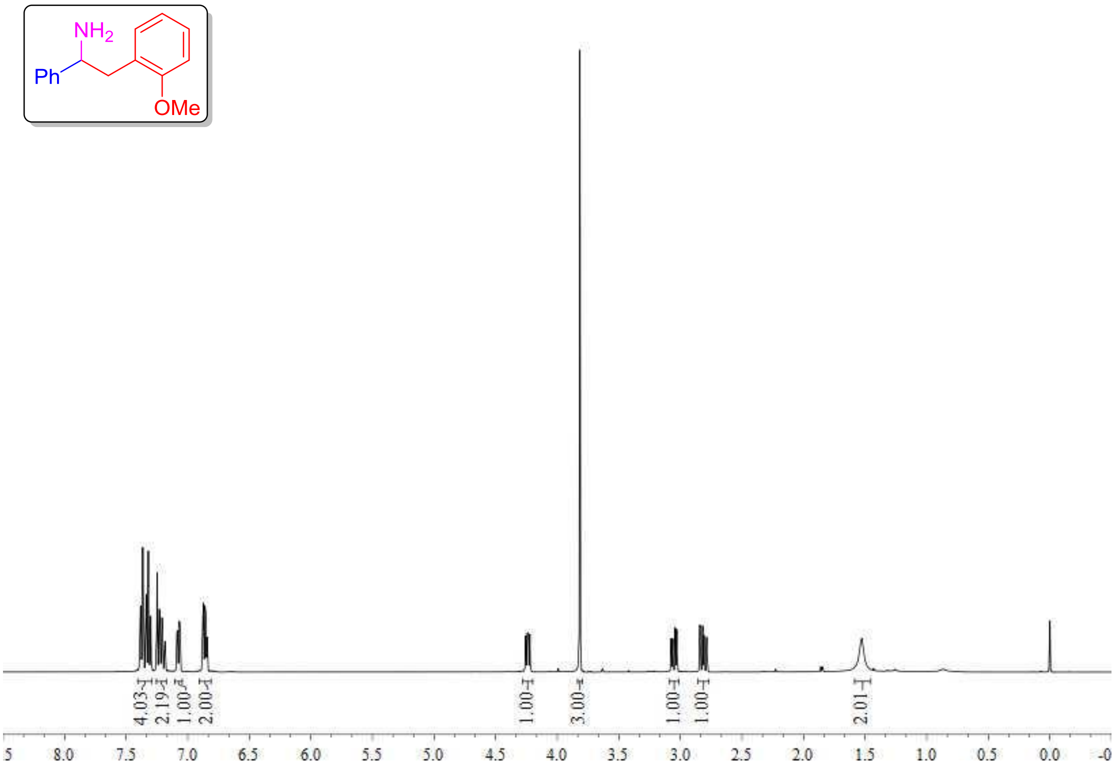

Supplementary Figure 54. ^{13}**C NMR Spectrum of 3ad (101 MHz, CDCl$_3$)**

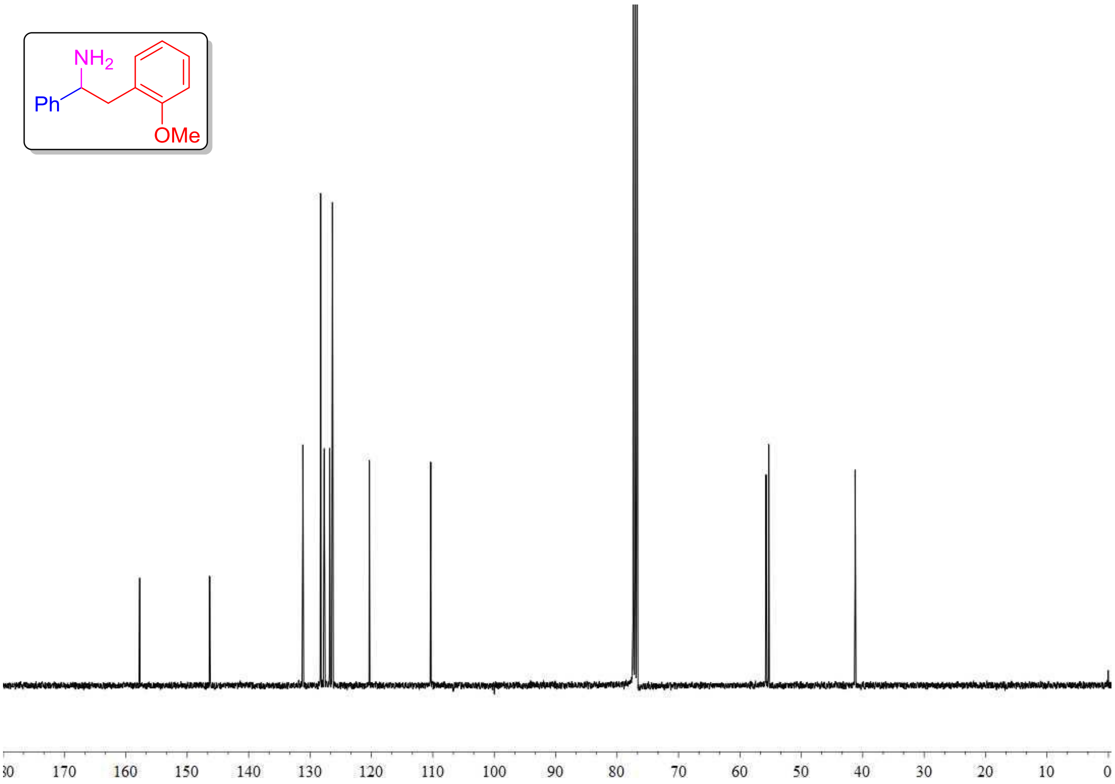

Supplementary Figure 55. **^{1}H NMR Spectrum of 3ae (400 MHz, $CDCl_3$)**

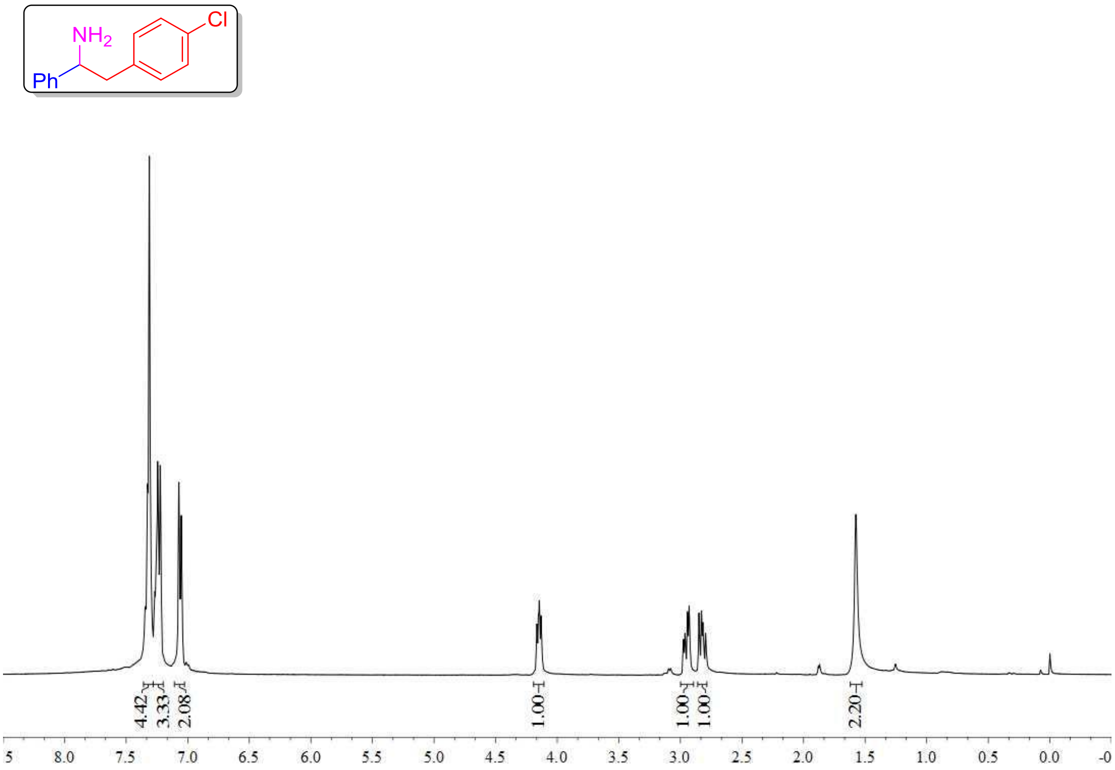

Supplementary Figure 56. **^{13}C NMR Spectrum of 3ae (101 MHz, $CDCl_3$)**

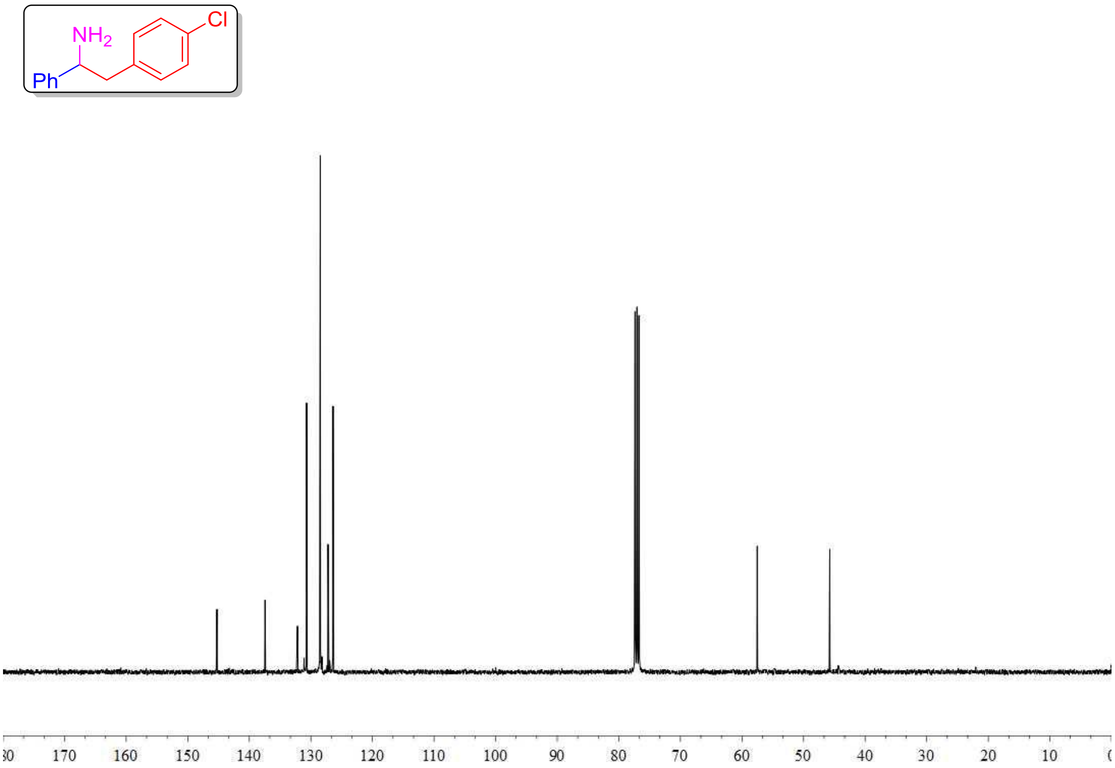

Supplementary Figure 57. ^{1}H **NMR Spectrum of 3af (400 MHz,** $CDCl_3$**)**

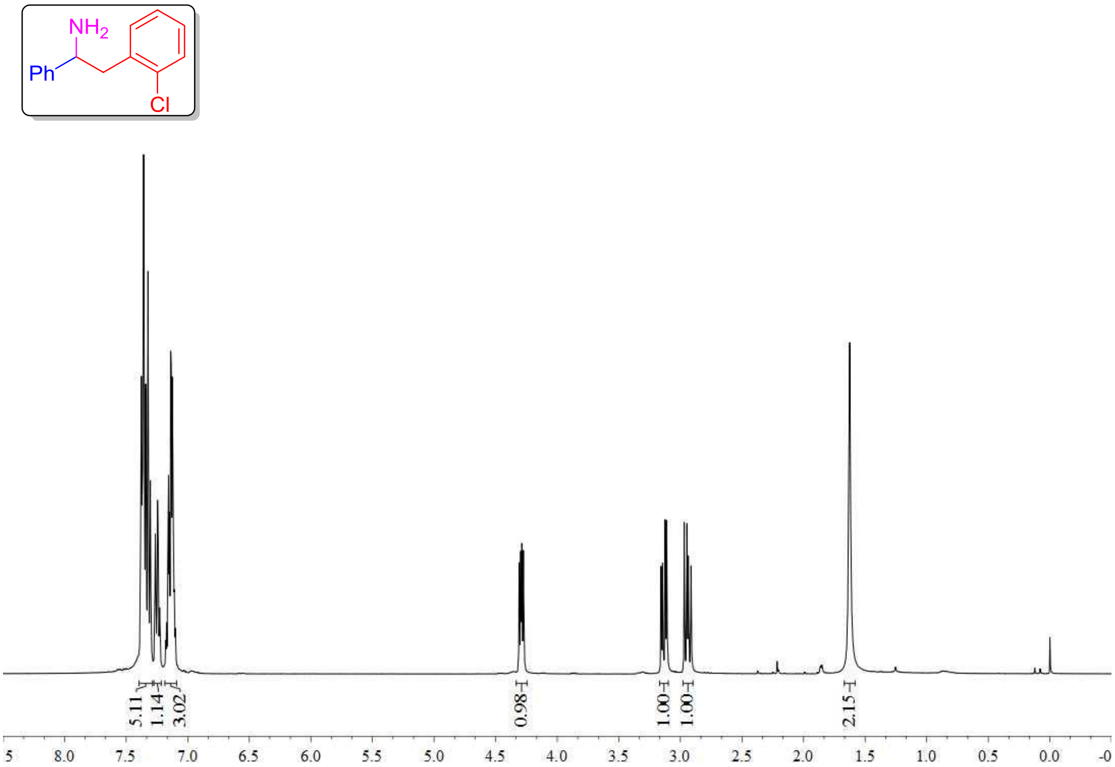

Supplementary Figure 58. ^{13}C **NMR Spectrum of 3af (101 MHz,** $CDCl_3$**)**

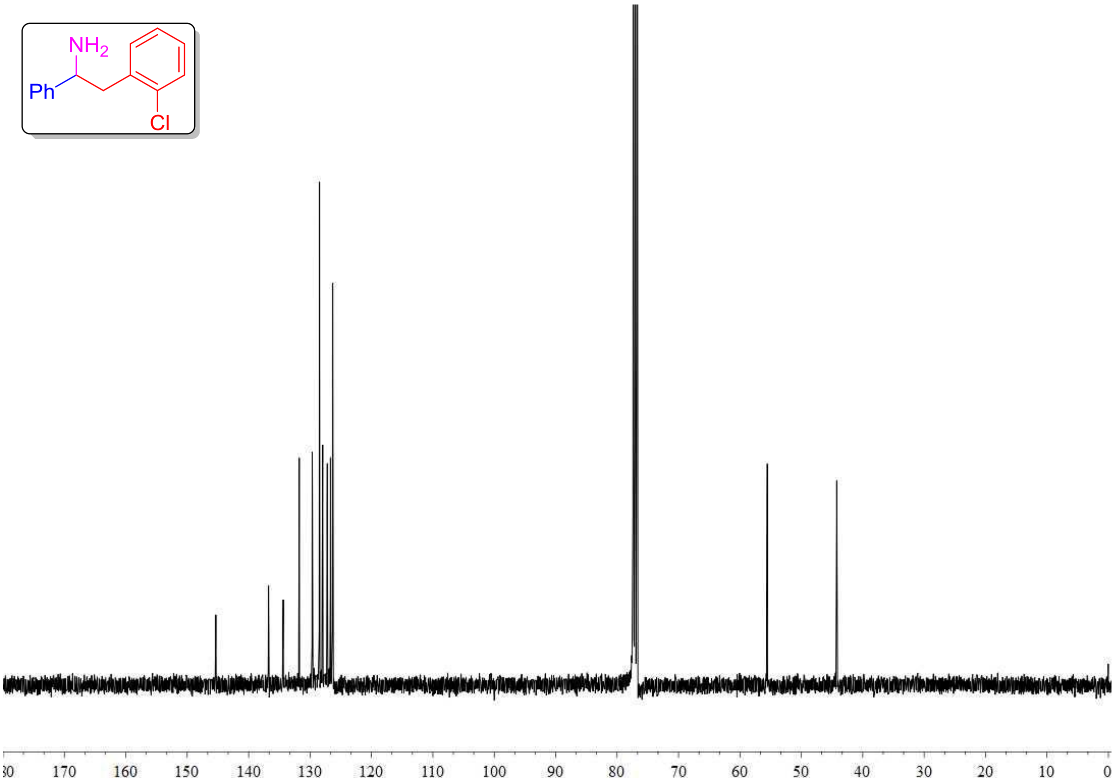

Supplementary Figure 59. **^{1}H NMR Spectrum of 3ag (400 MHz, $CDCl_3$)**

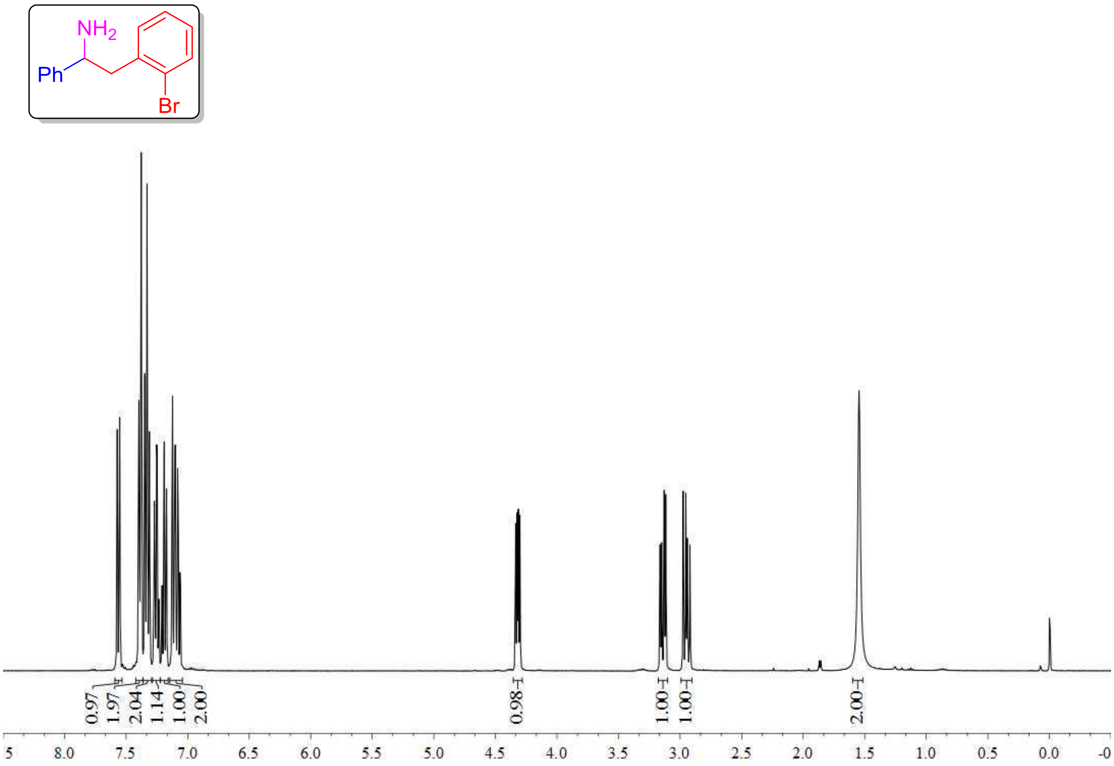

Supplementary Figure 60. **^{13}C NMR Spectrum of 3ag (101 MHz, $CDCl_3$)**

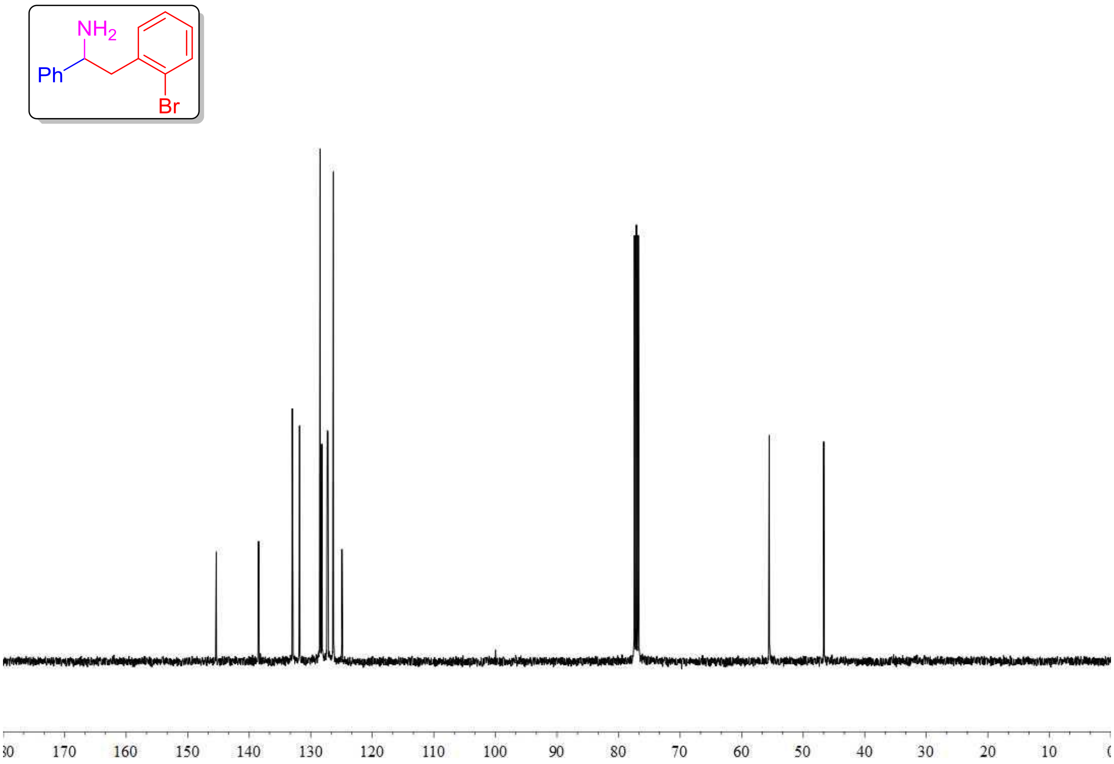

Supplementary Figure 61. ^{1}**H NMR Spectrum of 3ah (400 MHz, CDCl$_3$)**

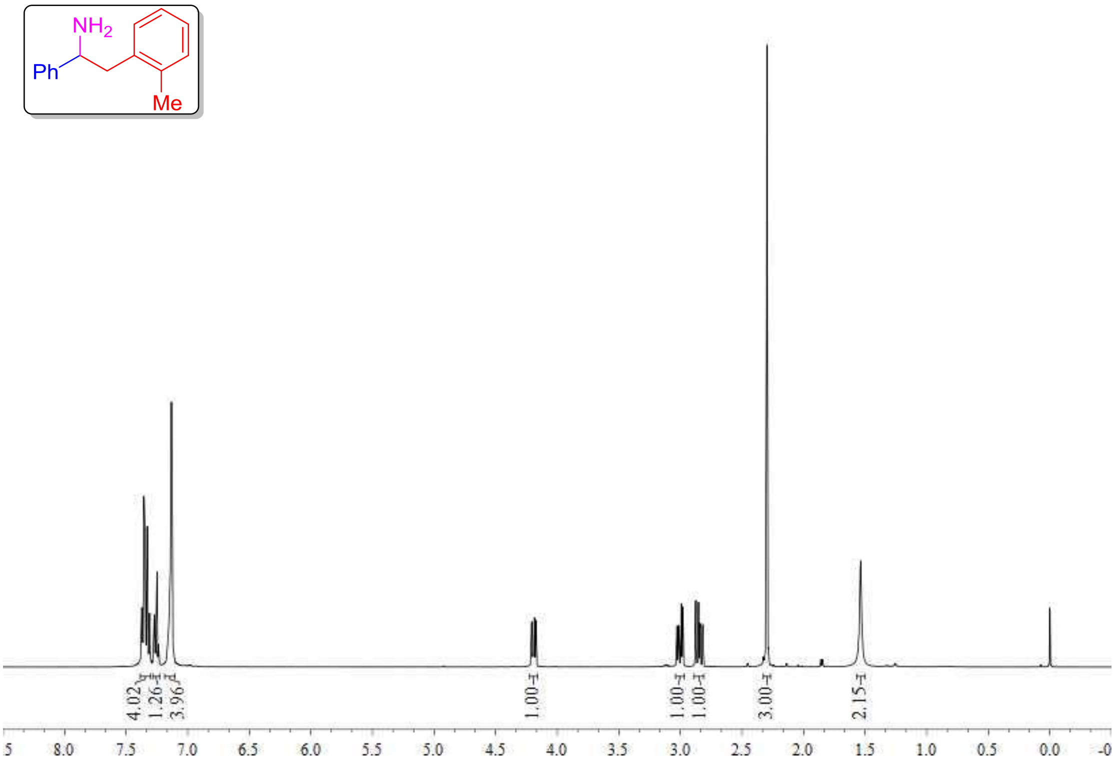

Supplementary Figure 62. ^{13}**C NMR Spectrum of 3ah (101 MHz, CDCl$_3$)**

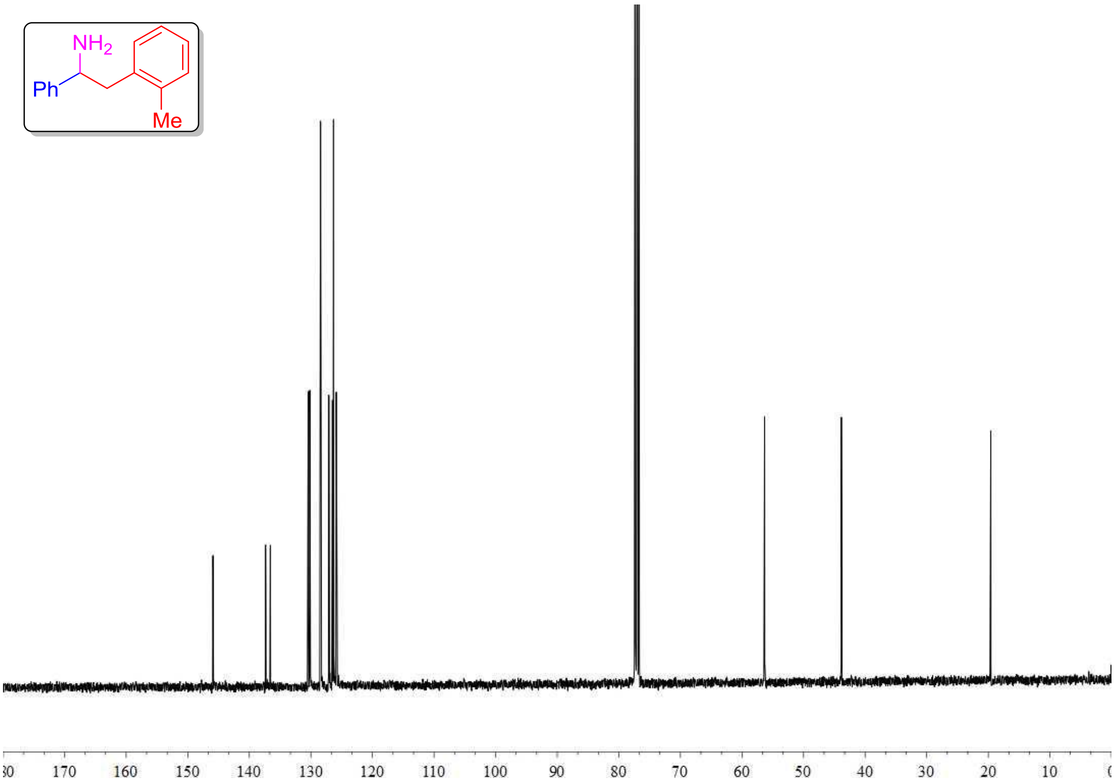

Supplementary Figure 63. ^{1}H NMR Spectrum of 3ai (400 MHz, $CDCl_3$)

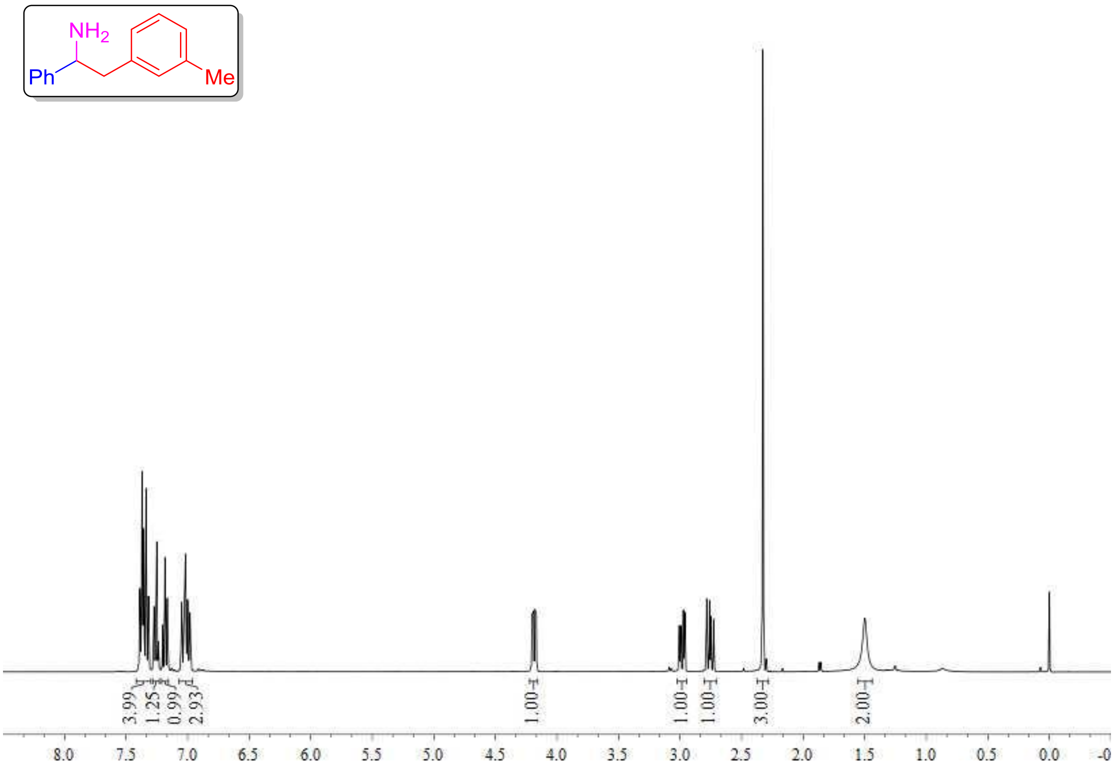

Supplementary Figure 64. ^{13}C NMR Spectrum of 3ai (101 MHz, $CDCl_3$)

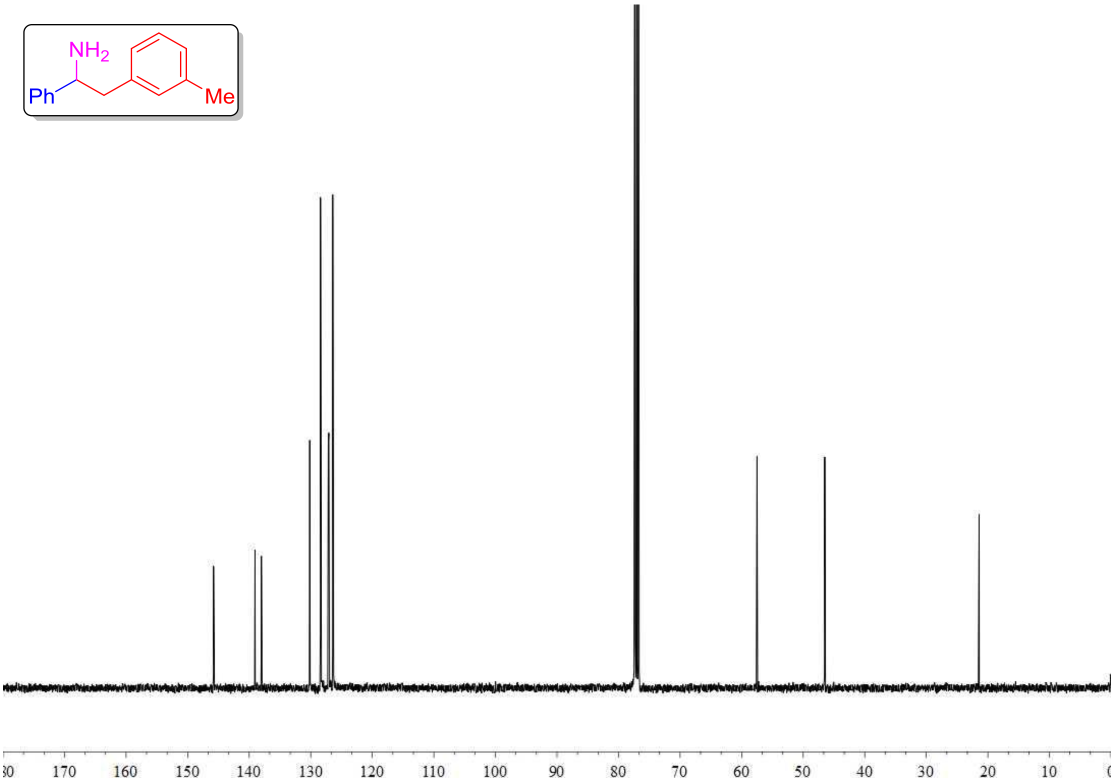

Supplementary Figure 65. ^{1}H NMR Spectrum of 3aj (400 MHz, $CDCl_3$)

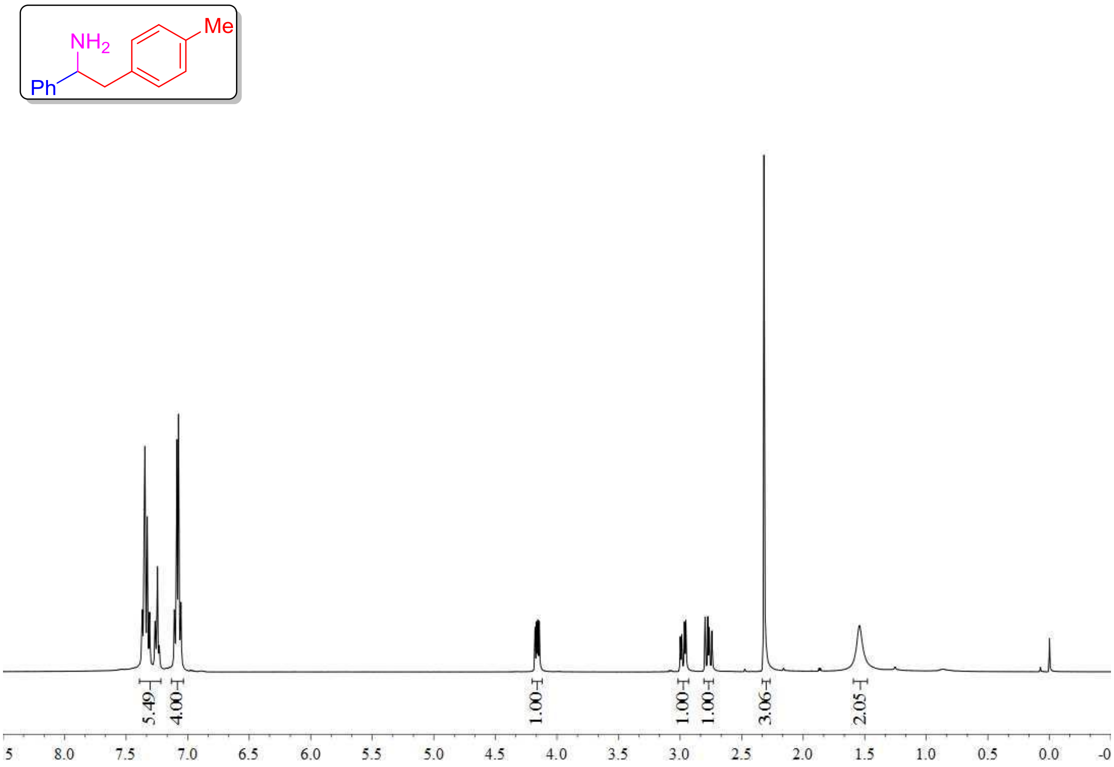

Supplementary Figure 66. ^{13}C NMR Spectrum of 3aj (101 MHz, $CDCl_3$)

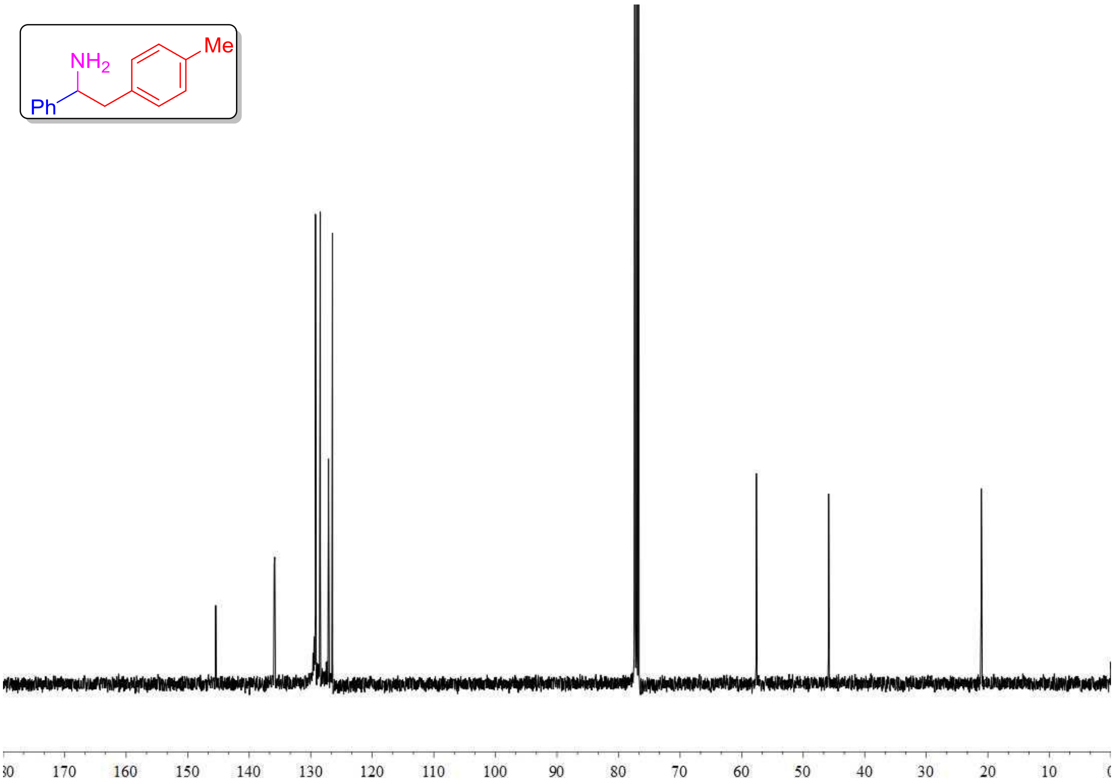

Supplementary Figure 67. ^{1}**H NMR Spectrum of 3ak (400 MHz, $CDCl_3$)**

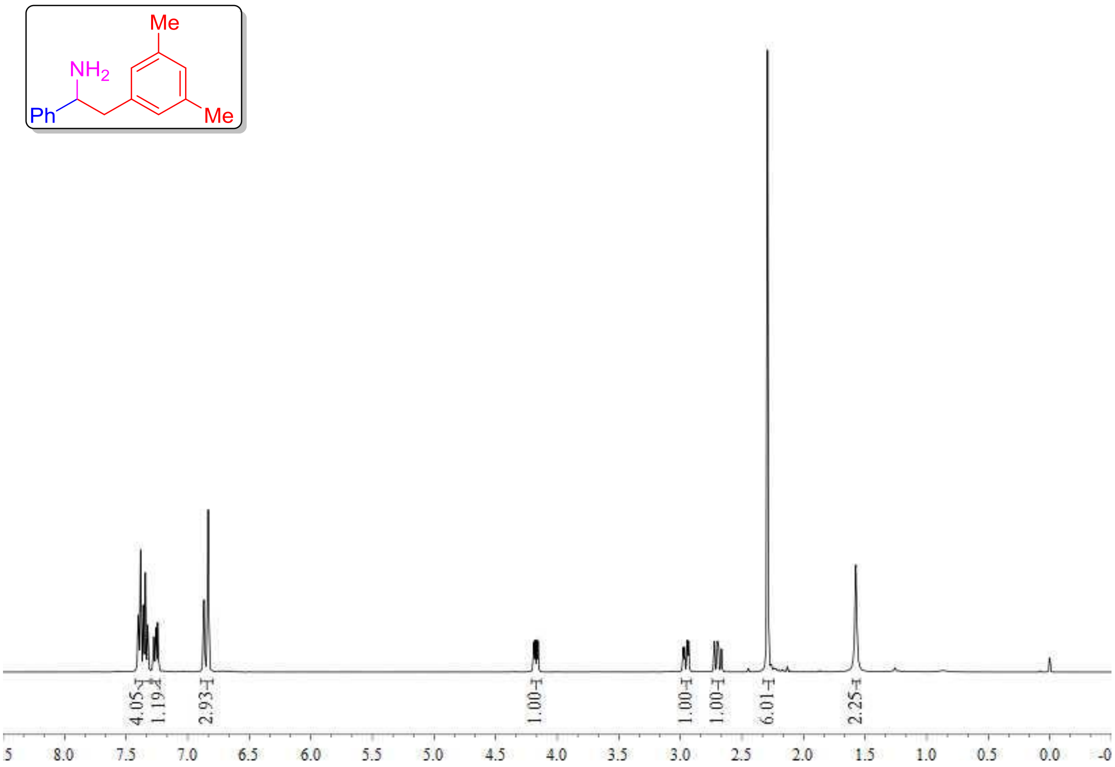

Supplementary Figure 68. ^{13}**C NMR Spectrum of 3ak (101 MHz, $CDCl_3$)**

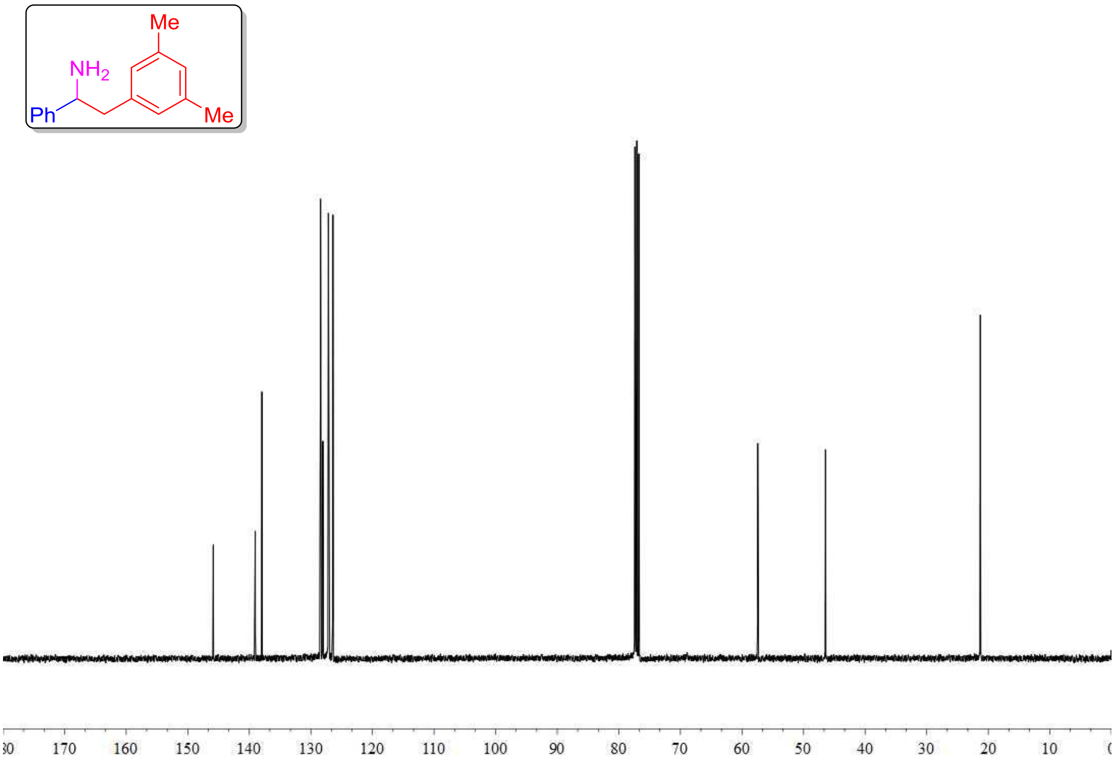

Supplementary Figure 69. ^{1}**H NMR Spectrum of 3al (400 MHz, $CDCl_3$)**

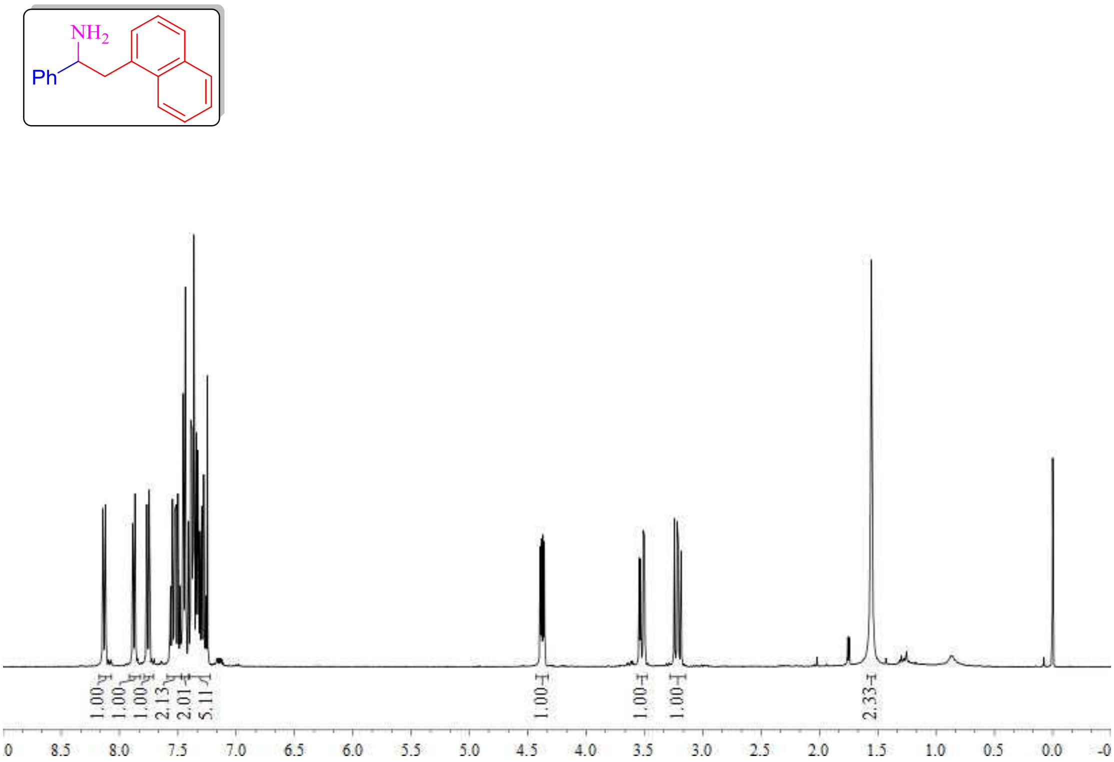

Supplementary Figure 70. ^{13}**C NMR Spectrum of 3al (101 MHz, $CDCl_3$)**

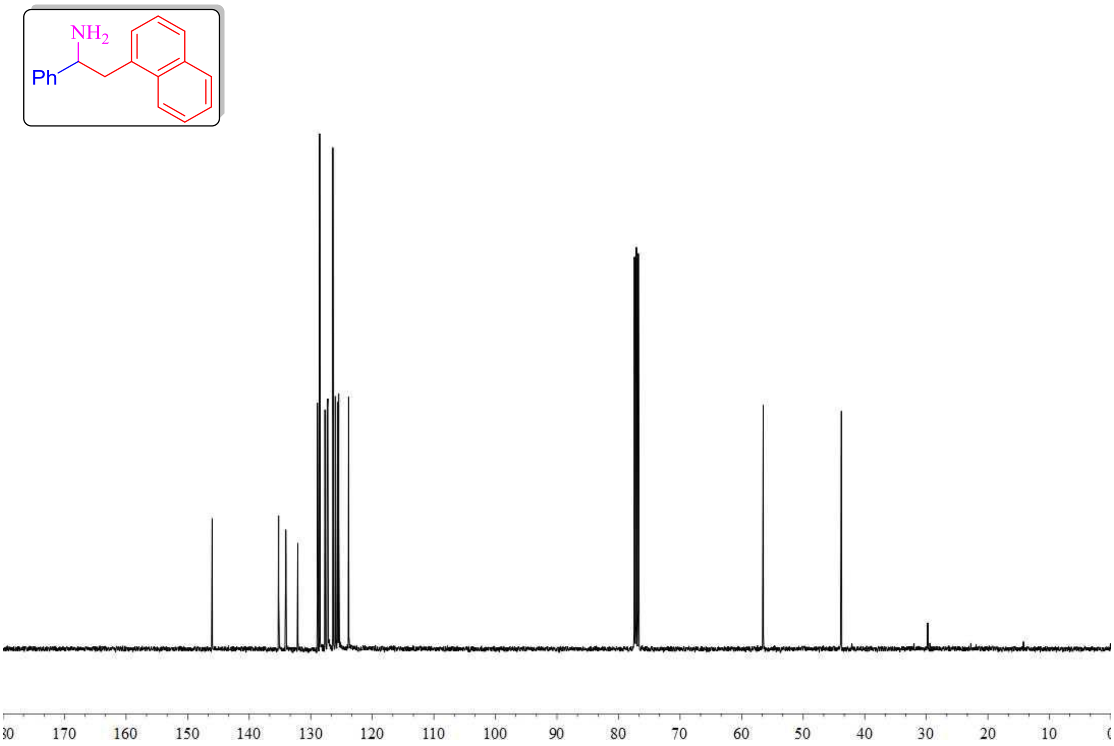

Supplementary Figure 71. ^{1}H NMR Spectrum of 3vg (400 MHz, $CDCl_3$)

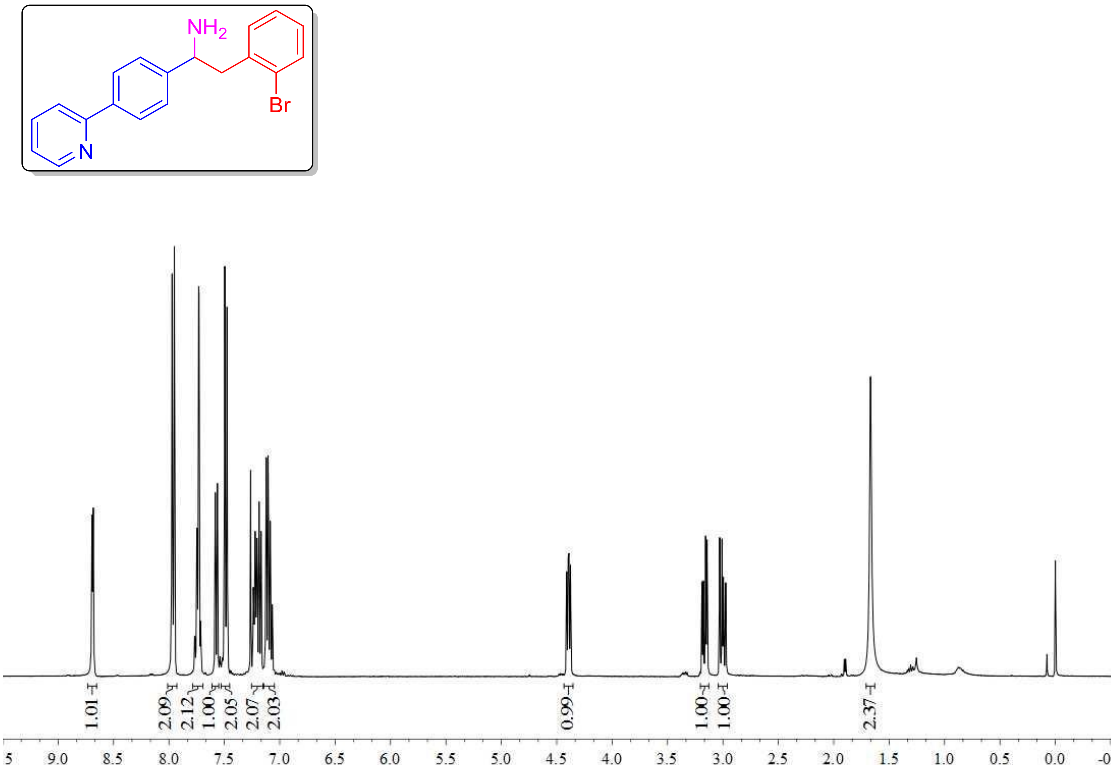

Supplementary Figure 72. ^{13}C NMR Spectrum of 3vg (101 MHz, $CDCl_3$)

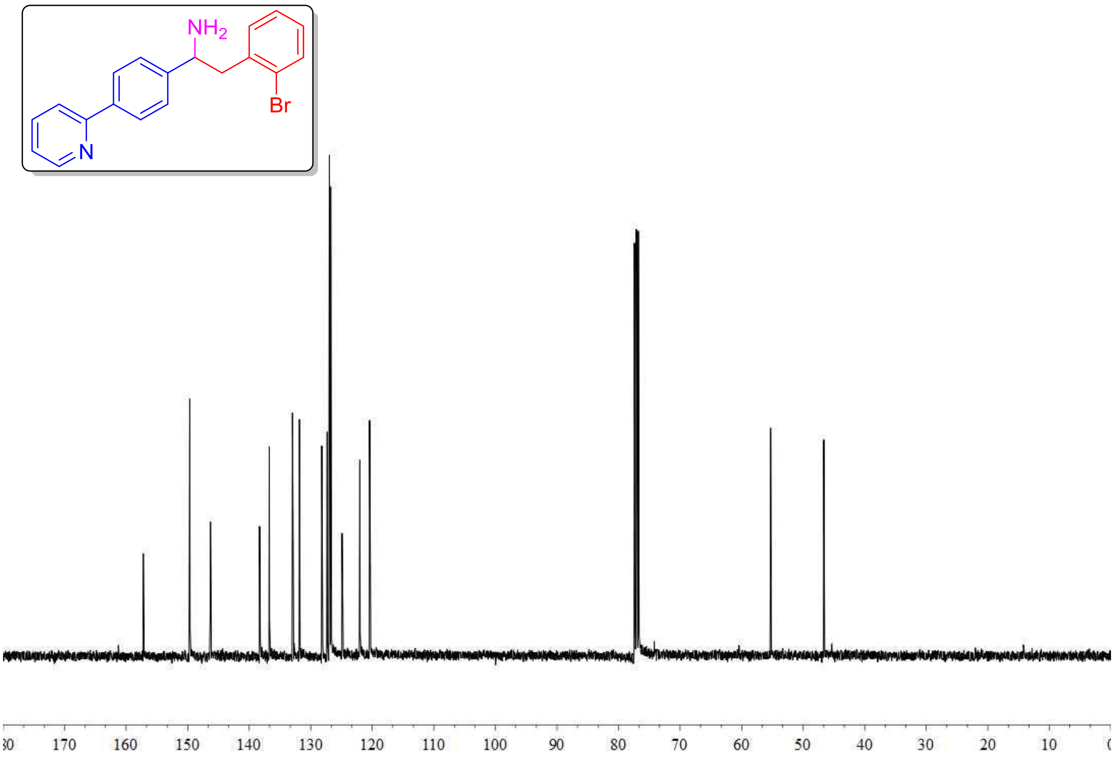

Supplementary Figure 73. ^{1}H NMR Spectrum of 3ng (400 MHz, $CDCl_3$)

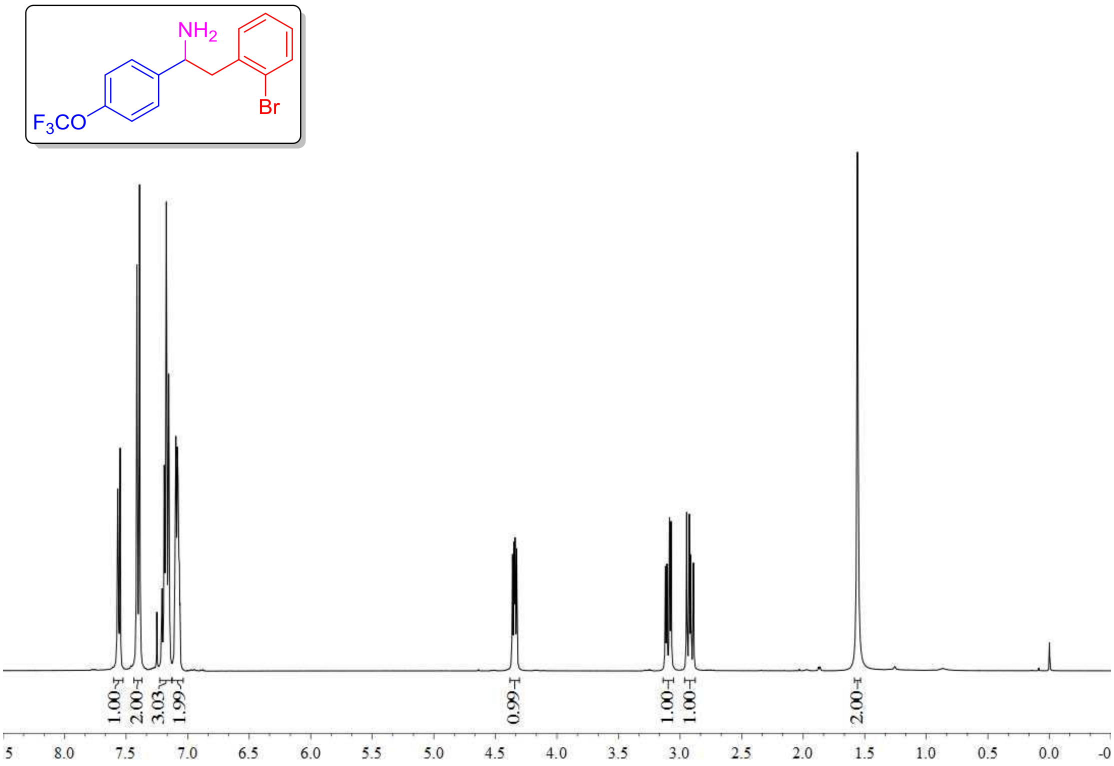

Supplementary Figure 74. ^{13}C NMR Spectrum of 3ng (101 MHz, $CDCl_3$)

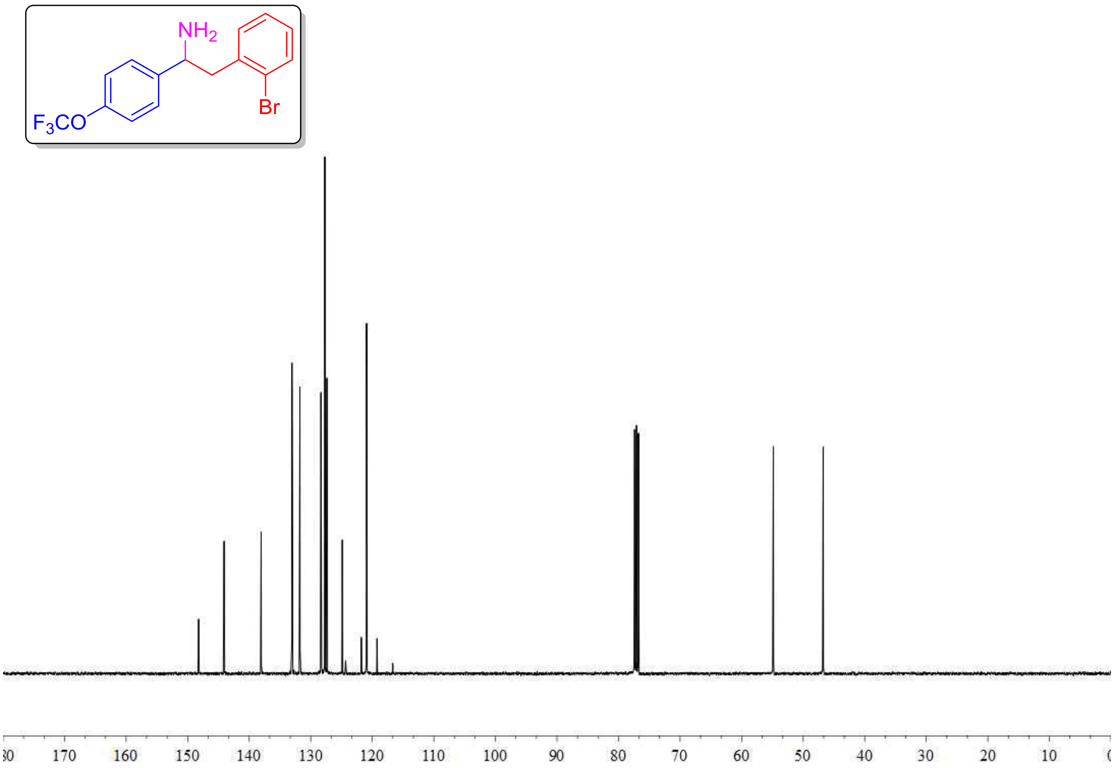

Supplementary Figure 75. ^{1}H NMR Spectrum of 3qg (400 MHz, $CDCl_3$)

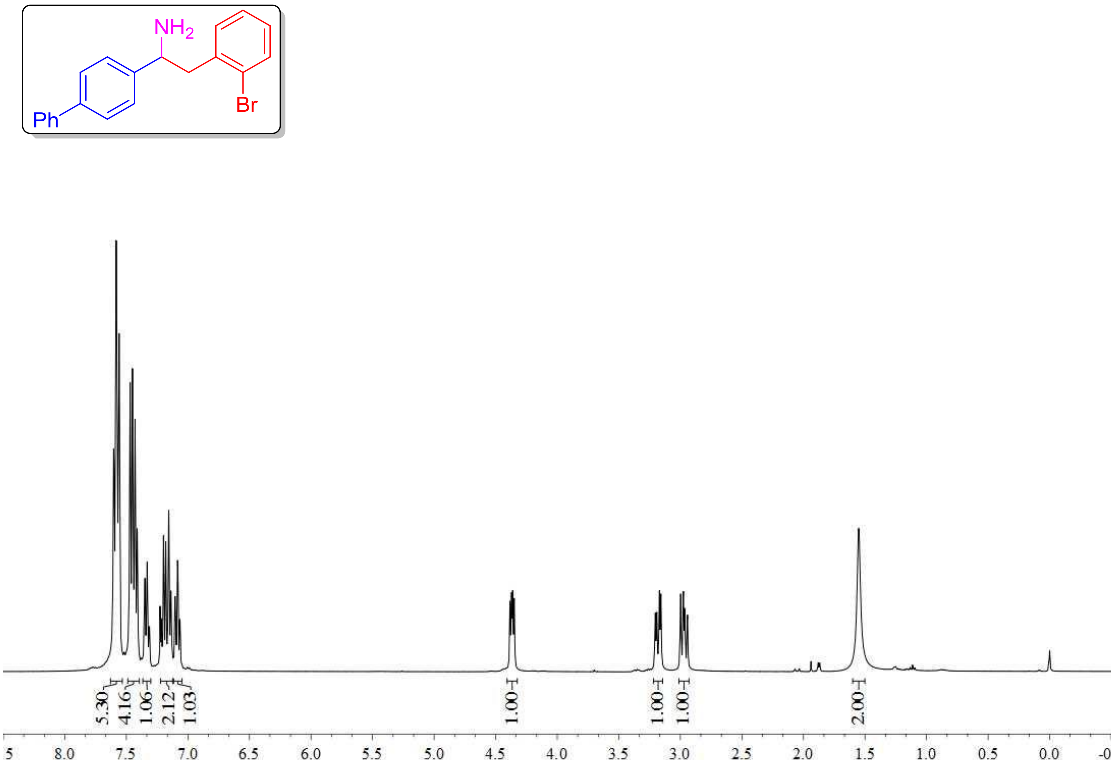

Supplementary Figure 76. ^{13}C NMR Spectrum of 3qg (101 MHz, $CDCl_3$)

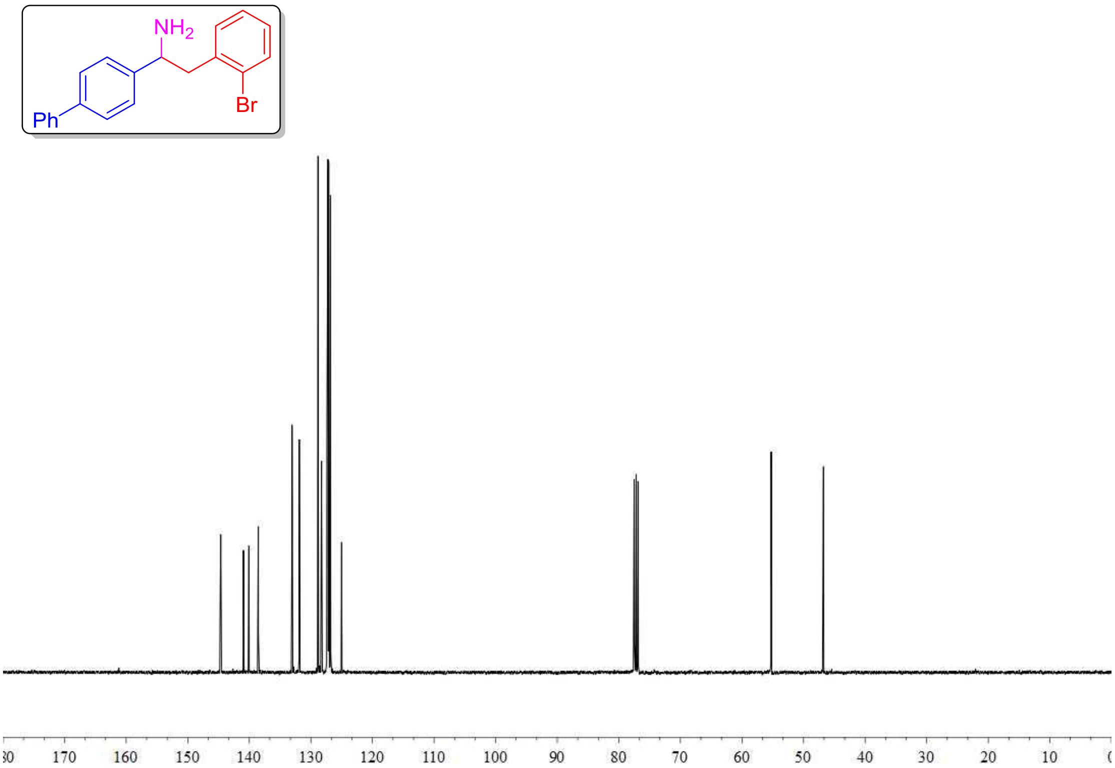

Supplementary Figure 77. ^{1}**H NMR Spectrum of 3'aa (400 MHz, CD_3OD)**

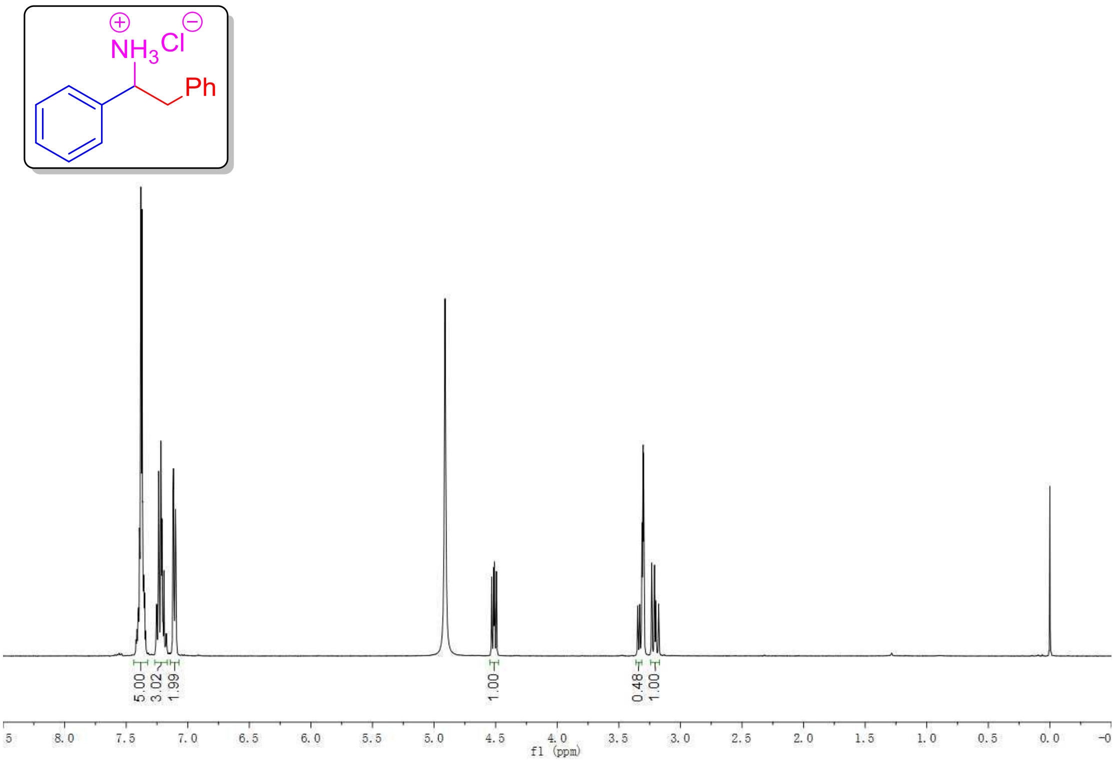

Supplementary Figure 78. ^{13}**C NMR Spectrum of 3'aa (101 MHz, CD_3OD)**

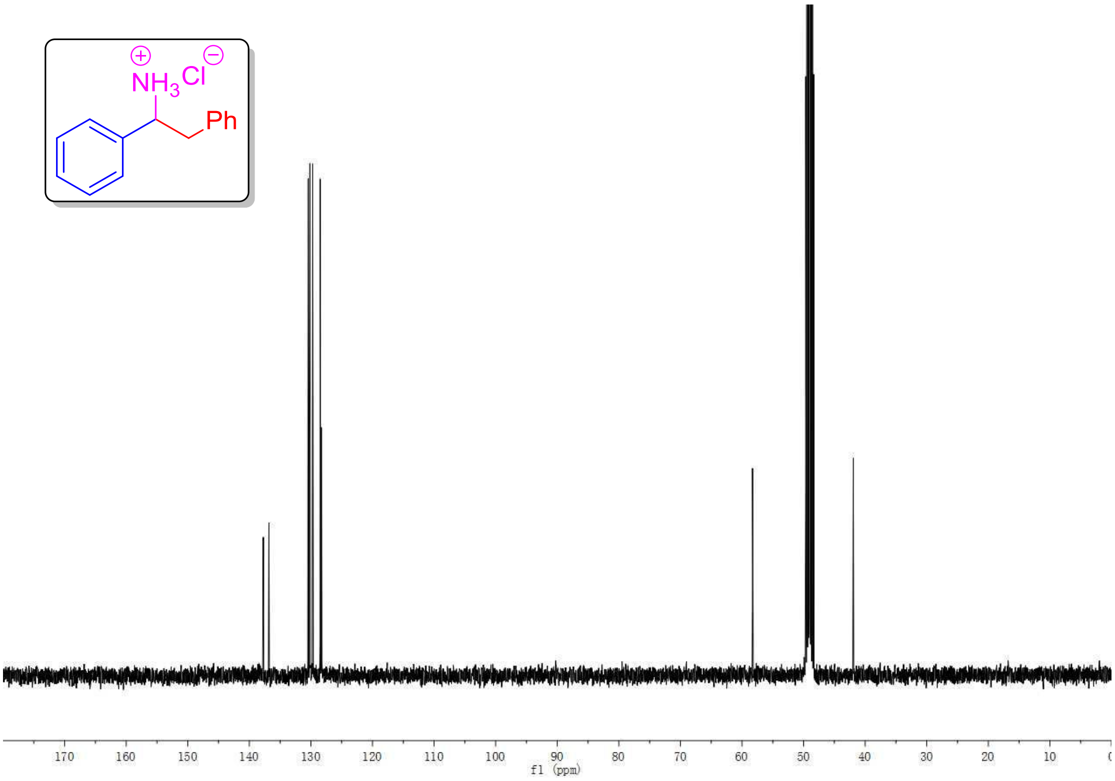

Supplementary Figure 79. ^{1}H NMR Spectrum of 3ag (400 MHz, $CDCl_3$)

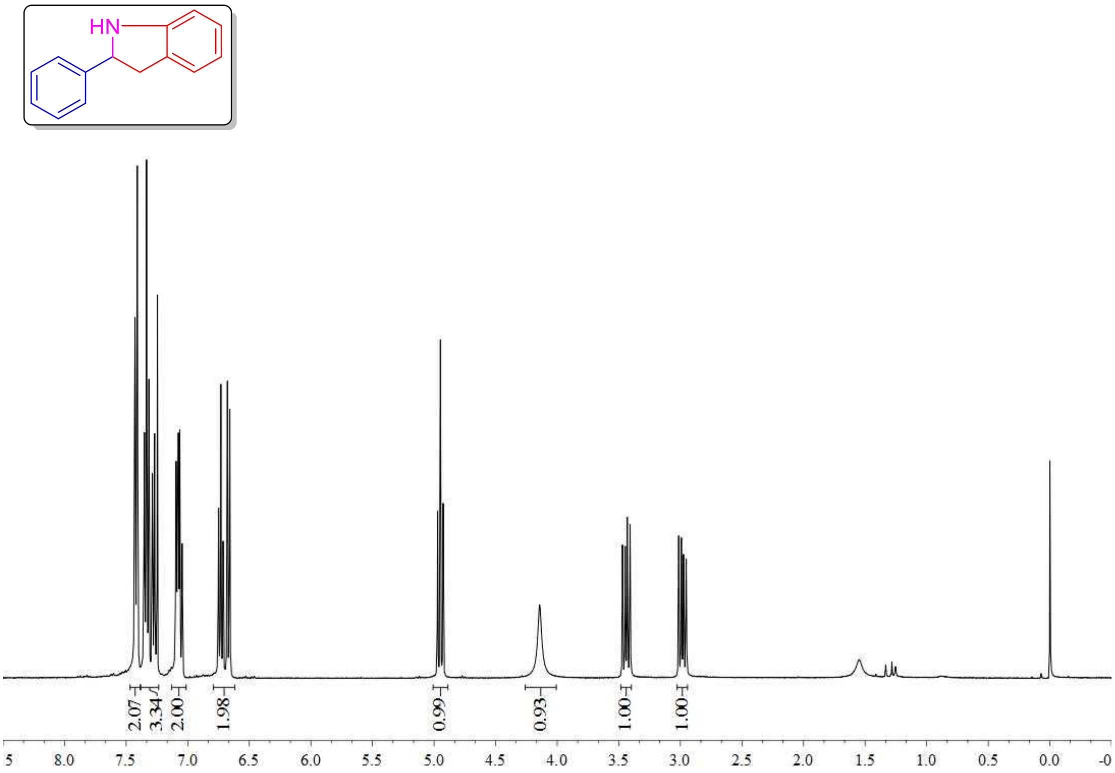

Supplementary Figure 80. ^{13}C NMR Spectrum of 3ag (101 MHz, $CDCl_3$)

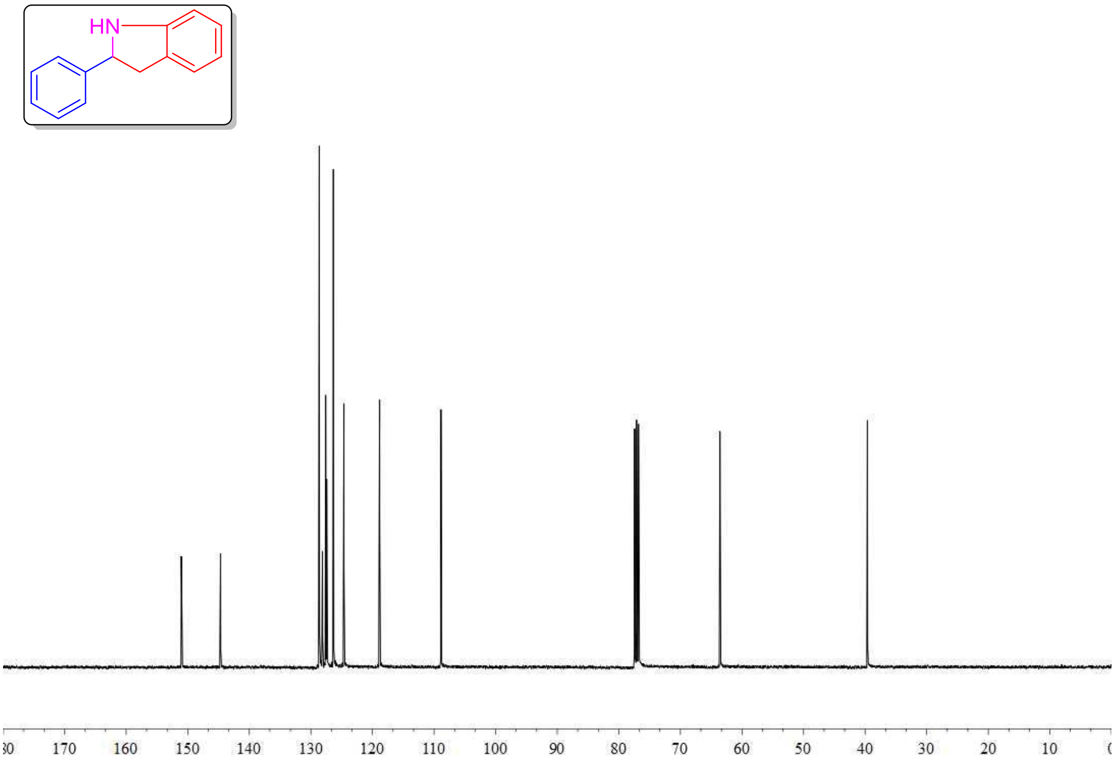

Supplementary Figure 81. ^{1}**H NMR Spectrum of 3vg (400 MHz, CDCl$_3$)**

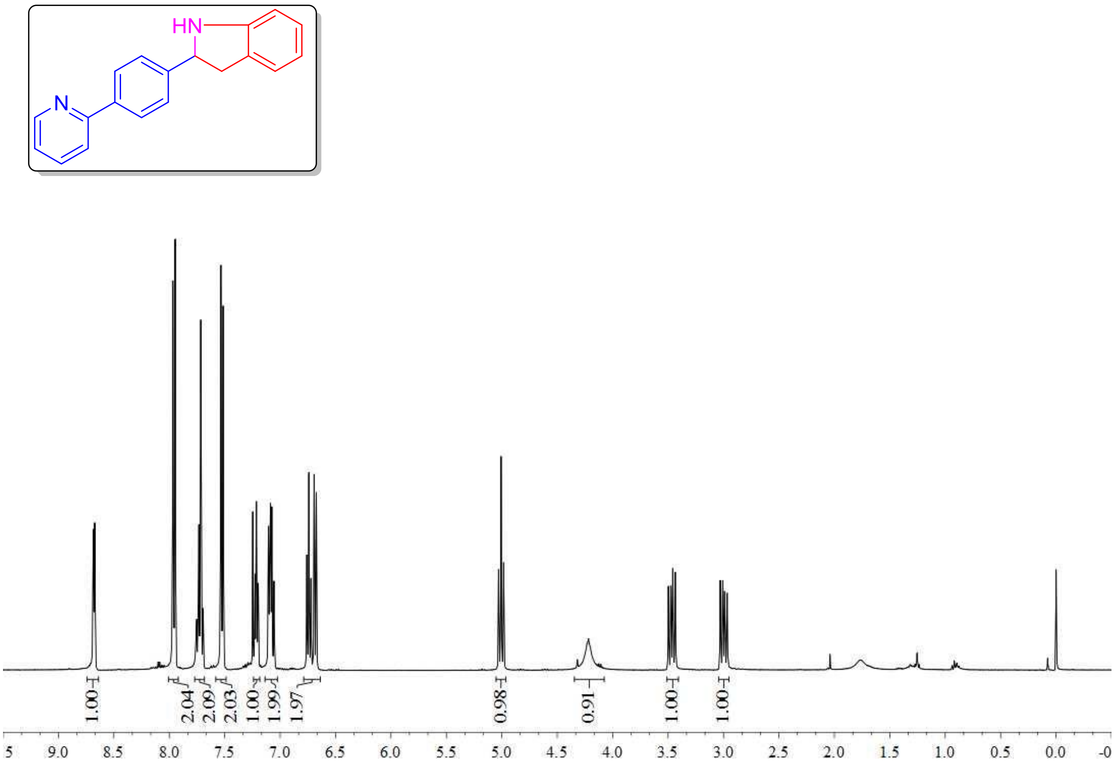

Supplementary Figure 82. ^{13}**C NMR Spectrum of 3vg (101 MHz, CDCl$_3$)**

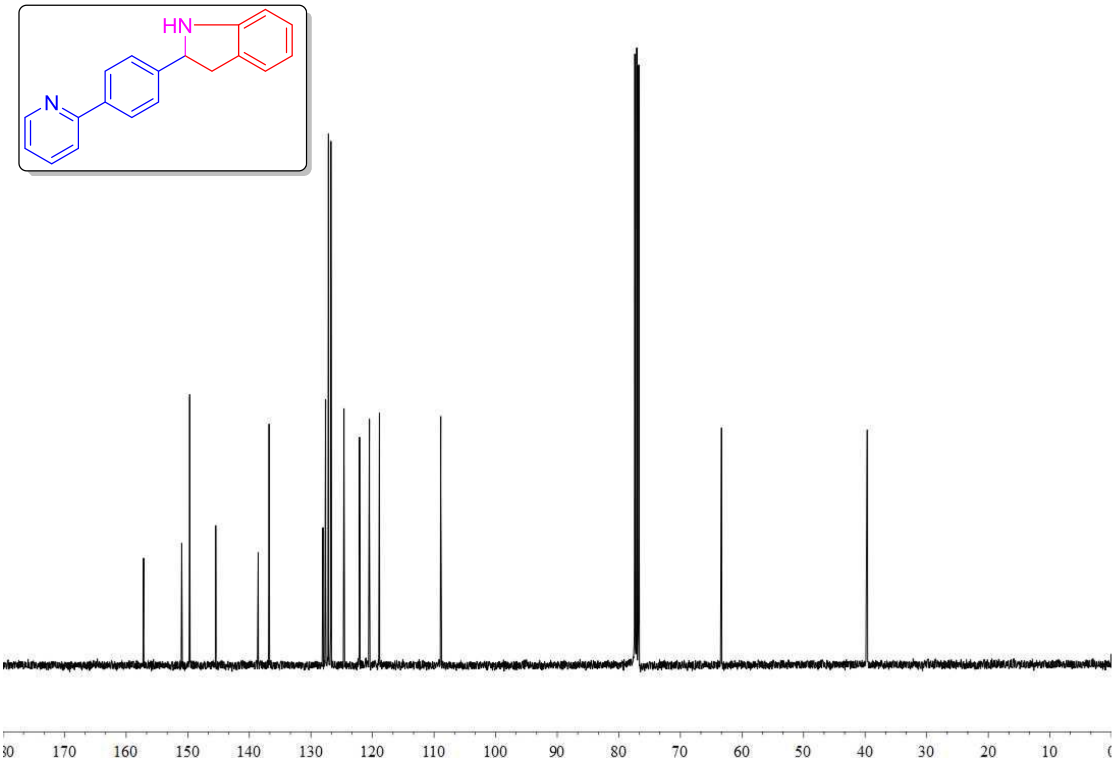

Supplementary Figure 83. **^{1}H NMR Spectrum of 3ng (400 MHz, CDCl$_3$)**

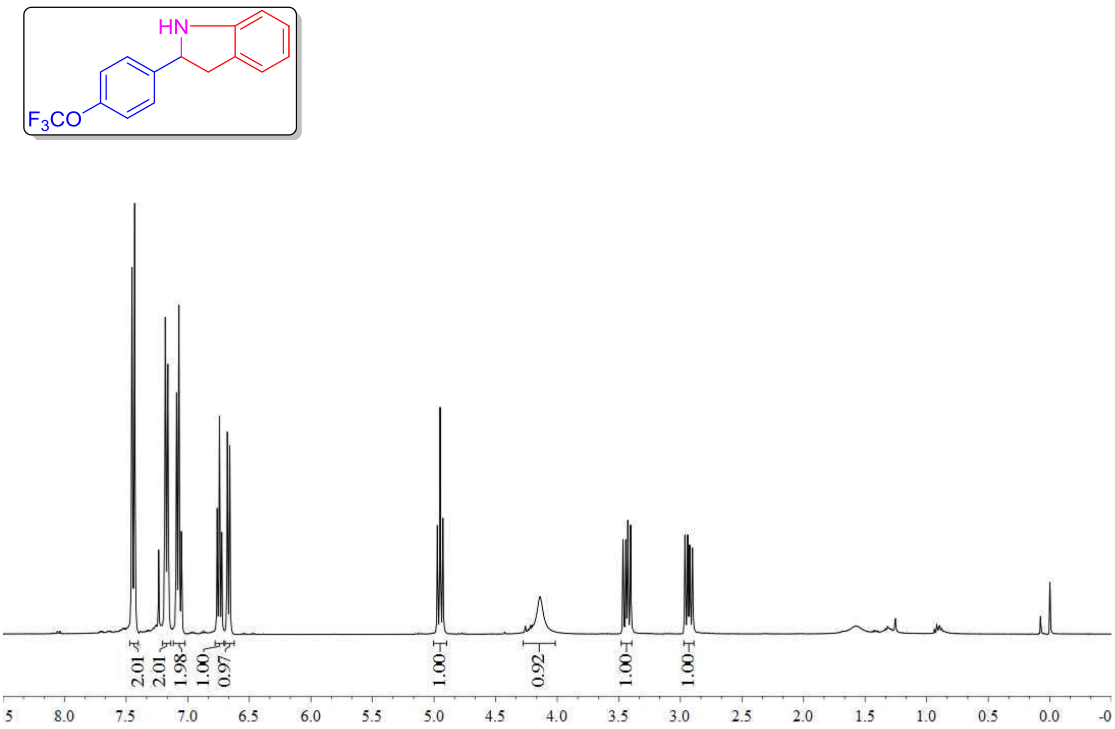

Supplementary Figure 84. **^{13}C NMR Spectrum of 3ng (101 MHz, CDCl$_3$)**

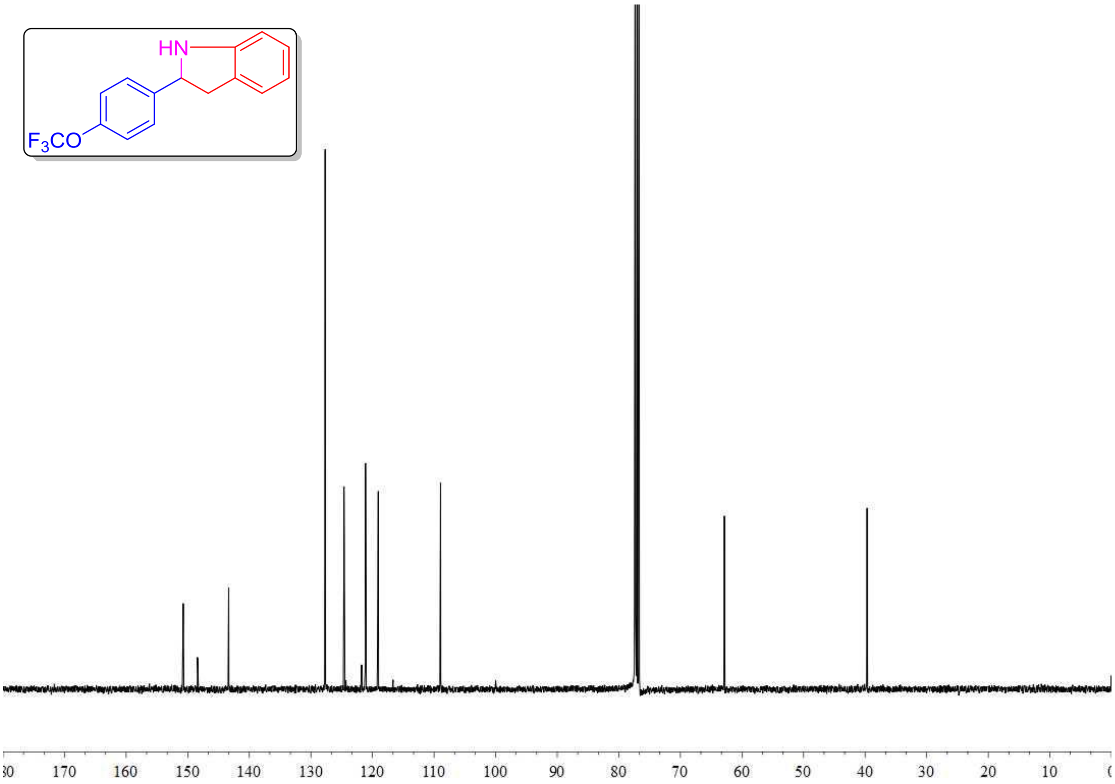

Supplementary Figure 85. ^{1}H NMR Spectrum of 3qg (400 MHz, $CDCl_3$)

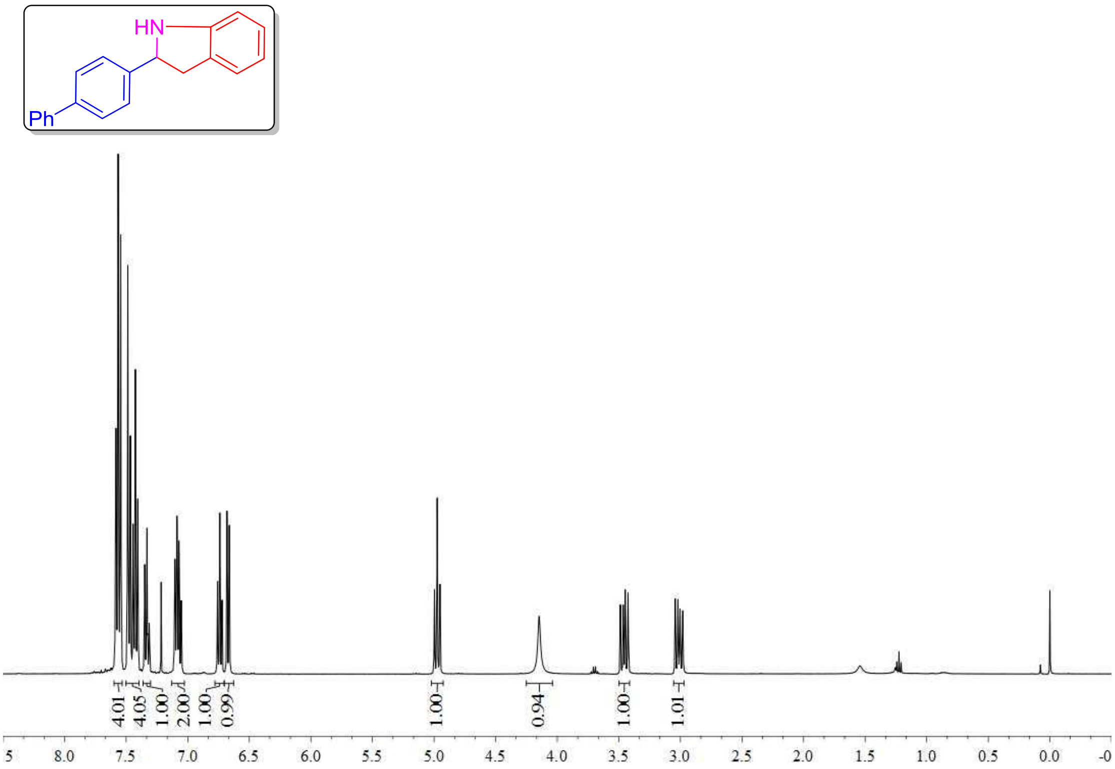

Supplementary Figure 86. ^{13}C NMR Spectrum of 3qg (101 MHz, $CDCl_3$)

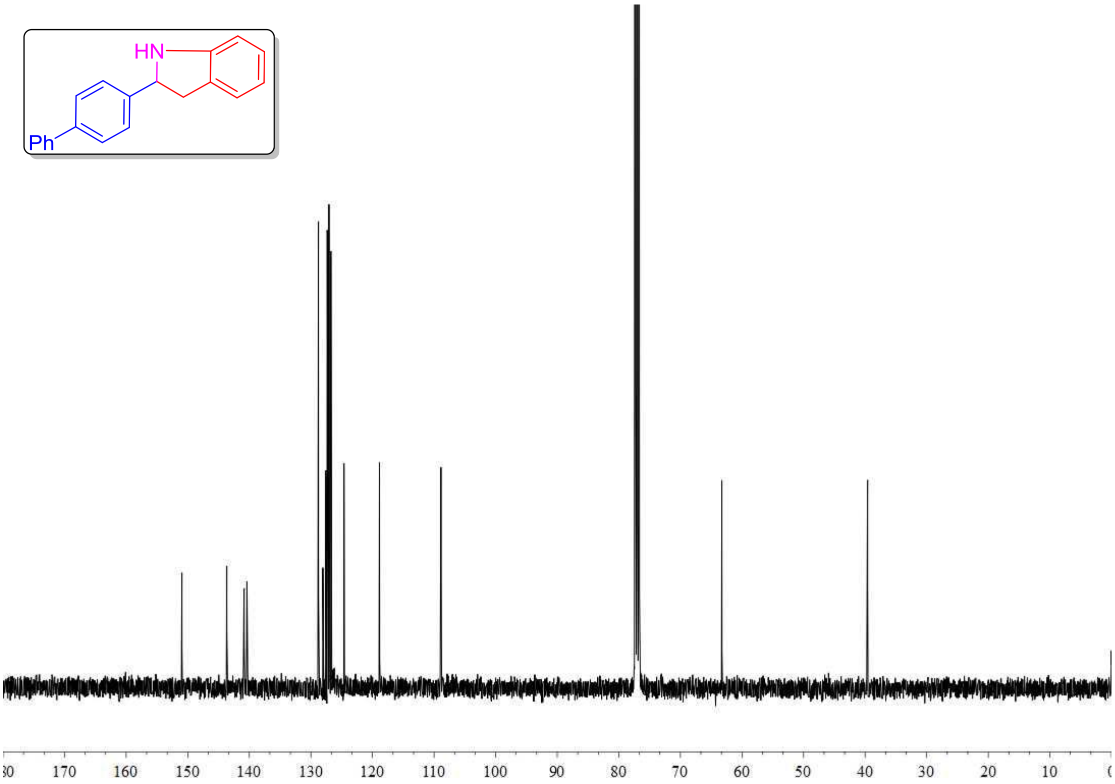

Supplementary Figure 87. ^{1}H NMR Spectrum of 5a (400 MHz, $CDCl_3$)

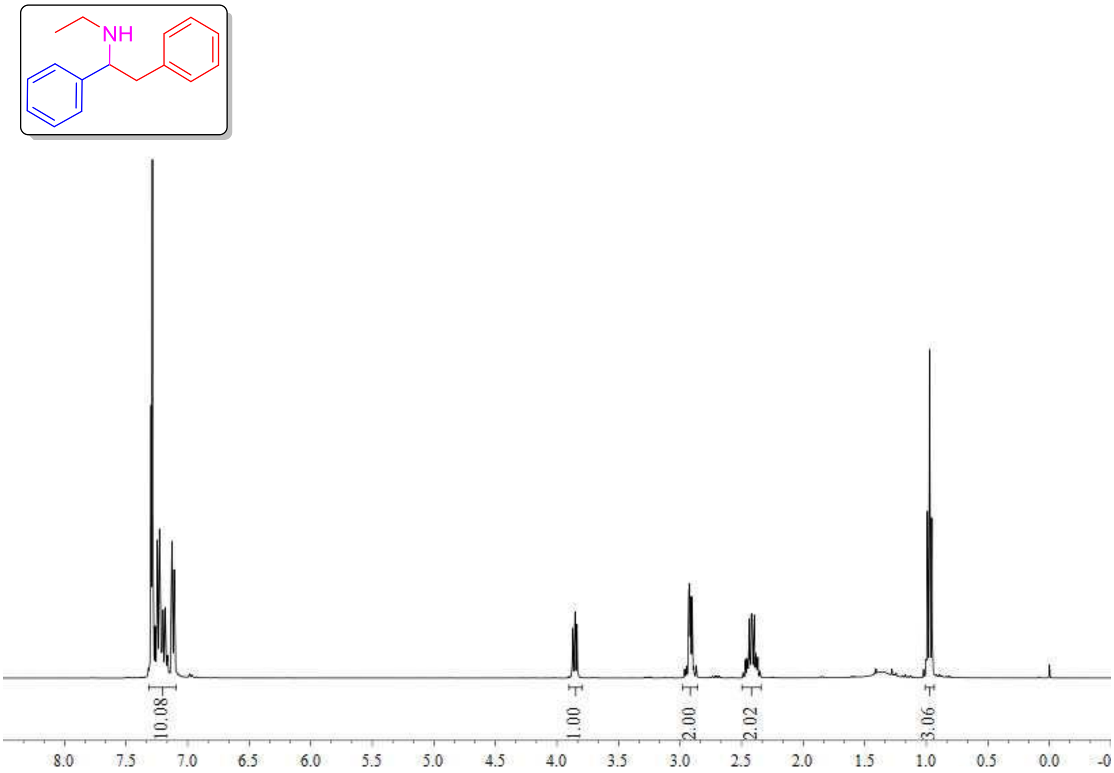

Supplementary Figure 88. ^{13}C NMR Spectrum of 5a (101 MHz, $CDCl_3$)

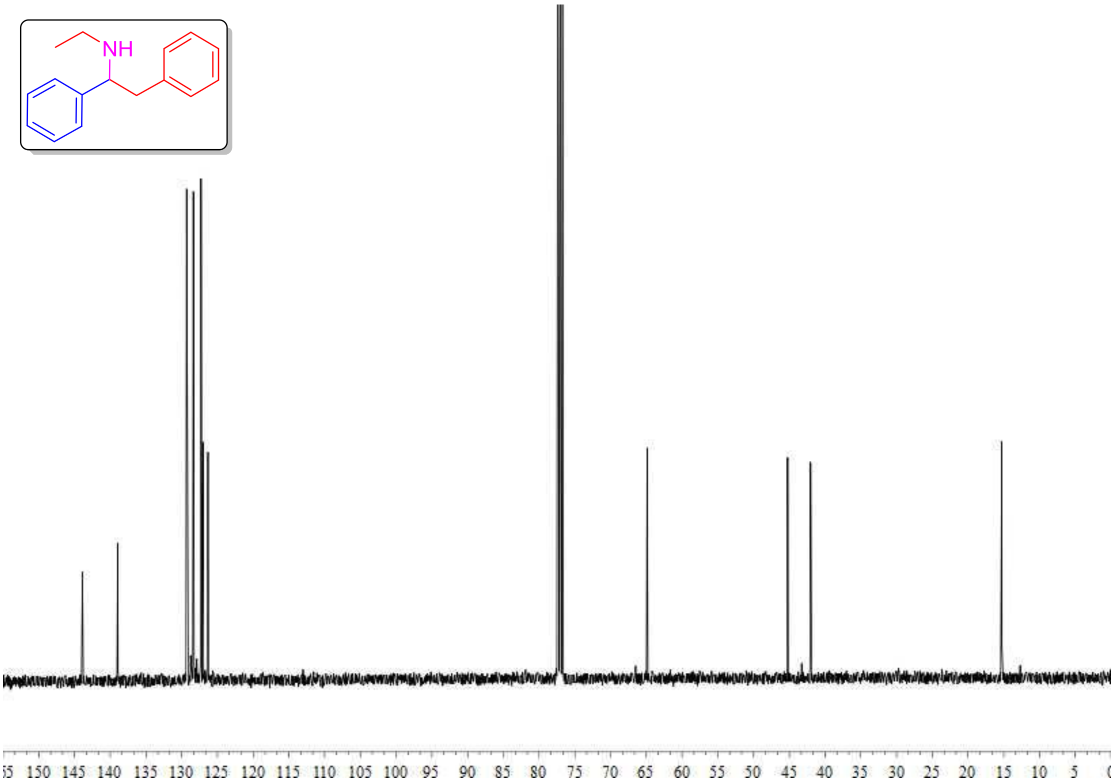

Supplementary Figure 89. ^{1}H **NMR Spectrum of 5b (400 MHz,** $CDCl_3$**)**

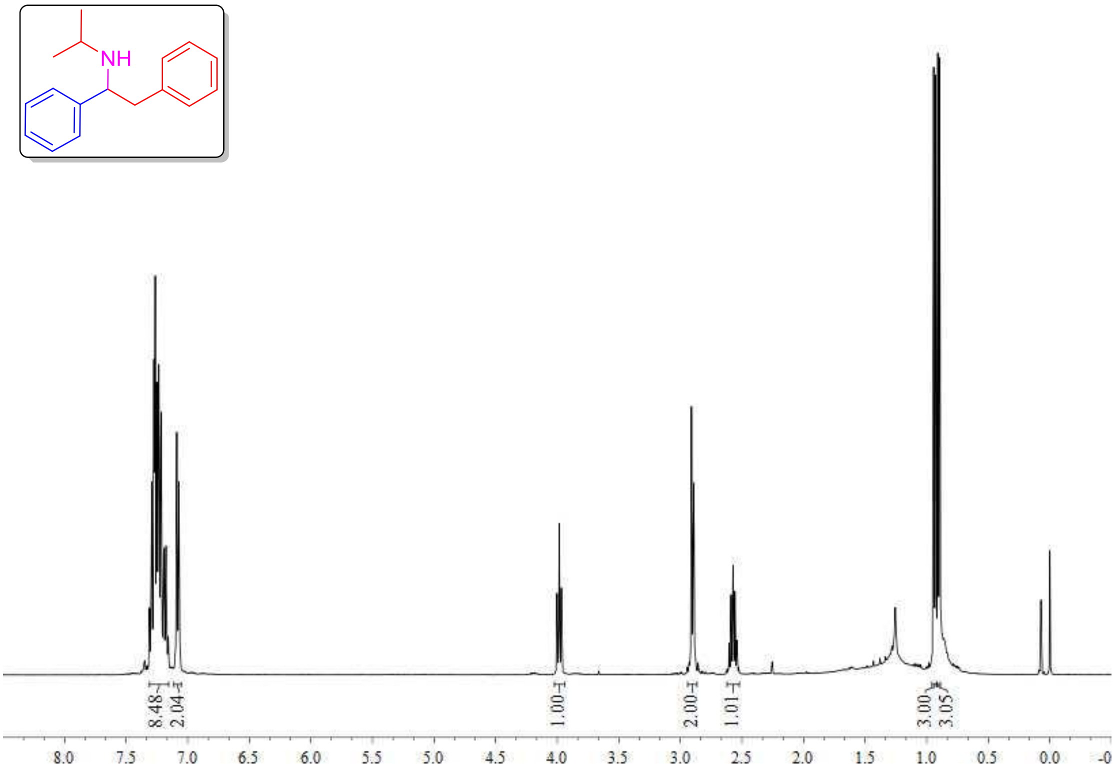

Supplementary Figure 90. ^{13}C **NMR Spectrum of 5b (101 MHz,** $CDCl_3$**)**

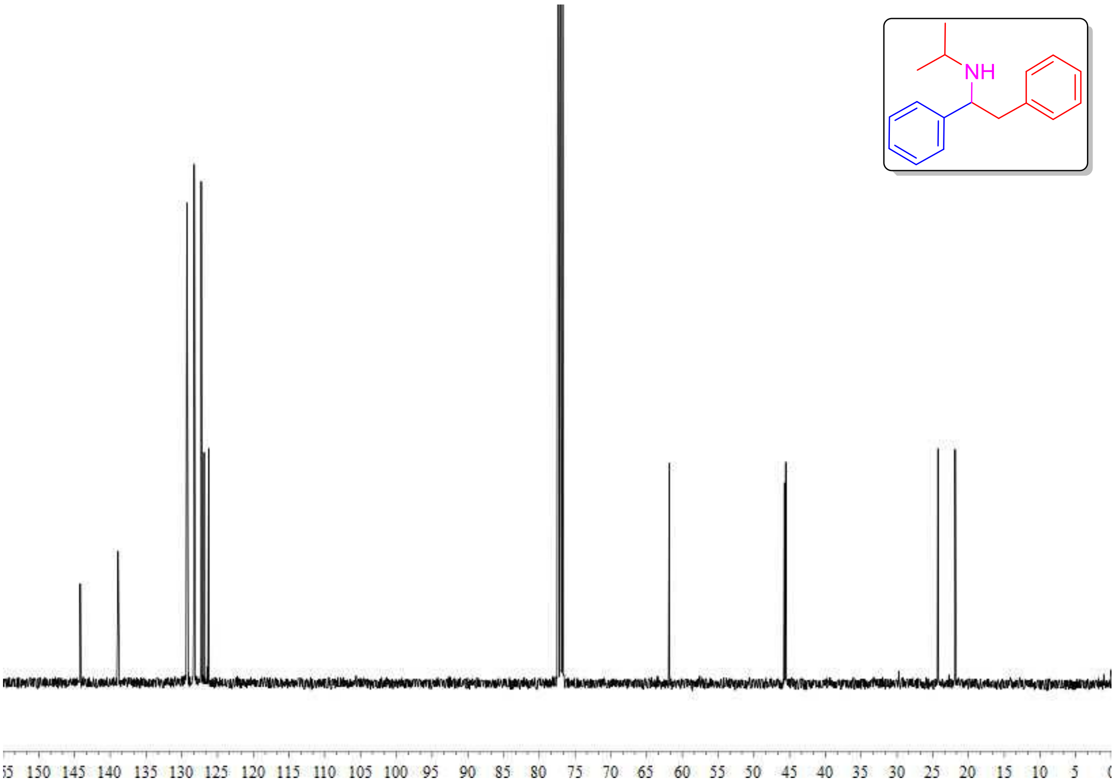

Supplementary Figure 91. ^{1}**H NMR Spectrum of 5c (400 MHz, CDCl$_3$)**

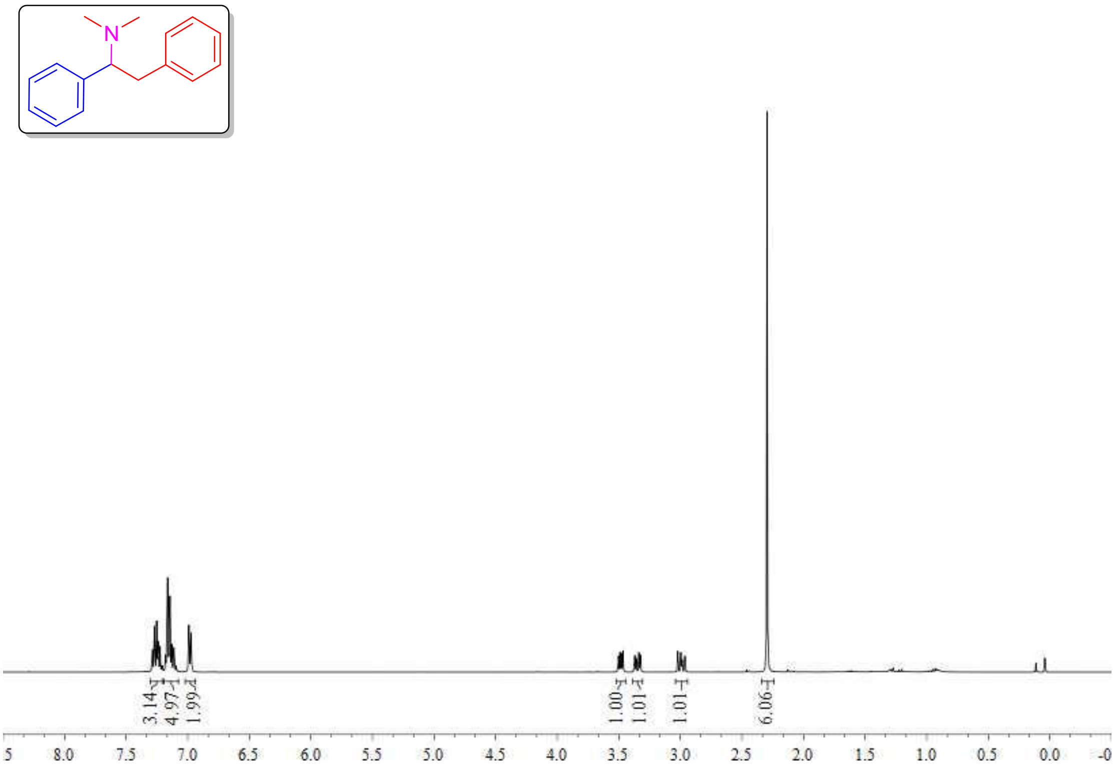

Supplementary Figure 92. ^{13}**C NMR Spectrum of 5c (101 MHz, CDCl$_3$)**

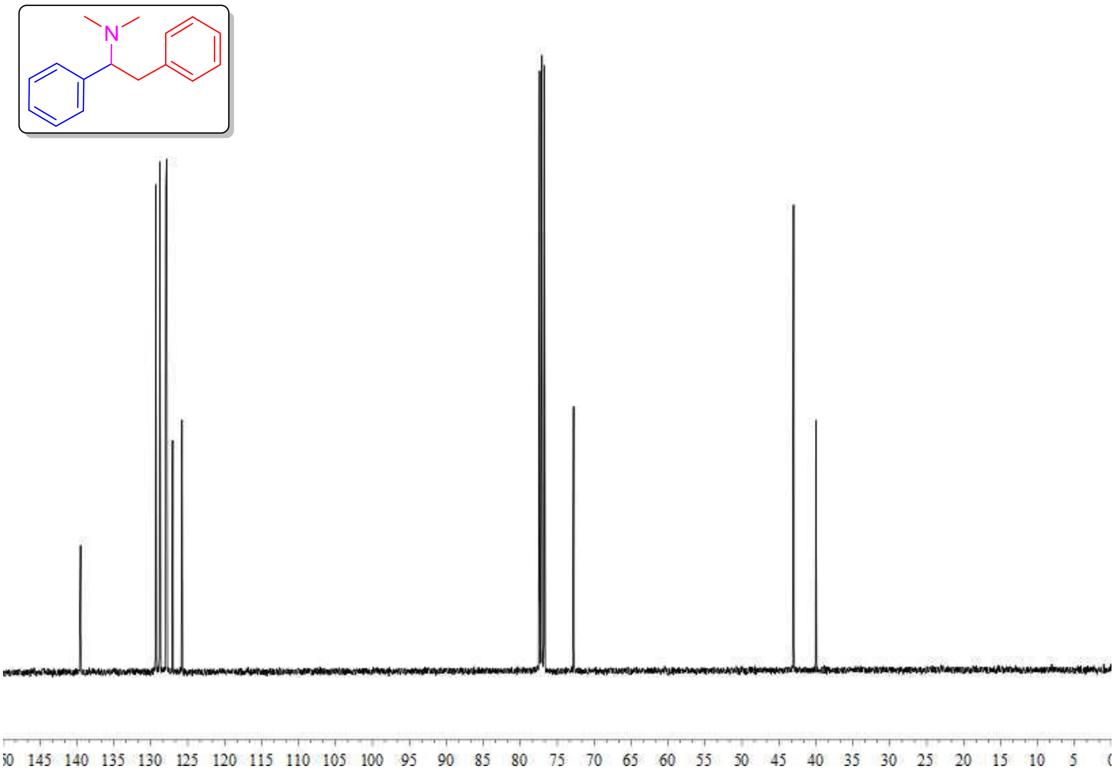

Supplementary Figure 93. ^{1}H NMR Spectrum of 5d (400 MHz, $CDCl_3$)

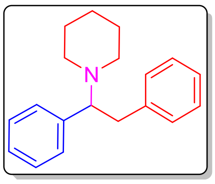

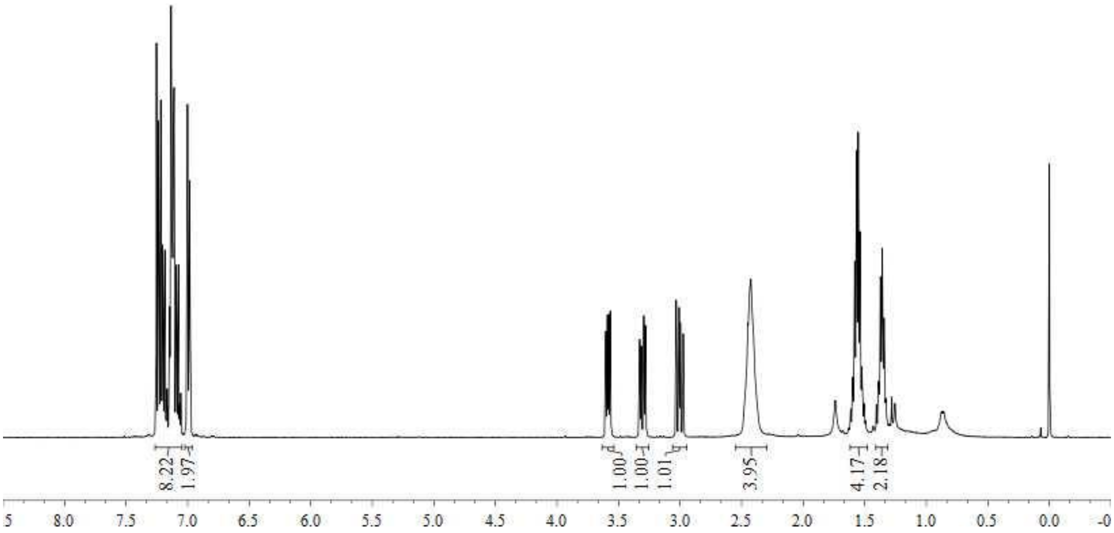

Supplementary Figure 94. ^{13}C NMR Spectrum of 5d (101 MHz, $CDCl_3$)

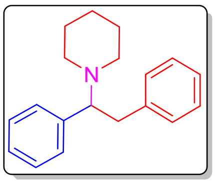

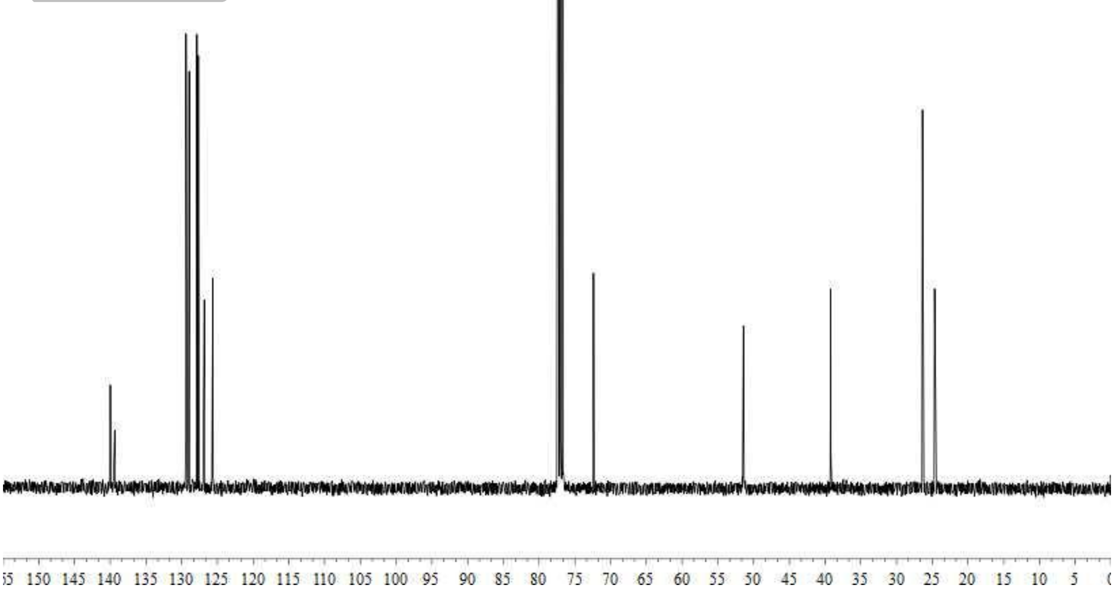

Supplementary Methods

All reactions were performed under an atmosphere of dry argon. Toluene was dried by heating with $AlLiH_4$ at reflux and distilled before use. Lithium bis(trimethylsilyl)amide (LiHMDS; Aldrich, 97%), Sodium bis(trimethylsilyl)amide (NaHMDS; Aldrich, 95%), Potassium bis(trimethylsilyl)amide (KHMDS; Aldrich, 95%), CsF (Aldrich, 99%), Cs_2CO_3 (Aldrich, 99%), CsCl (Alfa, 99.998%), CsOAc (Aldrich, 99.9%), Cs_2SO_4 (Alfa, 99%), $CsClO_4$ (Acros, 99%), $EtCO_2Cs$ (Acros, 95%), CsBr (Adamas-beta, 99.9%), CsI (Adamas-beta, 99%+), $CsOOCCF_3$ (Alfa, 98%+). Unless otherwise stated, reagents were commercially available and used as purchased without further purification. Chemicals were obtained from Sigma-Aldrich, Acros, Alfa Aesar, TCI China, or Adamas-beta.

The progress of the reactions was monitored by thin-layer chromatography using TLC plates and visualized by short-wave ultraviolet light or by treatment with ninhydrin. Flash chromatography was performed with Qingdao Haiyang flash silica gel (200–300 mesh). The NMR spectra were obtained using a Brüker 400 MHz Fourier-transform NMR spectrometer. Chemical shifts were reported in units of parts per million (ppm) downfield from tetramethylsilane (TMS), and all coupling constants were reported in hertz. Trace element/metal analysis was performed on an Thermo X series inductively coupled plasma (ICP) mass spectrometer calibrated against multi-element standard solutions. The infrared spectra were obtained with KBr plates by using a IS10 FT-IR Spectrometer (ThermoFisher Corporation). High resolution mass spectrometry (HRMS) data were obtained on a Waters LC-TOF mass spectrometer (Xevo G2-XS QTof) using electrospray ionization (ESI) in positive or negative mode. Melting points were measured using a SGW X-4 Melt-Temp apparatus and were uncorrected.

Supplement Table 1. ICP-MS metal analysis[a]

Element	NaHMDS (Aldrich 95%)	CsTFA (Alfa 98%+)	Reaction mixture
**Ti**	0.0748	0.0002	0.191
**Y**	0.0001	0.0139	0.0016
**Zr**	0.0047	0.0003	0.288
**Nb**	LOD	0.0045	0.0006
**Mo**	0.0005	0.0089	0.0068
**La**	LOD	0.0003	0.0049
**Ce**	0.0007	LOD	0.0106
**Pr**	LOD	0.0001	0.0003
**Nd**	0.0007	0.0001	0.0011
**Sm**	LOD	0.0002	0.0002
**Eu**	LOD	0.0002	0.0001
**Gd**	LOD	LOD	0.0003
**Tb**	LOD	0.0001	LOD
**Dy**	LOD	LOD	0.0002
**Ho**	LOD	0.0001	0.0001
**Er**	LOD	LOD	0.0002
**Tm**	LOD	0.0001	LOD
**Yb**	LOD	LOD	0.0003
**Lu**	LOD	0.0017	LOD
**Hf**	LOD	0.0001	0.0081

**Ta**	LOD	0.0006	0.0024
**W**	0.0026	0.0108	0.0034
**Li**	0.247	LOD	0.2124
**Be**	LOD	0.0260	0.0003
**Mg**	0.0769	0.1782	2.2577
**Al**	0.0204	0.0003	5.3074
**V**	0.0002	0.0104	0.0013
**Cr**	0.0061	0.0046	0.1203
**Mn**	0.729	0.318	0.3919
**Fe**	0.305	0.0008	0.9553
**Co**	0.0160	0.0033	0.0522
**Ni**	0.0558	0.0027	0.0635
**Cu**	0.0219	0.0507	0.0361
**Zn**	0.447	0.0146	1.3289
**Ga**	0.0020	LOD	0.0097
**As**	0.0077	0.0772	0.0086
**Sr**	0.0146	0.0001	0.0618
**Cd**	0.0007	0.0077	0.0008
**Sn**	0.0034	0.2648	0.0068
**Ba**	0.0034	0.0005	0.1330
**Tl**	LOD	0.0003	LOD
**Pb**	0.0038	0.0025	0.0084
**Bi**	LOD	0.0003	0.0010
**Sc**	0.0118	0.0927	0.0097
**Rb**	0.0005	0.0462	0.0432
**Mo**	0.0094	0.0868	0.0683
**Ag**	0.0713	0.0006	0.2849
**In**	LOD	10000	0.0003
**Cs**	0.4079	0.0001	3816
**Th**	LOD	0.0008	0.0030
**U**	0.0047	0.0005	0.0037
**Ge**	0.0008	LOD	0.0003
**Ru**	0.0014	LOD	LOD
**Rh**	LOD	0.0027	LOD
**Pd**	0.0697	LOD	0.0192
**Re**	LOD	0.0006	LOD
**Ir**	0.0050	LOD	0.0026
**Pt**	LOD	LOD	LOD
**Au**	LOD	27.30	0.0013
**K**	7.134	8.247	23.65
**Ca**	15.45	LOD	68.93
**Na**	10000	0.0002	10000
Amount overal	10025	10037	13921

Amount Na	10000	Amount Cs 10000	10000
Na(%)	99.75	Cs(%) 99.63	71.84

[a]Values in ppm relative to sodium or cesium.

ICP trace metal analysis was carried out using commercially available NaHMDS (Aldrich, 95%), CsTFA (Alfa, 98%+) and a standard reaction mixture. These data were displayed in Table 1. As a result of these quantitative analyses, there were no appreciable transition metal contaminants. Furthermore, the reactions have been conducted in two countries with different reagents from different vendors and the results are completely reproducible.

Synthesis of 1,2-diarylethylamine

General Procedure A: To an oven-dried microwave vial equipped with a stir bar under argon atmosphere inside a glove box was added $NaN(SiMe_3)_2$ (73.2 mg, 0.40 mmol), cesium trifluoroacetate (CsTFA) (17.2 mg, 0.07 mmol) and toluene (2 mL). Then the corresponding aldehyde (0.20 mmol) was added via syringe. The microwave vial was sealed with a cap and removed from the glove box. The reaction mixture was heated to 110 ℃ in an oil bath and stirred for 12 h. The sealed vial was cooled to room temperature, opened to air, and then 5 drops of water was added. The reaction mixture was passed through a short pad of silica, washed with an additional 6 mL of ethyl acetate (3 × 2 mL), and the combined solutions were concentrated *in vacuo*. The crude material was loaded onto a column of silica gel for purification of the amine.

General Procedure B: To an oven-dried microwave vial equipped with a stir bar under an argon atmosphere inside a glove box was added $NaN(SiMe_3)_2$ (73.2 mg, 0.40 mmol), CsTFA (17.2 mg, 0.07 mmol), the toluene derivative (1 mL) and cyclohexane (1 mL). Then the corresponding aldehyde (0.20 mmol) was added via syringe. The microwave vial was sealed with a cap and removed from the glove box. The reaction mixture was heated to 110 ℃ in an oil bath and stirred for 12 h. The sealed vial was cooled to room temperature, opened to air, and then 5 drops of water was added. The reaction mixture was passed through a short pad of silica, washed with additional 6 mL of ethyl acetate (3 × 2 mL), and the combined solutions were concentrated *in vacuo*. The crude material was loaded onto a column of silica gel for purification of the amine.

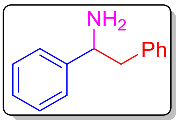

1,2-Diphenylethan-1-amine (3aa) The reaction was performed following the General Procedure A with benzaldehyde **(1a)** (20 μL, 0.20 mmol), $NaN(SiMe_3)_2$ (73.2 mg, 0.40 mmol), CsTFA (17.2 mg, 0.07 mmol) and toluene **(2a)** (2 mL). The crude product was purified by chromatography on silica gel (eluted with Petroleum ether:EtOAc:Et_3N = 100:20:5) to give the desired product (36.2 mg, 92% yield) as a yellow oil. R_f = 0.49 (Petroleum ether:EtOAc:Et_3N = 100:20:5). ^{1}H NMR (400 MHz, $CDCl_3$) δ: 7.37 – 7.30 (m, 4H), 7.29 – 7.20 (m, 4H), 7.19 – 7.16 (m, 2H), 4.19 (dd, J = 8.9, 4.9, Hz, 1H), 3.01 (dd, J = 13.3, 5.0, Hz, 1H), 2.82 (dd, J = 8.9, 13.3, Hz, 1H), 1.55 (brs, 2H) ppm. $^{13}C\{^1H\}$ NMR (101 MHz, $CDCl_3$) δ: 145.6, 139.1, 129.4, 128.5, 128.4, 127.1, 126.46, 126.41, 57.6, 46.5 ppm. The spectroscopic data for this product match the literature.[1]

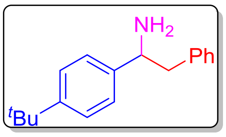

1-(4-(*tert*-Butyl)phenyl)-2-phenylethan-1-amine (3ba) The reaction was performed following the General Procedure A with 4-(*tert*-butyl)benzaldehyde **(1b)** (33.2 μL, 0.20 mmol), $NaN(SiMe_3)_2$ (73.2 mg, 0.40 mmol), CsTFA (17.2 mg, 0.07 mmol) and toluene **(2a)** (2 mL). The crude product was purified by chromatography on silica gel (eluted with Petroleum ether:EtOAc:Et_3N = 100:20:5) to give the desired product (44.5 mg, 88% yield) as a white solid. Mp 49.5–51.5 °C. R_f = 0.30 (Petroleum ether:EtOAc:Et_3N = 100:20:5). ^{1}H NMR (400 MHz, $CDCl_3$) δ: 7.38 – 7.36 (m, 2H), 7.33 – 7.28 (m, 4H), 7.25 – 7.21 (m, 3H), 4.17 (dd, J = 9.4, 4.4 Hz, 1H), 3.03 (dd, J = 13.3, 4.4 Hz, 1H), 2.78 (dd, J = 13.3, 9.3 Hz, 1H), 1.50 (brs, 2H), 1.32 (s, 9H) ppm.

$^{13}C\{^1H\}$ NMR (101 MHz, $CDCl_3$) δ: 150.0, 142.8, 139.5, 129.4, 128.5, 126.5, 126.2, 125.4, 57.2, 46.6, 34.6, 31.5 ppm. IR (neat): 3375, 3314, 2962, 1603, 1584, 1509, 1494, 1454, 825, 748, 701 cm^{-1}. HRMS: calcd for $C_{18}H_{23}N$ $[M+H]^+$ 254.1864, found 254.1869.

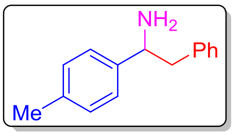

2-Phenyl-1-(*p*-tolyl)ethan-1-amine (3ca) The reaction was performed following the General Procedure A with 4-methylbenzaldehyde **(1c)** (23.1 µL, 0.20 mmol), $NaN(SiMe_3)_2$ (73.2 mg, 0.40 mmol), CsTFA (17.2 mg, 0.07 mmol) and toluene **(2a)** (2 mL). The crude product was purified by chromatography on silica gel (eluted with Petroleum ether:EtOAc:Et_3N = 100:20:5) to give the desired product (29.5 mg, 70% yield) as a yellow oil. R_f = 0.26 (Petroleum ether:EtOAc:Et_3N = 100:20:5). ^1H NMR (400 MHz, $CDCl_3$) δ: 7.34 – 7.25 (m, 5H), 7.23 – 7.17 (m, 4H), 4.20 (dd, J = 9.0, 4.9 Hz, 1H), 3.03 (dd, J = 13.3, 4.8 Hz, 1H), 2.82 (dd, J = 13.4, 9.0 Hz, 1H), 2.38 (s, 3H), 1.59 (brs, 2H) ppm. $^{13}C\{^1H\}$ NMR (101 MHz, $CDCl_3$) δ: 142.7. 139.3, 136.8, 129.5, 129.2, 128.6, 126.49, 126.45, 57.36, 46.55, 21.22 ppm. The spectroscopic data for this product match the literature data.[2]

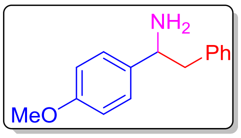

1-(4-Methoxyphenyl)-2-phenylethan-1-amine (3da) The reaction was performed following the General Procedure A with 4-methoxybenzaldehyde **(1d)** (22.3 µL, 0.20 mmol), $NaN(SiMe_3)_2$ (73.2 mg, 0.40 mmol), CsTFA (17.2 mg, 0.07 mmol) and toluene **(2a)** (2 mL). The crude product was purified by chromatography on silica gel (eluted with Petroleum ether:EtOAc:Et_3N = 100:20:5) to give the desired product (39.0 mg, 86% yield) as a yellow oil. R_f = 0.28 (Petroleum ether:EtOAc:Et_3N = 100:20:5). ^1H NMR (400 MHz, $CDCl_3$) δ: 7.30 – 7.24 (m, 4H), 7.22 – 7.15 (m, 3H), 6.87 – 6.85 (m, 2H), 4.14 (dd, J = 8.8, 5.0 Hz, 1H), 3.79 (s, 3H), 2.97 (dd, J = 13.2, 5.0 Hz, 1H), 2.80 (dd, J = 13.3, 8.8 Hz, 1H) 1.59 (brs, 2H) ppm. $^{13}C\{^1H\}$ NMR (101 MHz, $CDCl_3$) δ: 158.7, 139.3, 137.9, 129.4, 128.5, 127.6, 126.4, 113.8, 57.0, 55.4, 46.7 ppm. The spectroscopic data for this product match the literature data.[2]

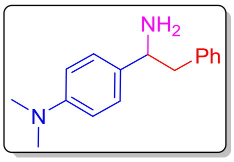

4-(1-Amino-2-phenylethyl)-*N,N*-dimethylaniline (3ea) The reaction was performed following the General Procedure A with 4-(dimethylamino)benzaldehyde **(1e)** (29.8 mg, 0.20 mmol), $NaN(SiMe_3)_2$ (73.2 mg, 0.40 mmol), CsTFA (17.2 mg, 0.07 mmol) and toluene **(2a)** (2 mL). The crude product was purified by chromatography on silica gel (eluted with Petroleum ether:EtOAc:Et_3N = 100:50:7) to give the desired product (37.9 mg, 79% yield) as a yellow oil. R_f = 0.23 (Petroleum ether:EtOAc:Et_3N = 100:50:7). ^1H NMR (400 MHz, $CDCl_3$) δ: 7.31 – 7.18 (m, 7H), 6.74 – 6.71 (m, 2H), 4.11 (dd, J = 9.0, 4.8 Hz, 1H), 2.99 (dd, J = 13.3, 4.8 Hz, 1H), 2.94 (s, 6H), 2.80 (dd, J = 13.3, 9.0 Hz, 1H), 1.63 (brs, 2H) ppm. $^{13}C\{^1H\}$ NMR (101 MHz, $CDCl_3$) δ: 149.7, 139.4, 133.5, 129.2, 128.2, 126.9, 126.0, 112.5, 56.7, 46.3, 40.6 ppm. The spectroscopic data for this product match the literature data.[2]

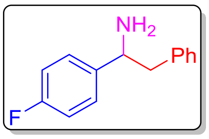

1-(4-Fuorophenyl)-2-phenylethan-1-amine (3fa) The reaction was performed following the General Procedure A with 4-fluorobenzaldehyde **(1f)** (21.4 µL, 0.20 mmol), $NaN(SiMe_3)_2$ (73.2 mg, 0.40 mmol), CsTFA (17.2 mg, 0.07 mmol) and toluene **(2a)** (2 mL). The crude product was purified by chromatography on silica gel (eluted with Petroleum ether:EtOAc:Et_3N = 100:20:5) to give the desired product (38.7 mg, 90% yield) as a brown oil. R_f = 0.62 (Petroleum ether:EtOAc:Et_3N = 100:20:5). ^1H NMR (400 MHz, $CDCl_3$) δ: 7.32 – 7.20 (m, 5H), 7.15 – 7.13 (m, 2H), 7.02 – 6.98 (m, 2H), 4.18 (dd, J = 8.6, 5.1 Hz, 1H), 2.95 (dd, J = 13.2, 5.2 Hz, 1H), 2.80 (dd, J = 13.2, 8.6 Hz, 1H) 1.48 (brs, 2H) ppm. $^{13}C\{^1H\}$ NMR (101 MHz, $CDCl_3$) δ: 162.0 (d, J^1_{C-F} = 245.6 Hz), 141.35 (d, J^4_{C-F} = 3.1 Hz), 138.9, 129.4, 128.6, 128.1 (d, J^3_{C-F} = 8.0 Hz), 126.6, 115.2 (d, J^2_{C-F} =

21.3 Hz), 57.03, 46.72 ppm. The spectroscopic data for this product match the literature data.[2]

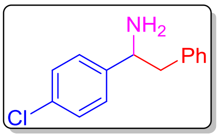

1-(4-Chlorophenyl)-2-phenylethan-1-amine (3ga) The reaction was performed following the General Procedure A with 4-chlorobenzaldehyde **(1g)** (23.5 μL, 0.20 mmol), $NaN(SiMe_3)_2$ (73.2 mg, 0.40 mmol), CsTFA (17.2 mg, 0.07 mmol) and toluene **(2a)** (2 mL). The crude product was purified by chromatography on silica gel (eluted with Petroleum ether:EtOAc:Et_3N = 100:20:5) to give the desired product (33.4 mg, 72% yield) as a yellow oil. R_f = 0.32 (Petroleum ether:EtOAc:Et_3N = 100:20:5). ^{1}H NMR (400 MHz, $CDCl_3$) δ: 7.30 – 7.20 (m, 7H), 7.15 – 7.13 (m, 2H), 4.17 (dd, J = 8.6, 5.2 Hz, 1H), 2.95 (dd, J = 13.3, 4.8 Hz, 1H), 2.79 (dd, J = 13.3, 8.6 Hz, 1H) 1.51 (brs, 2H) ppm. ^{13}C{^{1}H} NMR (101 MHz, $CDCl_3$) δ: 144.2, 138.7, 132.7, 129.9, 129.4, 128.6, 128.0, 126.6, 57.1, 46.6 ppm. The spectroscopic data for this product match the literature data.[2]

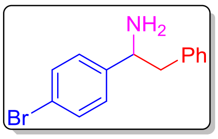

1-(4-Bromophenyl)-2-phenylethan-1-amine (3ha) The reaction was performed following the General Procedure A with 4-bromobenzaldehyde **(1h)** (37.0 mg, 0.20 mmol), $NaN(SiMe_3)_2$ (73.2 mg, 0.40 mmol), CsTFA (17.2 mg, 0.07 mmol) and toluene **(2a)** (2 mL). The crude product was purified by chromatography on silica gel (eluted with Petroleum ether:EtOAc:Et_3N = 100:20:5) to give the desired product (45.9 mg, 83% yield) as a yellow oil. R_f = 0.59 (Petroleum ether:EtOAc:Et_3N = 100:20:5). ^{1}H NMR (400 MHz, $CDCl_3$) δ: 7.45 – 7.42 (m, 2H), 7.31 – 7.20 (m, 5H), 7.15 – 7.13 (m, 2H), 4.17 (dd, J = 8.6, 5.1 Hz, 1H), 2.79 (dd, J = 13.3, 5.2 Hz, 1H), 2.79 (dd, J = 13.3, 8.6 Hz, 1H) 1.45 (brs, 2H) ppm. ^{13}C{^{1}H} NMR (101 MHz, $CDCl_3$) δ: 144.7, 138.7, 131.5, 129.4, 128.6, 128.4, 126.6, 120.8, 57.1, 46.5 ppm. The spectroscopic data for this product match the literature data.[3]

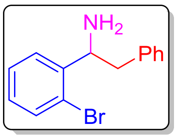

1-(2-Bromophenyl)-2-phenylethan-1-amine (3ia) The reaction was performed following the General Procedure A with 2-bromobenzaldehyde **(1i)** (23.3 μL, 0.20 mmol), $NaN(SiMe_3)_2$ (73.2 mg, 0.40 mmol), CsTFA (17.2 mg, 0.07 mmol) and toluene **(2a)** (2 mL). The crude product was purified by chromatography on silica gel (eluted with Petroleum ether:EtOAc:Et_3N = 100:20:5) to give the desired product (45.8 mg, 83% yield) as a brown oil. R_f = 0.14 (Petroleum ether:EtOAc:Et_3N = 100:20:5). ^{1}H NMR (400 MHz, $CDCl_3$) δ: 7.61 – 7.54 (m, 2H), 7.35 – 7.24 (m, 6H), 7.14 – 7.10 (m, 1H), 4.62 (dd, J = 9.6, 3.7 Hz, 1H), 3.15 (dd, J = 13.5, 3.7 Hz, 1H), 2.60 (dd, J = 13.4, 9.6 Hz, 1H), 1.50 (brs, 2H) ppm. ^{13}C{^{1}H} NMR (101 MHz, $CDCl_3$) δ: 144.4, 139.1, 132.9, 129.5, 128.6, 128.5, 127.8, 127.6, 126.7, 123.4, 56.0, 44.6 ppm. IR (neat): 3376, 3307, 3026, 1602, 1566, 1494, 1465, 1453, 754, 700 cm^{-1}. HRMS: calcd for $C_{14}H_{14}BrN$ $[M+H]^+$ 277.0289, found 277.0289.

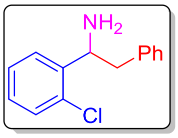

1-(2-Chlorophenyl)-2-phenylethan-1-amine (3ja) The reaction was performed following the General Procedure A with 2-chlorobenzaldehyde **(1j)** (22.5 μL, 0.20 mmol), $NaN(SiMe_3)_2$ (73.2 mg, 0.40 mmol), CsTFA (17.2 mg, 0.07 mmol) and toluene **(2a)** (2 mL). The crude product was purified by chromatography on silica gel (eluted with Petroleum ether:EtOAc:Et_3N = 100:20:5) to give the desired product (42.6 mg, 92% yield) as a white solid. R_f = 0.14 (Petroleum ether:EtOAc:Et_3N = 100:20:5). ^{1}H NMR (400 MHz, $CDCl_3$) δ: 7.58 – 7.56 (m, 1H), 7.37 – 7.17 (m, 8H), 4.66 (dd, J = 9.3, 3.8 Hz, 1H), 3.14 (dd, J = 13.7, 3.9 Hz, 1H), 2.65 (dd, J = 13.4, 9.3 Hz, 1H), 1.67 (brs, 2H) ppm. ^{13}C{^{1}H} NMR (101 MHz, $CDCl_3$) δ: 142.9, 139.1, 132.8, 129.7, 129.5, 128.6, 128.1, 127.4, 127.2, 126.6, 53.6, 44.5 ppm. The spectroscopic data for this product match the literature data.[4]

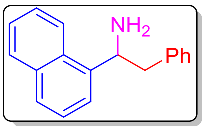

1-(Naphthalen-1-yl)-2-phenylethan-1-amine (3ka) The reaction was performed following the General Procedure A with 1-naphthaldehyde **(1k)** (27.2 μL, 0.20

mmol), $NaN(SiMe_3)_2$ (73.2 mg, 0.40 mmol), CsTFA (17.2 mg, 0.07 mmol) and toluene **(2a)** (2 mL). The crude product was purified by chromatography on silica gel (eluted with Petroleum ether:EtOAc:Et_3N = 100:20:5) to give the desired product (44.4 mg, 90% yield) as a yellow oil. R_f = 0.81 (Petroleum ether:EtOAc:Et_3N = 100:20:5). ^1H NMR (400 MHz, $CDCl_3$) δ: 8.22 (d, J = 8.4 Hz, 1H), 7.87 (d, J = 7.9 Hz, 1H), 7.75 (d, J = 8.2 Hz, 1H), 7.68 (d, J = 7.1 Hz, 1H), 7.56 – 7.45 (m, 3H), 7.33 – 7.20 (m, 5H), 5.03 (dd, J = 9.4, 3.7 Hz, 1H), 3.26 (dd, J = 13.6, 3.7 Hz, 1H), 2.84 (dd, J = 13.5, 9.4 Hz, 1H), 1.64 (brs, 2H) ppm. $^{13}C\{^1H\}$ NMR (101 MHz, $CDCl_3$) δ: 141.3, 139.4, 134.0, 130.8, 129.4, 129.2, 128.6, 127.6, 126.6, 126.1, 125.7, 125.5, 122.8, 52.6, 45.4 ppm. One resonance was not observed due to overlapping resonances. IR (neat): 3565, 3445, 3028, 1640, 1509, 1495, 1453, 1395, 776, 699cm^{-1}. HRMS: calcd for $C_{18}H_{17}N$ $[M+H]^+$ 248.1395, found 248.1398.

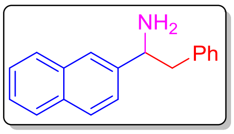

1-(Naphthalen-2-yl)-2-phenylethan-1-amine (3la) The reaction was performed following the General Procedure A with 2-naphthaldehyde **(1l)** (31.2 mg, 0.20 mmol), $NaN(SiMe_3)_2$ (73.2 mg, 0.40 mmol), CsTFA (17.2 mg, 0.07 mmol) and toluene **(2a)** (2 mL). The crude product was purified by chromatography on silica gel (eluted with Petroleum ether:EtOAc:Et_3N = 100:20:5) to give the desired product (43.4 mg, 88% yield) as a white solid. Mp 72–74 °C. R_f = 0.46 (Petroleum ether:EtOAc:Et_3N = 100:20:5). ^1H NMR (400 MHz, $CDCl_3$) δ: 7.83 – 7.79 (m, 4H), 7.52 – 7.43 (m, 3H), 7.31 – 7.19 (m, 5H), 4.36 (dd, J = 8.8, 4.9 Hz, 1H), 3.10 (dd, J = 13.4, 4.9 Hz, 1H), 2.89 (dd, J = 13.4, 8.9 Hz, 1H), 1.59 (brs, 2H) ppm. $^{13}C\{^1H\}$ NMR (101 MHz, $CDCl_3$) δ: 143.1, 139.1, 133.5, 132.9, 129.5, 128.6, 128.2, 128.0, 127.8, 126.6, 126.1, 125.7, 125.1, 124.9, 57.7, 46.5 ppm. IR (neat): 3369, 3304, 3025, 1601, 1583, 1438, 1375, 748, 699 cm^{-1}. HRMS: calcd for $C_{18}H_{17}N$ $[M+H]^+$ 248.1395, found 248.1398.

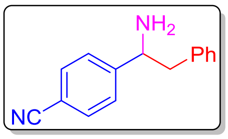

4-(1-Amino-2-phenylethyl)benzonitrile (3ma) The reaction was performed following the General Procedure A with 4-formylbenzonitrile **(1m)** (26.2 mg, 0.20 mmol), $NaN(SiMe_3)_2$ (73.2 mg, 0.40 mmol), CsTFA (17.2 mg, 0.07 mmol) and toluene **(2a)** (2 mL). The crude product was purified by flash chromatography on silica gel (eluted with Petroleum ether:EtOAc:Et_3N = 100:50:7) to give the desired product (31.1 mg, 70% yield) as a yellow oil. R_f = 0.54 (Petroleum ether:EtOAc:Et_3N = 100:50:7). ^1H NMR (400 MHz, $CDCl_3$) δ: 7.62 – 7.60 (m, 2H), 7.46 – 7.44 (m, 2H), 7.31 – 7.22 (m, 3H), 7.13 – 7.11 (m, 2H), 4.27 (dd, J = 8.5, 5.3 Hz, 1H), 2.96 (dd, J = 13.3, 5.3 Hz, 1H), 2.81 (dd, J = 13.4, 8.5 Hz, 1H), 1.60 (brs, 2H), ppm. $^{13}C\{^1H\}$ NMR (101 MHz, $CDCl_3$) δ: 151.0, 138.1, 132.4, 129.4, 128.7, 127.5, 126.9, 119.1, 111.0, 57.5, 46.4 ppm. IR (neat): 3477, 3440, 3006, 1655, 1561, 1556, 1505, 1458, 764, 750 cm^{-1}. HRMS: calcd for $C_{15}H_{14}N_2$ $[M+H]^+$ 223.2930, found 223.2932.

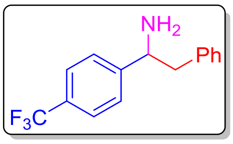

2-Phenyl-1-(4-(trifluoromethyl)phenyl)ethan-1-amine (3na) The reaction was performed following the General Procedure A with 4-(trifluoromethyl)benzaldehyde **(1n)** (27.3 μL, 0.20 mmol), $NaN(SiMe_3)_2$ (73.2 mg, 0.40 mmol), CsTFA (17.2 mg, 0.07 mmol) and toluene **(2a)** (2 mL). The crude product was purified by chromatography on silica gel (eluted with Petroleum ether:EtOAc:Et_3N = 100:20:5) to give the desired product (46.6 mg, 88% yield) as a yellow oil. R_f = 0.83 (Petroleum ether:EtOAc:Et_3N = 100:20:5). ^1H NMR (400 MHz, $CDCl_3$) δ: 7.59 – 7.56 (m, 2H), 7.48 – 7.46 (m, 2H), 7.31 – 7.21 (m, 3H), 7.16 – 7.14 (m, 2H), 4.26 (dd, J = 8.7, 5.0 Hz, 1H), 2.99 (dd, J = 18.4, 5.0 Hz, 1H), 2.81 (dd, J = 13.3, 8.7 Hz, 1H), 1.48 (brs, 2H) ppm. $^{13}C\{^1H\}$ NMR (101 MHz, $CDCl_3$) δ: 149.7, 138.5, 129.44, 129.42 (q, $J^2_{C\text{-}F}$ = 32.4 Hz), 128.7, 127.0, 126.8, 125.5(q, $J^3_{C\text{-}F}$ = 3.7 Hz), 123.9(q, $J^1_{C\text{-}F}$ = 272.9 Hz), 57.4, 46.5 ppm. The spectroscopic data for this product match the literature data.[2]

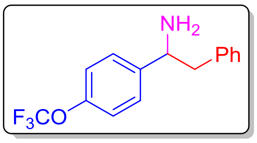

2-Phenyl-1-(4-(trifluoromethoxy)phenyl)ethan-1-amine (3oa) The reaction was performed following the General Procedure A with 4-(trifluoromethoxy)benzaldehyde **(1o)** (28.6 mg, 0.20 mmol), $NaN(SiMe_3)_2$ (73.2 mg, 0.40 mmol), CsTFA (17.2 mg, 0.07 mmol) and toluene (**2a**) (2 mL). The crude product was purified by chromatography on silica gel (eluted with Petroleum ether:EtOAc:Et_3N = 100:20:7) to give the desired product (50.6 mg, 90% yield) as a yellow oil. R_f = 0.28 (Petroleum ether:EtOAc:Et_3N = 100:20:7). ^{1}H NMR (400 MHz, $CDCl_3$) δ: 7.39 – 7.36 (m, 2H), 7.31 – 7.21 (m, 3H), 7.18 – 7.14 (m, 4H), 4.22 (dd, *J* = 8.8, 5.0 Hz, 1H), 2.97 (dd, *J* = 13.3, 5.0 Hz, 1H), 2.80 (dd, *J* = 13.3, 8.8 Hz, 1H), 1.51 (brs, 2H) ppm. $^{13}C\{^1H\}$ NMR (101 MHz, $CDCl_3$) δ: 148.3 (q, $J^2_{C\text{-}F}$ = 1.9 Hz), 144.4, 138.7, 129.4, 128.6, 127.9, 126.7, 121.0, 120.6 (q, $J^1_{C\text{-}F}$ = 257.8 Hz), 57.1, 46.7 ppm. IR (neat): 3376, 3311, 3029, 1603, 1508, 1496, 1454, 1262, 1223, 1164, 743, 700 cm^{-1}. HRMS: calcd for $C_{15}H_{14}F_3NO$ $[M+H]^+$ 282.1061, found 282.1066.

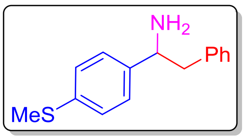

1-(4-(Methylthio)phenyl)-2-phenylethan-1-amine (3pa) The reaction was performed following the General Procedure A with 4-(methylthio)benzaldehyde **(1p)** (26.3 μL 0.20 mmol), $NaN(SiMe_3)_2$ (73.2 mg, 0.40 mmol), CsTFA (17.2 mg, 0.07 mmol) and toluene **(2a)** (2 mL). The crude product was purified by chromatography on silica gel (eluted with Petroleum ether:EtOAc:Et_3N = 100:50:7) to give the desired product (42.8 mg, 88% yield) as a white solid. Mp 62–64 °C. R_f = 0.35 (Petroleum ether:EtOAc:Et_3N = 100:50:7). ^{1}H NMR (400 MHz, $CDCl_3$) δ: 7.31 – 7.20 (m, 7H), 7.18 – 7.15 (m, 2H), 4.16 (dd, *J* = 8.8, 5.0 Hz, 1H), 2.98 (dd, *J* = 13.3, 5.0 Hz, 1H), 2.80 (dd, *J* = 13.3, 8.7 Hz, 1H), 2.48 (s, 3H), 1.65 (brs, 2H) ppm. $^{13}C\{^1H\}$ NMR (101 MHz, $CDCl_3$) δ: 142.7, 139.0, 137.0, 129.5, 128.6, 127.1, 126.9, 126.5, 57.2, 46.5, 16.2 ppm. IR (neat): 3438, 3373, 2919, 1598, 1561, 1493, 1452, 1091, 813, 744, 700 cm^{-1}. HRMS: calcd for $C_{15}H_{17}NS$ $[M+H]^+$ 244.1115, found 244.1113.

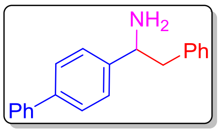

1-([1,1'-Biphenyl]-4-yl)-2-phenylethan-1-amine (3qa) The reaction was performed following the General Procedure A with [1,1'-biphenyl]-4-carbaldehyde **(1q)** (36.4 mg 0.20 mmol), $NaN(SiMe_3)_2$ (73.2 mg, 0.40 mmol), CsTFA (17.2 mg, 0.07 mmol) and toluene (**2a**) (2 mL). The crude product was purified by chromatography on silica gel (eluted with Petroleum ether:EtOAc:Et_3N = 100:20:5) to give the desired product (49.1 mg, 90% yield) as a white solid. Mp 65–67 °C. R_f = 0.39 (Petroleum ether:EtOAc:Et_3N = 100:20:5). ^{1}H NMR (400 MHz, $CDCl_3$) δ: 7.61 – 7.56 (m, 4H), 7.46 – 7.42 (m, 4H), 7.36 – 7.29 (m, 3H), 7.26 – 7.20 (m, 3H), 4.25 (dd, *J* = 9.0, 4.8 Hz, 1H), 3.06 (dd, *J* = 13.3, 4.8 Hz, 1H), 2.88 (dd, *J* = 13.4, 9.0 Hz, 1H), 1.57 (brs, 2H) ppm. $^{13}C\{^1H\}$ NMR (101 MHz, $CDCl_3$) δ: 144.6, 141.0, 140.2, 139.0, 129.5, 128.9, 128.6, 127.34, 127.31, 127.2, 127.0, 126.6, 57.4, 46.4 ppm. IR (neat): 3443, 3389, 3027, 1630, 1601, 1561, 1486, 1452, 750, 698 cm^{-1}. HRMS: calcd for $C_{20}H_{19}N$ $[M+H]^+$ 274.1596, found 274.1599.

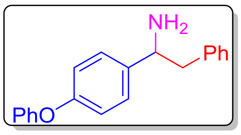

1-(4-Phenoxyphenyl)-2-phenylethan-1-amine (3ra) The reaction was performed following the General Procedure A with 4-phenoxybenzaldehyde **(1r)** (35.0 mg, 0.20 mmol), $NaN(SiMe_3)_2$ (73.2 mg, 0.40 mmol), CsTFA (17.2 mg, 0.07 mmol) and toluene **(2a)** (2 mL). The crude product was purified by chromatography on silica gel (eluted with Petroleum ether:EtOAc:Et_3N = 100:50:7) to give the desired product (53.2 mg, 92% yield) as a white solid. Mp 50–52 °C. R_f = 0.47 (Petroleum ether:EtOAc:Et_3N = 100:50:7). ^{1}H NMR (400 MHz, $CDCl_3$) δ: 7.34 – 7.27 (m, 6H), 7.24 – 7.16 (m, 3H), 7.10 – 7.07 (m, 1H), 7.01 – 6.95 (m, 4H), 4.18 (dd, *J* = 8.8, 5.0 Hz, 1H), 3.00 (dd, *J* = 13.3, 5.0 Hz, 1H), 2.82 (dd, *J* = 13.3, 8.8 Hz, 1H), 1.64 (brs, 2H) ppm. $^{13}C\{^1H\}$ NMR (101 MHz, $CDCl_3$) δ: 157.6, 156.2, 140.7, 139.1, 129.8, 129.5, 128.5, 127.9, 126.5, 123.2,

119.0, 118.8, 57.1, 46.7 ppm. IR (neat): 3375, 3310, 3027, 1589, 1505, 1489, 1454, 1237, 750, 699 cm^{-1}. HRMS: calcd for $C_{20}H_{19}NO$ $[M+H]^+$ 290.1500, found 290.1495.

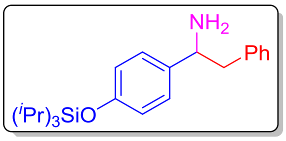

2-Phenyl-1-(4-((triisopropylsilyl)oxy)phenyl)ethan-1-amine (3sa) The reaction was performed following the General Procedure A with 4-((triisopropylsilyl)oxy)benzaldehyde **(1s)** (58.7 μL, 0.20 mmol), $NaN(SiMe_3)_2$ (73.2 mg, 0.40 mmol), CsTFA (17.2 mg, 0.07 mmol) and toluene **(2a)** (2 mL). The crude product was purified by chromatography on silica gel (eluted with Petroleum ether:EtOAc:Et_3N = 100:50:7) to give the desired product (56.8 mg, 77% yield) as a yellow oil. R_f = 0.55 (Petroleum ether:EtOAc:Et_3N = 100:50:7). ^1H NMR (400 MHz, $CDCl_3$) δ: 7.27 – 7.10 (m, 7H), 6.84 – 6.81 (m, 2H), 4.11 (dd, J = 8.3, 5.4 Hz, 1H), 2.95 (dd, J = 13.2, 5.4 Hz, 1H), 2.81 (dd, J = 13.2, 8.3 Hz, 1H), 1.57 (brs, 2H), 1.29 – 1.20 (m, 3H), 1.09 (d, J = 7.4 Hz, 18H) ppm. $^{13}C\{^1H\}$ NMR (101 MHz, $CDCl_3$) δ: 155.1, 139.2, 138.1, 129.5, 128.4, 127.4, 126.4, 119.8, 57.1, 46.7, 18.0, 12.8 ppm. IR (neat): 3380, 3318, 2944, 2866, 1606, 1581, 1509, 1463, 1454, 1261, 747, 699 cm^{-1}. HRMS: calcd for $C_{23}H_{35}NOSi$ $[M+H]^+$ 370.2521, found 370.2520.

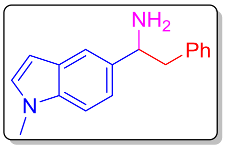

1-(1-Methyl-1H-indol-5-yl)-2-phenylethan-1-amine (3ta) The reaction was performed following the General Procedure A with 1-methyl-1H-indole-5-carbaldehyde **(1t)** (31.8 mg, 0.20 mmol), $NaN(SiMe_3)_2$ (73.2 mg, 0.40 mmol), CsTFA (17.2 mg, 0.07 mmol) and toluene **(2a)** (2 mL). The crude product was purified by chromatography on silica gel (eluted with Petroleum ether:EtOAc:Et_3N = 100:50:7) to give the desired product (41.0 mg, 82% yield) as a yellow oil. R_f = 0.40 (Petroleum ether:EtOAc:Et_3N = 100:50:7). ^1H NMR (400 MHz, $CDCl_3$) δ: 7.61 (s, 1H), 7.31 – 7.21 (m, 7H), 7.04 (d, J = 3.1 Hz, 1H), 6.45 (d, J = 3.0 Hz, 1H), 4.30 (dd, J = 9.0, 4.8 Hz, 1H), 3.80 (s, 3H), 3.08 (dd, J = 13.3, 4.8 Hz, 1H), 2.89 (dd, J = 13.3, 9.0 Hz, 1H), 1.65 (brs, 2H) ppm. $^{13}C\{^1H\}$ NMR (101 MHz, $CDCl_3$) δ: 139.8, 136.9, 136.2, 129.5, 129.3, 128.6, 128.5, 126.4, 120.6, 118.5, 109.3, 101.0, 58.0, 47.0, 33.0 ppm. IR (neat): 3377, 3294, 2925, 1595, 1567, 1508, 1471, 1442, 1259, 1222, 1164, 749, 660 cm^{-1}. HRMS: calcd for $C_{17}H_{18}N_2$ $[M+H]^+$ 251.1548, found 251.1548.

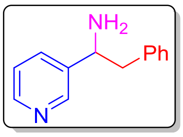

2-Phenyl-1-(pyridin-3-yl)ethan-1-amine (3ua) The reaction was performed following the General Procedure A with 3-pyridinealdehyde **(1u)** (18.7 μL, 0.20 mmol), $NaN(SiMe_3)_2$ (73.2 mg, 0.40 mmol), CsTFA (17.2 mg, 0.07 mmol) and toluene **(2a)** (2 mL). The crude product was purified by chromatography on silica gel (eluted with Petroleum ether:EtOAc:Et_3N = 100:5) to give the desired product (33.2 mg, 84% yield) as a brown oil. R_f = 0.36 (Petroleum ether:EtOAc:Et_3N = 50:50:5). ^1H NMR (400 MHz, $CDCl_3$) δ: 8.56 – 8.49 (m, 2H), 7.71 – 7.68 (m, 1H), 7.32 – 7.21 (m, 4H), 7.17 – 7.14 (m, 2H), 4.25 (dd, J = 8.7, 5.3 Hz, 1H), 2.99 (dd, J = 13.3, 5.3 Hz, 1H), 2.85 (dd, J = 13.3, 8.6 Hz, 1H), 1.67 (brs, 2H) ppm. $^{13}C\{^1H\}$ NMR (101 MHz, $CDCl_3$) δ: 148.76, 148.72, 140.8, 138.3, 134.2, 129.4, 128.7, 126.8, 123.5, 55.4, 46.4 ppm. The spectroscopic data for this product match the literature data.[5]

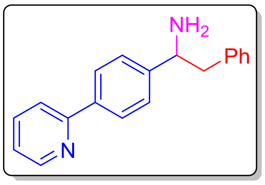

2-Phenyl-1-(3-(pyridin-2-yl)phenyl)ethan-1-amine (3va) The reaction was performed following the General Procedure A with 3-(pyridin-2-yl)benzaldehyde **(1v)** (36.6 mg, 0.20 mmol), $NaN(SiMe_3)_2$ (73.2 mg, 0.40 mmol), CsTFA (17.2 mg, 0.07 mmol) and toluene **(2a)** (2 mL). The crude product was purified by chromatography on silica gel (eluted with Petroleum ether:EtOAc:Et_3N = 100:50:7) to give the desired product (42.7 mg, 78% yield) as a yellow oil. R_f

= 0.34 (Petroleum ether:EtOAc:Et_3N = 100:50:7). ^{1}H NMR (400 MHz, $CDCl_3$) δ: 8.68 – 8.67 (m, 1H), 7.96 – 7.94 (m, 2H), 7.75 – 7.70 (m, 2H), 7.47 – 7.45 (m, 2H), 7.30 – 7.26 (m, 2H), 7.24 – 7.16 (m, 4H), 4.27 (dd, *J* = 8.4, 5.5 Hz, 1H), 3.06 (dd, *J* = 13.3, 5.5 Hz, 1H), 2.92 (dd, *J* = 13.3, 8.4 Hz, 1H), 1.63 (brs, 2H) ppm. ^{13}C{^{1}H} NMR (101 MHz, $CDCl_3$) δ: 157.3, 149.8, 145.9, 138.8, 138.5, 136.9, 129.5, 128.6, 127.13, 127.11, 126.6, 122.2, 120.6, 57.5, 46.2 ppm. IR (neat): 3426, 3376, 2922, 1588, 1566, 1508, 1438, 1260, 750, cm^{-1}. HRMS: calcd for $C_{19}H_{18}N_2$ [M+H]$^+$ 275.1504, found 275.1507.

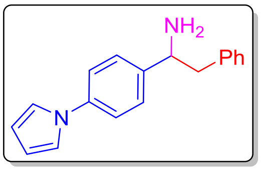

1-(4-(1*H*-pyrrol-1-yl)phenyl)-2-phenylethan-1-amine (3wa) The reaction was performed following the General Procedure A with 4-(1H-pyrrol-1-yl)benzaldehyde **(1w)** (34.2 mg, 0.20 mmol), $NaN(SiMe_3)_2$ (73.2 mg, 0.40 mmol), CsTFA (17.2 mg, 0.07 mmol) and toluene **(2a)** (2 mL). The crude product was purified by chromatography on silica gel (eluted with Petroleum ether:EtOAc:Et_3N = 100:50:7) to give the desired product (42.9 mg, 82% yield) as a yellow oil. R_f = 0.48 (Petroleum ether:EtOAc:Et_3N = 100:50:7). ^{1}H NMR (400 MHz, $CDCl_3$) δ: 7.41 – 7.38 (m, 2H), 7.36 – 7.33 (m, 2H), 7.31 – 7.27 (m, 2H), 7.24 – 7.20 (m, 1H), 7.18 – 7.15 (m, 2H), 7.10 – 7.07 (m, 2H), 6.35 – 6.33 (m, 2H), 4.22 (dd, *J* = 8.7, 5.1 Hz, 1H), 3.00 (dd, *J* = 13.3, 5.1 Hz, 1H), 2.84 (dd, *J* = 13.3, 8.7 Hz, 1H), 1.51 (brs, 2H) ppm. ^{13}C{^{1}H} NMR (101 MHz, $CDCl_3$) δ: 143.1, 139.8, 138.9, 129.5, 128.6, 127.7, 126.6, 120.5, 119.4, 110.4, 57.1, 46.6 ppm. IR (neat): 3472, 3353, 2930, 1612, 1587, 1525, 1494, 1454, 1326, 723 cm^{-1}. HRMS: calcd for $C_{18}H_{18}N_2$ [M+H]$^+$ 263.1504, found 263.1507.

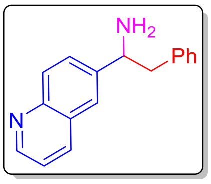

2-Phenyl-1-(quinolin-6-yl)ethan-1-amine (3xa) The reaction was performed following the General Procedure A with quinoline-6-carbaldehyde **(1x)** (31.4 mg, 0.20 mmol), $NaN(SiMe_3)_2$ (73.2 mg, 0.40 mmol), CsTFA (17.2 mg, 0.07 mmol) and toluene **(2a)** (2 mL). The crude product was purified by chromatography on silica gel (eluted with Petroleum ether:EtOAc:Et_3N = 100:50:7) to give the desired product (34.2 mg, 69% yield) as a yellow oil. R_f = 0.17 (Petroleum ether:EtOAc:Et_3N = 100:50:7). ^{1}H NMR (400 MHz, $CDCl_3$) δ: 8.90 – 8.88 (m, 1H), 8.13 – 8.08 (m, 2H), 7.78 – 7.74 (m, 2H), 7.41 –7.37 (m, 1H), 7.31 – 7.29 (m, 2H), 7.28 – 7.17 (m, 3H), 4.41 (dd, *J* = 8.7, 5.1 Hz, 1H), 3.11 (dd, *J* = 13.4, 5.1 Hz, 1H), 2.90 (dd, *J* = 13.3, 8.7 Hz, 1H), 1.73 (brs, 2H) ppm. ^{13}C{^{1}H} NMR (101 MHz, $CDCl_3$) δ: 150.3, 147.9, 143.9, 138.7, 136.1, 129.7, 129.5, 128.8, 128.7, 128.3, 126.7, 124.8, 121.4, 57.5, 46.5 ppm. IR (neat): 3361, 3306, 2921, 2852, 1595, 1574, 1555, 1500, 1454, 839, 701 cm^{-1}. HRMS: calcd for $C_{17}H_{16}N_2$ [M+H]$^+$ 249.1347, found 249.1351.

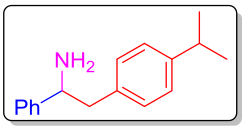

2-(4-Isopropylphenyl)-1-phenylethan-1-amine (3ab) The reaction was performed following the General Procedure B with benzaldehyde **(1a)** (20 μL, 0.20 mmol), $NaN(SiMe_3)_2$ (73.2 mg, 0.40 mmol), CsTFA (17.2 mg, 0.07 mmol) 4-isopropyltoluene **(2b)** (1 mL) and cyclohexane (1 mL). The crude product was purified by chromatography on silica gel (eluted with Petroleum ether:EtOAc:Et_3N = 100:20:5) to give the desired product (37.2 mg, 78% yield) as a white solid. Mp 38–40 ^{o}C. R_f = 0.54 (Petroleum ether:EtOAc:Et_3N = 100:20:5). ^{1}H NMR (400 MHz, $CDCl_3$) δ: 7.40 – 7.32 (m, 4H), 7.28 – 7.23 (m, 1H), 7.17 – 7.12 (m, 4H), 4.17 (dd, *J* = 9.3, 4.4 Hz, 1H), 2.99 (dd, *J* = 13.4, 4.4 Hz, 1H), 2.92 – 7.85 (m, 1H), 2.76 (dd, *J* = 13.4, 9.4 Hz, 1H), 1.51 (brs, 2H), 1.24 (d, *J* = 6.9 Hz, 6H) ppm. ^{13}C{^{1}H} NMR (101 MHz, $CDCl_3$) δ: 147.1, 146.0, 136.6, 129.4, 128.5, 127.2, 126.62, 126.55, 57.6, 46.2, 33.9, 24.2 ppm. IR (neat): 3371, 3297, 2959, 1602, 1514, 1453, 1383, 814, 700 cm^{-1}. HRMS: calcd for $C_{17}H_{21}N$ [M+H]$^+$ 240.1708, found 240.1704.

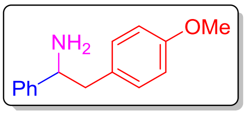

2-(4-Methoxyphenyl)-1-phenylethan-1-amine (3ac) The reaction was performed following the General Procedure B with benzaldehyde **(1a)** (20 μL, 0.20 mmol), $NaN(SiMe_3)_2$ (73.2 mg, 0.40 mmol), CsTFA (17.2 mg, 0.07 mmol) and 1-methoxy-4-methylbenzene **(2c)** (2 mL). The crude product was purified by chromatography on silica gel (eluted with Petroleum ether:EtOAc:Et_3N = 100:20:5) to give the desired product (30.0 mg, 66% yield) as a brown oil. R_f = 0.54 (Petroleum ether:EtOAc:Et_3N = 100:20:5). ^1H NMR (400 MHz, $CDCl_3$) δ: 7.36– 7.24 (m, 5H), 7.08 (d, J = 8.2 Hz, 2H), 6.82 (d, J = 8.3 Hz, 2H), 4.15 (dd, J = 8.8, 5.0 Hz, 1H), 3.79 (s, 3H), 2.95 (dd, J = 13.5, 4.9 Hz, 1H), 2.77 (dd, J = 13.5, 8.8 Hz, 1H), 1.55 (brs, 2H) ppm. $^{13}C\{^1H\}$ NMR (101 MHz, $CDCl_3$) δ: 158.3, 145.8, 131.2, 130.4, 128.5, 127.2, 126.6, 114.0, 57.8, 55.4, 45.7 ppm. IR (neat): 3438, 3394, 2920, 1617, 1584, 1512, 1455, 764, 751 cm^{-1}. HRMS: calcd for $C_{15}H_{17}NO$ $[M+H]^+$ 228.1388, found 228.1387.

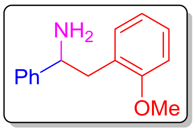

2-(2-Methoxyphenyl)-1-phenylethan-1-amine (3ad) The reaction was performed following the General Procedure B with benzaldehyde **(1a)** (20 μL, 0.20 mmol), $NaN(SiMe_3)_2$ (73.2 mg, 0.40 mmol), CsTFA (17.2 mg, 0.07 mmol) 1-methoxy-2-methylbenzene **(2d)** (1 mL) and cyclohexane (1 mL). The crude product was purified by chromatography on silica gel (eluted with Petroleum ether:EtOAc:Et_3N = 100:20:5) to give the desired product (34.9 mg, 77% yield) as a light yellow oil. R_f = 0.54 (Petroleum ether:EtOAc:Et_3N = 100:20:5). ^1H NMR (400 MHz, $CDCl_3$) δ: 7.39 – 7.30 (m, 4H), 7.25 – 7.19 (m, 2H), 7.09 – 7.06 (m, 1H), 6.88 – 6.84 (m, 2H), 4.24 (dd, J = 8.9, 4.8 Hz, 1H), 3.82 (s, 3H), 3.05 (dd, J = 13.1, 4.8 Hz, 1H), 2.81 (dd, J = 13.1, 8.9 Hz, 1H), 1.53 (brs, 2H) ppm. $^{13}C\{^1H\}$ NMR (101 MHz, $CDCl_3$) δ: 157.8, 146.5, 131.3, 128.4, 127.8, 127.7, 127.0, 126.5, 120.4, 110.5, 55.9, 55.4, 41.3 ppm. IR (neat): 3373, 3304, 2920, 1601, 1586, 1493, 1455, 1243, 750, 700 cm^{-1}. HRMS: calcd for $C_{15}H_{17}NO$ $[M+H]^+$ 228.1344, found 228.1345.

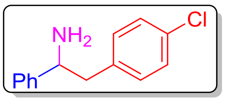

2-(4-Chlorophenyl)-1-phenylethan-1-amine (3ae) The reaction was performed following the General Procedure B with benzaldehyde **(1a)** (20 μL, 0.20 mmol), $NaN(SiMe_3)_2$ (73.2 mg, 0.40 mmol), CsTFA (17.2 mg, 0.07 mmol) and 1-chloro-4-methylbenzene **(2e)** (2 mL). The crude product was purified by chromatography on silica gel (eluted with Petroleum ether:EtOAc:Et_3N = 100:20:5) to give the desired product (23.1 mg, 50% yield) as a brown oil. R_f = 0.60 (Petroleum ether:EtOAc:Et_3N = 100:20:5). ^1H NMR (400 MHz, $CDCl_3$) δ: 7.35 – 7.30 (m, 4H), 7.29 – 7.21 (m, 3H), 7.08 – 7.05 (m, 2H), 4.15 (dd, J = 8.3, 5.4 Hz, 1H), 2.95 (dd, J = 13.4, 5.4 Hz, 1H), 2.82 (dd, J = 13.4, 8.4 Hz, 1H), 1.51 (brs, 2H) ppm. $^{13}C\{^1H\}$ NMR (101 MHz, $CDCl_3$) δ: 145.4, 137.6, 132.3, 130.8, 128.6, 127.3, 126.5, 57.6, 45.9 ppm. One resonance was not observed due to overlapping resonances. IR (neat): 3411, 3373, 2919, 2852, 1597, 1555, 1535, 1491, 1453, 1092, 1027, 805, 700cm^{-1}. HRMS: calcd for $C_{14}H_{14}ClN$ $[M+H]^+$ 233.0785, found 233.0786.

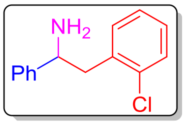

2-(2-Chlorophenyl)-1-phenylethan-1-amine (3af) The reaction was performed following the General Procedure B with benzaldehyde **(1a)** (20 μL, 0.20 mmol), $NaN(SiMe_3)_2$ (73.2 mg, 0.40 mmol), CsTFA (17.2 mg, 0.07 mmol) and 1-chloro-2-methylbenzene **(2f)** (1 mL) and cyclohexane (1 mL). The crude product was purified by chromatography on silica gel (eluted with Petroleum ether:EtOAc:Et_3N = 100:20:5) to give the desired product (39.9 mg, 86% yield) as a light yellow oil. R_f = 0.52 (Petroleum ether:EtOAc:Et_3N = 100:20:5). ^1H NMR (400 MHz, $CDCl_3$) δ: 7.39 – 7.31 (m, 5H), 7.28 – 7.24 (m, 1H), 7.19 – 7.12 (m, 3H), 4.30 (dd, J = 8.7, 5.1 Hz, 1H), 3.14 (dd, J = 13.4, 5.1 Hz, 1H), 2.96 (dd, J = 13.4, 8.8 Hz, 1H), 1.81 (brs, 2H) ppm. $^{13}C\{^1H\}$ NMR (101 MHz, $CDCl_3$) δ: 145.5, 136.9, 134.5, 131.9, 129.8, 128.6, 128.1, 127.3, 126.8, 126.4, 55.7, 44.3 ppm. IR (neat): 3373, 3304, 2925, 1603, 1571, 1561, 1492, 1452, 755, 700cm^{-1}. HRMS:

calcd for $C_{14}H_{14}ClN$ [M+H]$^+$ 232.0893, found 232.0898.

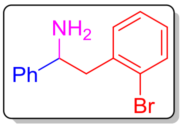

2-(2-Bromophenyl)-1-phenylethan-1-amine (3ag) The reaction was performed following the General Procedure B with benzaldehyde **(1a)** (20 µL, 0.20 mmol), $NaN(SiMe_3)_2$ (73.2 mg, 0.40 mmol), CsTFA (17.2 mg, 0.07 mmol) 1-bromo-2-methylbenzene **(2g)** (1 mL) and cyclohexane (1 mL). The crude product was purified by chromatography on silica gel (eluted with Petroleum ether:EtOAc:Et_3N = 100:20:5) to give the desired product (46.9 mg, 85% yield) as a light yellow oil. R_f = 0.52 (Petroleum ether:EtOAc:Et_3N = 100:20:5). ^{1}H NMR (400 MHz, $CDCl_3$) δ: 7.57 – 7.55 (m, 1H), 7.40 – 7.37 (m, 2H), 7.35 – 7.31 (m, 2H), 7.27 – 7.23 (m, 1H), 7.21 – 7.17 (m, 1H), 7.13 – 7.05 (m, 2H), 4.31 (dd, *J* = 8.8, 5.1 Hz, 1H), 3.14 (dd, *J* = 13.4, 5.0 Hz, 1H), 2.95 (dd, *J* = 13.3, 8.8 Hz, 1H), 1.60 (brs, 2H) ppm. ^{13}C{^{1}H} NMR (101 MHz, $CDCl_3$) δ: 145.4, 138.5, 133.1, 131.9, 128.6, 128.3, 127.4, 127.3, 126.4, 125.0, 55.6, 46.7 ppm. IR (neat): 3369, 3295, 3060, 3027, 1603, 1566, 1493, 1470, 1453, 1024, 758, 700 cm^{-1}. HRMS: calcd for $C_{14}H_{14}BrN$ [M+H]$^+$ 277.0389, found 277.0385.

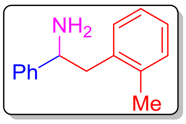

1-Phenyl-2-(*o*-tolyl)ethan-1-amine (3ah) The reaction was performed following the General Procedure B with benzaldehyde **(1a)** (20 µL, 0.20 mmol), $NaN(SiMe_3)_2$ (73.2 mg, 0.40 mmol), CsTFA (17.2 mg, 0.07 mmol) *o*-xylene **(2h)** (1 mL) and cyclohexane (1 mL). The crude product was purified by chromatography on silica gel (eluted with Petroleum ether:EtOAc:Et_3N = 100:20:5) to give the desired product (34.6 mg, 82% yield) as a light yellow oil. R_f = 0.48 (Petroleum ether:EtOAc:Et_3N = 100:20:5). ^{1}H NMR (400 MHz, $CDCl_3$) δ: 7.38 – 7.31 (m, 4H), 7.28 – 7.23 (m, 1H), 7.16 – 7.11 (m, 4H), 4.19 (dd, *J* = 8.9, 4.9 Hz, 1H), 3.00 (dd, *J* = 13.6, 4.9 Hz, 1H), 2.85 (dd, *J* = 13.6, 8.9 Hz, 1H), 2.30 (s, 3H), 1.54 (brs, 2H) ppm. ^{13}C{^{1}H} NMR (101 MHz, $CDCl_3$) δ: 146.0, 137.5, 136.7, 130.5, 130.3, 128.6, 127.2, 126.6, 126.4, 126.0, 56.4, 44.0, 19.7 ppm. IR (neat): 3373, 3295, 3025, 2920, 1604, 1589, 1492, 1453, 753, 700 cm^{-1}. HRMS: calcd for $C_{15}H_{17}N$ [M+H]$^+$ 212.1395, found 212.1400.

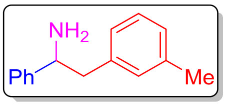

1-Phenyl-2-(*m*-tolyl)ethan-1-amine (3ai) The reaction was performed following the General Procedure B with benzaldehyde **(1a)** (20 µL, 0.20 mmol), $NaN(SiMe_3)_2$ (73.2 mg, 0.40 mmol), CsTFA (17.2 mg, 0.07 mmol), *m*-xylene **(2i)** (1 mL) and cyclohexane (1 mL). The crude product was purified by chromatography on silica gel (eluted with Petroleum ether:EtOAc:Et_3N = 100:20:5) to give the desired product (35.5 mg, 84% yield) as a white solid. Mp 32–34 °C. R_f = 0.50 (Petroleum ether:EtOAc:Et_3N = 100:20:5). ^{1}H NMR (400 MHz, $CDCl_3$) δ: 7.39 – 7.32 (m, 4H), 7.28 – 7.24 (m, 1H), 7.20 – 7.17 (m, 1H), 7.05 – 6.98 (m, 3H), 4.19 (dd, *J* = 9.3, 4.6 Hz, 1H), 2.98 (dd, *J* = 13.3, 4.6 Hz, 1H), 2.76 (dd, *J* = 13.3, 9.3 Hz, 1H), 2.33 (s, 3H), 1.50 (brs, 2H) ppm. ^{13}C{^{1}H} NMR (101 MHz, $CDCl_3$) δ: 145.9, 139.2, 138.1, 130.3, 128.5, 128.5, 127.3, 127.2, 126.5, 126.4, 57.6, 46.6, 21.5 ppm. IR (neat): 3368, 3300, 3027, 2918, 1607, 1589, 1493, 1453, 774, 700 cm^{-1}. HRMS: calcd for $C_{15}H_{17}N$ [M+H]$^+$ 212.1439, found 212.1436.

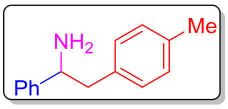

1-Phenyl-2-(*p*-tolyl)ethan-1-amine (3aj) The reaction was performed following the General Procedure B with benzaldehyde **(1a)** (20 µL, 0.20 mmol), $NaN(SiMe_3)_2$ (73.2 mg, 0.40 mmol), CsTFA (17.2 mg, 0.07 mmol), *p*-xylene **(2j)** (1 mL) and cyclohexane (1 mL). The crude product was purified by chromatography on silica gel (eluted with Petroleum ether:EtOAc:Et_3N = 100:20:5) to give the product (34.2 mg, 81% yield) as a light yellow oil. R_f = 0.50 (Petroleum ether:EtOAc:Et_3N = 100:20:5). ^{1}H NMR (400 MHz, $CDCl_3$) δ: 7.37 – 7.23 (m, 5H), 7.11 – 7.05 (m, 4H), 4.16 (dd, *J* = 9.0, 4.7 Hz, 1H), 2.98 (dd, *J* = 13.4, 4.8 Hz, 1H), 2.77 (dd, *J* = 13.4, 9.0 Hz, 1H), 1.54

(brs, 2H) ppm. $^{13}C\{^1H\}$ NMR (101 MHz, $CDCl_3$) δ: 145.6, 136.1, 136.0, 129.4, 129.3, 128.6, 127.2, 126.6, 57.7, 46.0, 21.2 ppm. The spectroscopic data for this product match the literature data.[6]

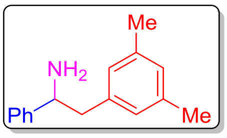

2-(3,5-Dimethylphenyl)-1-phenylethan-1-amine (3ak) The reaction was performed following the General Procedure B with benzaldehyde **(1a)** (20 µL, 0.20 mmol), $NaN(SiMe_3)_2$ (73.2 mg, 0.40 mmol), CsTFA (17.2 mg, 0.07 mmol) mesitylene **(2k)** (1 mL) and cyclohexane (1 mL). The crude product was purified by chromatography on silica gel (eluted with Petroleum ether:EtOAc:Et_3N = 100:20:5) to give the desired product (43.2 mg, 96% yield) as a yellow oil. R_f = 0.50 (Petroleum ether:EtOAc:Et_3N = 100:20:5). ^1H NMR (400 MHz, $CDCl_3$) δ: 7.38 – 7.29 (m, 4H), 7.25 – 7.20 (m, 1H), 6.84 – 6.80 (m, 3H), 4.14 (dd, *J* = 9.6, 4.2 Hz, 1H), 2.92 (dd, *J* = 13.3, 4.1 Hz, 1H), 2.66 (dd, *J* = 13.2, 9.6 Hz, 1H), 2.26 (s, 6H), 1.54 (brs, 2H) ppm. $^{13}C\{^1H\}$ NMR (101 MHz, $CDCl_3$) δ: 146.0, 139.1, 138.1, 128.5, 128.2, 127.3, 127.2, 126.5, 57.5, 46.5, 21.4 ppm. IR (neat): 3453, 3376, 3024, 2917, 1605, 1493, 1454, 751, 700 cm^{-1}. HRMS: calcd for $C_{16}H_{19}N$ $[M+H]^+$ 226.1551, found 226.1551.

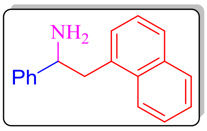

2-(Naphthalen-1-yl)-1-phenylethan-1-amine (3al) The reaction was performed following the General Procedure B with benzaldehyde **(1a)** (20 µL, 0.20 mmol), $NaN(SiMe_3)_2$ (73.2 mg, 0.40 mmol), CsTFA (17.2 mg, 0.07 mmol) 1-methylnaphthalene **(2l)** (1 mL) and cyclohexane (1 mL). The crude product was purified by chromatography on silica gel (eluted with Petroleum ether:EtOAc:Et_3N = 100:20:5) to give the desired product (30.6 mg, 62% yield) as a white solid. Mp 66–68 °C. R_f = 0.52 (PE:EtOAc:Et_3N = 100:20:5). ^1H NMR (400 MHz, $CDCl_3$) δ: 8.15 – 8.12 (m, 1H), 7.89 – 7.86 (m, 1H), 7.77 – 7.75 (m, 1H), 7.57 – 7.48 (m, 2H), 7.46 – 7.43 (m, 2H), 7.41 – 7.25 (m, 5H), 4.38 (dd, *J* = 9.4, 3.6 Hz, 1H), 3.52 (dd, *J* = 13.5,3.6 Hz, 1H), 3.21 (dd, *J* = 13.4, 9.3 Hz, 1H), 1.55 (brs, 2H) ppm. $^{13}C\{^1H\}$ NMR (101 MHz, $CDCl_3$) δ: 146.1, 135.3, 134.1, 132.2, 129.0, 128.6, 127.8, 127.4, 127.3, 126.5, 126.1, 125.7, 125.5, 123.9, 56.6, 43.9 ppm. IR (neat): 3446, 3442, 2929, 2864, 1633, 1509, 1492, 764, 699 cm^{-1}. HRMS: calcd for $C_{18}H_{17}N$ $[M+H]^+$ 248.1439, found 248.1439.

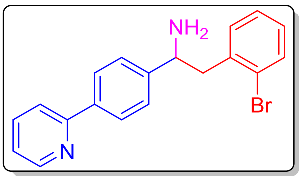

2-(2-Bromophenyl)-1-(3-(pyridin-2-yl)phenyl)ethan-1-amine (3vg) The reaction was performed following the General Procedure B with 4-(2-Pyridinyl)benzaldehyde **(1v)** (183.2 mg, 1.0 mmol), $NaN(SiMe_3)_2$ (366.8 mg, 2.0 mmol), CsTFA (86.1 mg, 3.5 mmol) 1-bromo-2-methylbenzene **(2g)** (5 mL) and cyclohexane (5 mL). The crude product was purified by chromatography on silica gel (eluted with Petroleum ether:EtOAc:Et_3N = 100:50:7) to give the desired product (312.5 mg, 88% yield) as a brown oil. R_f = 0.39 (Petroleum ether:EtOAc:Et_3N = 100:50:7). ^1H NMR (400 MHz, $CDCl_3$) δ: 8.70 – 7.68 (m, 1H), 7.98 – 7.95 (m, 2H), 7.77 – 7.71 (m, 2H), 7.58 – 7.54 (m, 1H), 7.50 – 7.48 (m, 2H), 7.24 – 7.17 (m, 2H), 7.13 – 7.06 (m, 2H), 4.39 (dd, *J* = 8.5, 5.3 Hz, 1H), 3.17 (dd, *J* = 13.3, 5.4 Hz, 1H), 3.00 (dd, *J* = 13.3, 8.5 Hz, 1H), 1.62 (brs, 2H) ppm. $^{13}C\{^1H\}$ NMR (101 MHz, $CDCl_3$) δ: 157.3, 149.7, 146.4, 138.4, 138.3, 136.8, 133.0, 131.9, 128.3, 127.4, 127.0, 126.8, 125.0, 122.1, 120.5, 55.4, 46.7 ppm. IR (neat): 3455, 3370, 3009, 2923, 1601, 1587, 1561, 1467, 1435, 780, 753 cm^{-1}. HRMS: calcd for $C_{19}H_{17}N_2Br$ $[M+H]^+$ 354.0555, found 354.0551.

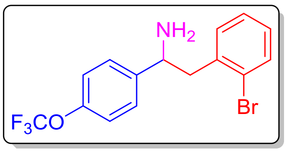

2-(2-Bromophenyl)-1-(4-(trifluoromethoxy)phenyl)ethan-1-amine (3ng) The reaction was performed following the General Procedure B with 4-(trifluoromethoxy)benzaldehyde **(1n)** (190.1 mg, 1.0 mmol), $NaN(SiMe_3)_2$ (366.8 mg, 2.0 mmol), CsTFA (86.1 mg, 3.5 mmol)

1-bromo-2-methylbenzene **(2g)** (5 mL) and cyclohexane (5 mL). The crude product was purified by chromatography on silica gel (eluted with Petroleum ether:EtOAc:Et_3N = 100:50:7) to give the desired product (263.8 mg, 73% yield) as a brown oil. R_f = 0.42 (Petroleum ether:EtOAc:Et_3N = 100:50:7). ^{1}H NMR (400 MHz, $CDCl_3$) δ: 7.57 – 7.55 (m, 1H), 7.42 – 7.40 (m, 2H), 7.21 – 7.15 (m, 3H), 7.11 – 7.06 (m, 2H), 4.34 (dd, J = 8.7, 5.1 Hz, 1H), 3.10 (dd, J = 13.3, 5.1 Hz, 1H), 2.92 (dd, J = 13.3, 8.7 Hz, 1H), 1.56 (brs, 2H) ppm. ^{13}C{^{1}H} NMR (101 MHz, $CDCl_3$) δ: 148.3 (q, $J^2_{C\text{-}F}$ = 1.8 Hz), 144.2, 138.2, 133.1, 131.9, 128.4, 127.8, 127.4, 125.0, 121.0, 120.6 (q, $J^1_{C\text{-}F}$ = 257.8 Hz), 54.9, 46.8 ppm. IR (neat): 3377, 3302, 3057, 2925, 1595, 1567, 1508, 1471, 1442, 1266, 1224, 1166, 1025, 750 cm^{-1}. HRMS: calcd for $C_{15}H_{13}BrF_3NO$ $[M+H]^+$ 360.0211, found 360.0209.

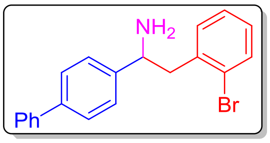

1-[(1,1'-Biphenyl)-4-yl]-2-(2-bromophenyl)ethan-1-amine (3qg) The reaction was performed following the General Procedure B with [1,1'-biphenyl]-4-carbaldehyde **(1q)** (182.2 mg, 1.0 mmol), $NaN(SiMe_3)_2$ (366.8 mg, 2.0 mmol), CsTFA (86.1 mg, 3.5 mmol), 1-bromo-2-methylbenzene **(2g)** (5 mL) and cyclohexane (5 mL). The crude product was purified by chromatography on silica gel (eluted with Petroleum ether:EtOAc:Et_3N = 100:50:7) to give the desired product (296.9 mg, 84% yield) as a yellow oil. R_f = 0.42 (Petroleum ether:EtOAc:Et_3N = 100:50:7). ^{1}H NMR (400 MHz, $CDCl_3$) δ: 7.60 – 7.55 (m, 5H), 7.47 – 7.41 (m, 4H), 7.35 – 7.31 (m, 1H), 7.23 – 7.13 (m, 2H), 7.10 – 7.06 (m, 1H), 4.37 (dd, J = 8.9, 4.9 Hz, 1H), 3.18 (dd, J = 13.4, 4.9, Hz, 1H), 2.97 (dd, J = 13.4, 8.9, Hz, 1H), 1.55 (brs, 2H) ppm. ^{13}C{^{1}H} NMR (101 MHz, $CDCl_3$) δ: 144.6, 141.0, 140.1, 138.5, 133.0, 131.9, 128.8, 128.3, 127.4, 127.24, 127.19, 127.1, 126.8, 125.0, 55.2, 46.7 ppm. IR (neat): 3373, 3307, 3028, 2924, 1599, 1566, 1517, 1486, 1471, 1441, 1024, 751 cm^{-1}. HRMS: calcd for $C_{20}H_{18}NBr[M+H]^+$ 353.0602, found 353.0604.

Synthesis of 1,2-diphenylethan-1-aminium chloride salt

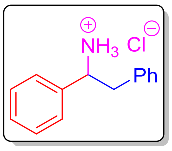

To an oven-dried microwave vial equipped with a stir bar under argon atmosphere inside a glove box was added $NaN(SiMe_3)_2$ (73.2 mg, 0.40 mmol), CsTFA (17.2 mg, 0.07 mmol) and toluene (2 mL). Then the benzaldehyde (0.20 mmol) was added via syringe. The microwave vial was sealed with a a cap and removed from the glove box. The reaction mixture was heated to 110 ℃ in an oil bath and stirred for 12 h. The sealed vial was cooled to room temperature, opened to air, and then 5 drops of water was added. The reaction mixture was passed through a short pad of silica, washed with an additional 6 mL of ethyl acetate (3 × 2 mL), and the combined solutions were concentrated *in vacuo* to give the crude material. Then HCl (0.1 mL, 10 N) was added to the crude material at rt and a light yellow soild precipitate was observed immediately. The light yellow soild was filtered and washed with cold Et_2O (1.0 × 3 mL) to the desired aminium chloride salt.

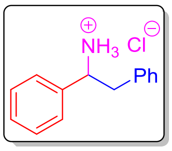

1,2-diphenylethan-1-aminium chloride (3'aa) The reaction was performed following the General Procedure C with $NaN(SiMe_3)_2$ (73.2 mg, 0.40 mmol), cesium trifluoroacetate (CsTFA) (17.2 mg, 0.07 mmol) , toluene (2 mL) and benzaldehyde (0.20 mmol). Then it give desired product (36.5 mg, 78% yield) as a light yellow solid. ^{1}H NMR (400 MHz, MeOD) δ: 7.41 – 7.35 (m, 5H), 7.26 – 7.19 (m, 3H), 7.12 – 7.10 (m, 2H), 4.51 (dd, J = 9.1, 6.4 Hz, 1H), 3.20 (dd, J = 13.5, 9.1 Hz, 1H) ppm. ^{13}C{^{1}H} NMR (101 MHz, MeOD) δ: 138, 137, 130.4, 130.3, 130.2, 130, 128.5, 128.3, 58, 42 ppm. The spectroscopic data for this product match the literature data.[7]

Synthesis of 2-phenylindoline

General Procedure C: To an oven-dried microwave vial equipped with a stir bar under a argon

atmosphere inside a glove box was added Pd(dba)$_2$ (5.8 mg, 0.01 mmol), 2,2'-bis(diphenylphosphino)-1,1'-binaphthyl (9.3 mg, 0.015 mmol), NaO*t*-Bu (26.9 mg, 0.28 mmol) toluene (1.8 mL), and the corresponding amine (0.20 mmol). The microwave vial was sealed and removed from the glove box. The reaction mixture was heated to 110 ℃ in an oil bath and stirred for 12 h. The sealed vial was cooled to room temperature, opened to air, and then 5 drops water was added. The reaction mixture was passed through a short pad of silica gel, rinsed with an additional 6 mL of ethyl acetate (3 × 2 mL), and the combined solutions were concentrated *in vacuo*. The crude material was loaded onto a column of silica gel to give the purified product.

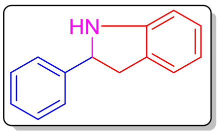

2-Phenylindoline (4ag) The reaction was performed following the General Procedure C with 2-(2-bromophenyl)-1-phenylethan-1-amine **(3ag)** (55.2 mg, 0.20 mmol), Pd(dba)$_2$ (5.8 mg, 0.01 mmol), 2,2'-bis(diphenylphosphino)-1,1'-binaphthyl (9.3 mg, 0.015 mmol), NaO*t*-Bu (26.9 mg, 0.28 mmol) and toluene (1.8 mL). The crude product was purified by chromatography on silica gel (eluted with Petroleum ether:EtOAc = 10:1) to give the desired product (32.3 mg, 83% yield) as a white solid. R_f = 0.23 (Petroleum ether:EtOAc = 10:1). ^{1}H NMR (400 MHz, CDCl$_3$) δ: 7.42 – 7.39 (m, 2H), 7.35 – 7.22 (m, 3H), 7.09 – 7.04 (m, 2H), 6.75 – 6.65 (m, 2H), 4.93 (t, *J* = 9.0 Hz, 1H), 4.12 (s, 1H), 3.42 (dd, *J* = 15.6, 9.2, Hz, 1H), 2.97 (dd, *J* = 15.6, 8.8, Hz, 1H) ppm. ^{13}C{^{1}H} NMR (101 MHz, CDCl$_3$) δ: 151.1, 144.7, 128.7, 128.2, 127.6, 127.5, 126.4, 124.7, 118.9, 108.9, 63.6, 39.7 ppm. The spectroscopic data for this product match the literature data.[8]

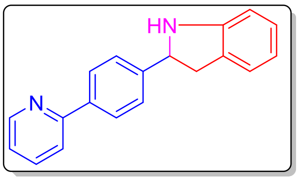

2-(4-(Pyridin-2-yl)phenyl)indoline (4vg) The reaction was performed following the General Procedure C with 2-(2-bromophenyl)-1-(3-(phridin-2-yl)phenyl)ethan-1-amine **(3vg)** (70.6 mg, 0.20 mmol), Pd(dba)$_2$ (5.8 mg, 0.01 mmol), 2,2'-bis(diphenylphosphino)-1,1'-binaphthyl (9.3 mg, 0.015 mmol), NaO*t*-Bu (26.9 mg, 0.28 mmol) and toluene (1.8 mL). The crude product was purified by chromatography on silica gel (eluted with Petroleum ether:EtOAc = 15:1) to give the desired product (48.9 mg, 90% yield) as a yellow oil. R_f = 0.81 (Petroleum ether:EtOAc = 15:1). ^{1}H NMR (400 MHz, CDCl$_3$) δ: 8.70 – 8.68 (m, 1H), 7.99 – 7.96 (m, 2H), 7.77 – 7.70 (m, 2H), 7.55 – 7.53 (m, 2H), 7.24 – 7.21 (m, 1H), 7.12 – 7.07 (m, 2H), 6.77 – 6.69 (m, 2H), 5.02 (t, *J* = 9.1 Hz, 1H), 4.22 (s, 1H), 3.48 (dd, *J* = 15.6, 9.2 Hz, 1H), 3.01 (dd, *J* = 8.8, 15.7 Hz, 1H) ppm. ^{13}C{^{1}H} NMR (101 MHz, CDCl$_3$) δ: 157.3, 151.1, 149.8, 145.6, 138.7, 136.9, 128.2, 127.7, 127.3, 126.9, 124.8, 122.1, 120.6, 119.0, 109.0, 63.4, 39.8 ppm. IR (neat): 3373, 3051, 1608, 1588, 1484, 1466, 1435, 1404, 1045, 754, 703 cm^{-1}. HRMS: calcd for $C_{19}H_{16}N_2$ $[M+H]^+$ 273.1392, found 273.1390.

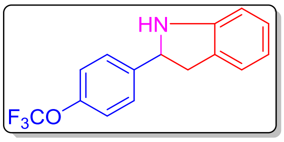

2-(4-(Trifluoromethoxy)phenyl)indoline (4ng) The reaction was performed following the General Procedure C with 2-(2-bromophenyl)-1-(4-(trifluoromethoxy)phenyl)ethan-1-amine **(3ng)** (72.0 mg, 0.20 mmol), Pd(dba)$_2$ (5.8 mg, 0.01 mmol), 2,2'-bis(diphenylphosphino)-1,1'-binaphthyl (9.3 mg, 0.015 mmol), NaO*t*-Bu (26.9 mg, 0.28 mmol) and toluene (1.8 mL). The crude product was purified by chromatography on silica gel (eluted with Petroleum ether:EtOAc = 15:1) to give the desired product (44.6 mg, 80% yield) as a yellow oil. R_f = 0.53 (Petroleum ether:EtOAc = 15:1). ^{1}H NMR (400 MHz, CDCl$_3$) δ: 7.48 – 7.45 (m, 2H), 7.21 – 7.18 (m, 2H), 7.11 – 7.07 (m, 2H), 6.79 – 6.75 (m, 1H), 6.70 – 6.68 (m, 1H), 4.97 (t, *J* = 9.1 Hz, 1H), 4.16 (s, 1H), 3.46 (dd, *J* = 15.6, 9.2 Hz, 1H), 2.96 (dd, *J* = 15.6, 9.0, Hz, 1H) ppm. ^{13}C{^{1}H} NMR (101 MHz, CDCl$_3$) δ: 150.9, 148.5 (q, $J^2_{C\text{-}F}$ = 1.8 Hz), 143.5, 127.86, 127.82, 124.8, 121.7 (q, $J^1_{C\text{-}F}$ = 257.9 Hz), 121.3, 119.2, 109.1, 100.2, 63.0, 39.8

ppm. IR (neat): 3364, 3049, 1608, 1587, 1561, 1484, 1466, 1435, 1405, 1293, 1248, 1152, 749 cm^{-1}. HRMS: calcd for $C_{15}H_{12}F_3NO$ $[M+H]^+$ 280.0949, found 280.0950.

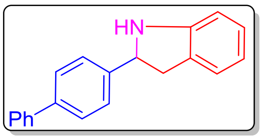

2-([1,1'-Biphenyl]-4-yl)indoline (4qg) The reaction was performed following the General Procedure C with 2-(2-bromophenyl)-1-phenylethan-1-amine **(3qg)** (70.4 mg, 0.20 mmol), $Pd(dba)_2$ (5.8 mg, 0.01 mmol), 2,2'-bis(diphenylphosphino)-1,1'-binaphthyl (9.3 mg, 0.015 mmol), NaO*t*-Bu (26.9 mg, 0.28 mmol) and toluene (1.8 mL). The crude product was purified by chromatography on silica gel (eluted with Petroleum ether:EtOAc = 15:1) to give the product (43.9 mg, 81% yield) as a white solid. Mp 104–105 °C. R_f = 0.53 (Petroleum ether:EtOAc = 15:1). ^1H NMR (400 MHz, $CDCl_3$) δ: 7.60 – 7.56 (m, 4H), 7.51 – 7.42 (m, 4H), 7.37 – 7.33 (m, 1H), 7.12 – 7.07 (m, 2H), 6.77 – 6.73 (m, 1H), 6.70 – 6.69 (m, 1H), 5.01 (t, *J* = 9.0 Hz, 1H), 4.19 (s, 1H), 3.48 (dd, *J* = 15.6, 9.1 Hz, 1H), 3.04 (dd, *J* = 15.6, 8.8 Hz, 1H) ppm. $^{13}C\{^1H\}$ NMR (101 MHz, $CDCl_3$) δ: 151.1, 143.8 140.9, 140.5, 128.9, 128.2, 127.7, 127.45, 127.38, 127.2, 126.9, 124.7, 118.9, 108.9, 63.4, 39.7 ppm. IR (neat): 3364, 3049, 3030, 3011, 2949, 2898, 2844, 1608, 1587, 1561, 1484, 1466, 1435, 1405, 1248, 1152, 1045, 1015, 989, 929, 848, 781, 749, 702cm^{-1}. HRMS: calcd for $C_{20}H_{17}N$ $[M+H]^+$ 272.1395, found 272.1396.

Further Transformations of 1,2-diphenylethylamine

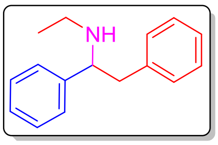

***N*-Ethyl-1,2-diphenylethan-1-amine (5a)** To an oven-dried flask equipped with a stir bar under an argon atmosphere was added 1,2-diphenylethan-1-amine **(3aa)** (38.6 μL, 0.2 mmol) and anhydrous DMF (0.5 mL). Then 1-bromoethane (22.4 μL, 0.3 mmol) was added dropwise over 10 min at room temperature. After the addition was finished, the reaction mixture was stirred at the same temperature for 12 h. The solvent was concentrated *in vacuo* and the crude product was purified by chromatography on silica gel (eluted with Petroleum ether:EtOAc:Et_3N = 100:20:5) to give the product (33.8 mg, 75% yield) as a yellow oil. R_f = 0.52 (Petroleum ether:EtOAc:Et_3N = 100:20:5). ^1H NMR (400 MHz, $CDCl_3$) δ: 7.30 – 7.10 (m, 10H), 3.86 (dd, *J* = 7.8 Hz, *J* = 6.1 Hz, 1H), 2.93 – 2.90 (m, 2H), 2.47 – 2.35 (m, 2H), 0.98 (t, *J* = 7.1 Hz, 3 H), ppm. $^{13}C\{^1H\}$ NMR (101 MHz, $CDCl_3$) δ: 140.0, 139.0, 129.4, 128.5, 128.4, 127.4, 127.1, 126.4, 65.0, 45.3, 42.1, 15.4 ppm. The spectroscopic data for this product match the literature data.[9]

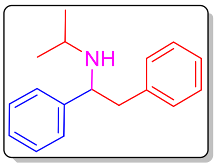

***N*-(1,2-Diphenylethyl)propan-2-amine (5b)** To an oven-dried flask equipped with a stir bar under argon atmosphere was added 1,2-diphenylethan-1-amine **(3aa)** (38.6 μL, 0.2 mmol), anhydrous Na_2SO_4 (43 mg, 0.3 mmol) and acetone (0.8 mL) in sequence at room temperature. The reaction mixture was stirred at the same temperature for 12 h. Then the resulting mixture was filtered and the filtrate was removed *in vacuo* to give the crude product, which was used in the next step without further purification. To a stirred solution of the crude product in MeOH (1 mL) was added $NaBH_4$ (10 mg, 0.26 mmol) at room temperature. The reaction mixture was stirred at room temperature for 1 h, then 5 drops water were added. The reaction mixture was passed through a short pad of silica gel, the silica gel rinsed with an addition 6 mL of ethyl acetate (3 × 2 mL), and the combined solutions were concentrated *in vacuo*. The crude product was purified by chromatography on silica gel (eluted with Petroleum ether:EtOAc:Et_3N = 100:20:5) to give the desired product (35.1 mg, 82% yield) as a yellow oil. R_f = 0.52 (Petroleum ether:EtOAc:Et_3N = 100:20:5). ^1H NMR (400 MHz, $CDCl_3$) δ: 7.31 – 7.16 (m, 8H), 7.09 – 7.06 (m, 2H), 3.99 (t, *J* = 7.0 Hz, 1H), 2.91 – 2.89 (m, 2H), 2.60 – 2.54 (m, 1H), 0.94 (d, *J* = 6.4 Hz, 3H), 0.90 (d, *J* = 6.2 Hz, 3H) ppm. $^{13}C\{^1H\}$ NMR (101 MHz, $CDCl_3$) δ: 144.3, 139.0, 129.4, 128.4, 128.3, 127.4, 127.0, 126.3, 61.9, 45.8, 45.7, 24.4, 22.0 ppm.

IR (neat): 3398, 2955, 2924, 2854, 1602, 1494, 1453, 1378, 699 cm^{-1}. HRMS: calcd for $C_{17}H_{21}N$ $[M+H]^+$ 240.1708, found 240.1713.

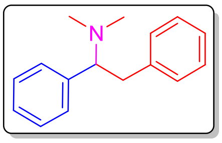

***N*,*N*-Dimethyl-1,2-diphenylethan-1-amine (5c)** To an oven-dried microwave vial equipped with a stir bar under an argon atmosphere was added 1,2-diphenylethan-1-amine **(3aa)** (38.6 µL, 0.2 mmol), 37 % aqueous formaldehyde (113 µL, 1.2 mmol) and formic acid (87 µL, 2 mmol) in sequence at room temperature. The reaction mixture was refluxed for 24 h. After cooling to room temperature, NaOH (1 M) was added until the pH=10. The solution was extracted with dichloromethane (3 × 5 mL). The combined organic layers were washed with brine, dried over anhydrous Na_2SO_4, filtered and concentrated *in vacuo*, the crude product was purified by chromatography on silica gel (eluted with Petroleum ether:EtOAc:Et_3N = 100:20:5) to give the desired product (23.6 mg, 52%) as a yellow oil. R_f = 0.52 (Petroleum ether:EtOAc:Et_3N = 100:20:5). ^1H NMR (400 MHz, $CDCl_3$) δ: 7.25 – 7.16 (m, 3H), 7.14 – 7.05 (m, 5H), 6.95 – 6.92 (m, 2H), 3.44 (dd, *J* = 9.8 Hz, *J* = 5.0 Hz, 1H), 3.31 (dd, *J* = 13.2 Hz, *J* = 5.0 Hz, 1H), 2.95 (dd, *J* = 14.0 Hz, *J* = 9.7 Hz, 1H), 2.26 (s, 6H), ppm. $^{13}C\{^1H\}$ NMR (101 MHz, $CDCl_3$) δ: 139.7, 139.6, 129.4, 128.9, 128.0, 128.0, 127.1, 125.9, 72.9, 43.1, 40.1 ppm. The spectroscopic data for this product match the literature data.[9]

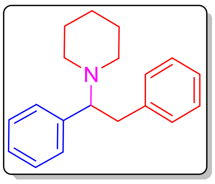

1-(1,2-Diphenylethyl)piperidine (5d) To an oven-dried microwave vial equipped with a stir bar under an argon atmosphere inside a glove box was added K_2CO_3 (165.6 mg, 1.2 mmol), anhydrous CH_3CN (0.5 mL) and 1,2-diphenylethan-1-amine **(3aa)** (38.6 µL, 0.2 mmol). The microwave vial was sealed and removed from the glove box. Then 1,5-dibromopentane (82 µL, 0.3 mmol) was added dropwise over 10 min via syringe at room temperature. After the addition was finished, the reaction mixture was stirred at the same temperature for 3 days. The potassium salts were removed by filtration and washed with CH_3CN (3 × 5 mL). The filtrate was concentrated *in vacuo* and the residue was purified by flash chromatography (eluted with Petroleum ether:EtOAc:Et_3N = 100:20:5) to give the desired product (43.2 mg, 81% yield) as a yellow oil. R_f = 0.52 (Petroleum ether:EtOAc:Et_3N = 100:20:5). ^1H NMR (400 MHz, $CDCl_3$) δ: 7.26 – 7.16 (m, 3H), 7.15 – 7.05 (m, 5H), 7.00 – 6.97 (m, 2H), 3.56 (dd, *J* = 9.5 Hz, *J* = 5.1 Hz, 1H), 3.30 (dd, *J* = 13.4 Hz, *J* = 5.2 Hz, 1H), 3.00 (dd, *J* = 13.3 Hz, *J* = 9.5 Hz, 1H), 2.47 – 2.40 (m, 4H), 1.63 – 1.49 (m, 4H), 1.41 – 1.33 (m, 2H), ppm. $^{13}C\{^1H\}$ NMR (101 MHz, $CDCl_3$) δ: 140.1, 139.5, 129.5, 129.1, 128.0, 127.8, 127.0, 125.8, 72.5, 51.5, 39.3, 26.5, 24.8 ppm. The spectroscopic data for this product match the literature data.[10]

Supplementary References

1 Haak, E., Siebeneicher, H. & Doye, S. An ammonia equivalent for the dimethyltitanocene-catalyzed intermolecular hydroamination of alkynes. *Org. Lett.* **2**, 1935-1937 (2000).

2 Gyenes, F., Bergmann, K. E. & Welch, J. T. Convenient access to primary amines by employing the Barbier-type reaction of N-(trimethylsilyl) imines derived from aromatic and aliphatic aldehydes. *J. Org. Chem.* **63**, 2824-2828 (1998).

3 Wilckens, K., Lentz, D. & Czekelius, C. Synthesis of gold complexes bearing sterically highly encumbered, chiral carbene ligands. *Organometallics* **30**, 1287-1290 (2011).

4 Das, M. & O'Shea, D. F. Highly selective addition of a broad spectrum of trimethylsilane pro-nucleophiles to N-*tert*-butanesulfinyl imines. *Chem. - Eur. J.* **21**, 18717-18723 (2015).

5 Maury, J., Zawodny, W. & Clayden, J. Stereospecific intramolecular arylation of 2- and 3-pyridyl substituted alkylamines via configurationally stable α-pyridyl organolithiums. *Org. Lett.* **19**, 472-475 (2017).

6 Pallavicini, M., Bolchi, C., Moroni, B., Valoti, E. & Piccolo, O. Resolution of 1-phenyl-2-(p-tolyl)ethylamine via diastereomeric salt formation. *Tetrahedron Asymmetry* **14**, 2247-2251 (2003).

7 Roe, C., Hobbs, H. & Stockman, R. A. One-pot synthesis of chiral nonracemic amines. *J. Org. Chem.* **76**, 9452-9459, (2011).

8 Villanueva, O., Weldy, N. M., Blakey, S. B. & MacBeth, C. E. Cobalt catalyzed sp^3 C-H amination utilizing aryl azides. *Chem. Sci.* **6**, 6672-6675 (2015).

9 Xie, L.-G. & Dixon, D. J. Tertiary amine synthesis via reductive coupling of amides with Grignard reagents. *Chem. Sci.* **8**, 7492-7497 (2017).

10 Hesp, K. D. & Stradiotto, M. Stereo- and regioselective gold-catalyzed hydroamination of internal alkynes with dialkylamines. *J. Am. Chem. Soc.* **132**, 18026-18029 (2010).
